# Identification of Novel HLA-A*0201-Restricted CTL Epitopes in Chinese Vitiligo Patients

**DOI:** 10.1038/srep36360

**Published:** 2016-11-08

**Authors:** Tingting Cui, Xiuli Yi, Sen Guo, Fubo Zhou, Ling Liu, Chunying Li, Kai Li, Tianwen Gao

**Affiliations:** 1 Department of Dermatology, Xijing Hospital, Fourth Military Medical University, 127 Changle West Road, Xi’an, shaanxi, 710032, China

## Abstract

Generalized vitiligo is an autoimmune disease characterized by melanocyte loss, which results in patchy depigmentation of skin and hair. Recent studies suggested the key role of CD8^+^T lymphocytes for mediating immune response in vitiligo through melanocyte differentiation antigens, including tyrosinase, gp100 and MelanA/Mart-1. However, the specific epitopes of these auto-antigens are still unknown. In our study, we predicted the possible HLA-A*0201-restricted nonapeptides overlaying the full-length amino acid sequences of these three known antigens and investigated the lymphocytes reactivity to these nonapeptides by Elispot assay. In addition, we evaluated the abilities of these nonapeptides to activate CD8^+^T cells. We screened out 5 possible epitopes originated from tyrosinase and gp100, numbered P28, P41, P112, P118 and P119. Among these 5 epitopes, notably, P28 and P119 played the dominant role in activating CTLs, with a significant increase in proliferation rate and Interferon-γ (IFN-γ) production of CD8^+^T cells. Nevertheless, antigen-specific T cell reactivity was not detected in MelanA/Mart-1 peptides. Our studies identified two novel epitopes originated from proteins of gp100 and tyrosinase, which may have implications for the development of immunotherapies for vitiligo.

Generalized vitiligo is an autoimmune disease characterized by melanocyte loss, which results in patchy depigmentation of skin and hair. Psychological symptoms are more aggravated in the young vitiligo patients^1^. Various factors are implicated in the pathogenesis of vitiligo, such as oxidative stress^2^, genetic factors^3^,^4^, and T lymphocytes mediated melanocytes destruction^5^, but none of these can fully clarify the mechanism of vitiligo. However, accumulating evidences emphasized the crucial role of CD8^+^T lymphocytes (CD8^+^T cells) in the destruction of melanocytes. Previous studies demonstrated that CD8^+^T-cell counts in both lesional skin and the blood of vitiligo patients is significantly increased^6^, and the CD4^+^/CD8^+^ ratio is decreased in active vitiligo patients than that in stable patients^7^, which suggested that the CD8^+^T cells mediated autoimmune response involved in the pathogenesis and progression of vitiligo. Studies on immunophenotypic characterization of lymphoid cell infiltration in vitiligo further suggested that the autoimmune reactivity towards melanocyte antigens might be T-cell-dependent and antigen-driven^6^,^8^.

Auto-antigens in pathological situation, such as oxidative stress, may abnormally expressed or be partially modified to produce new peptide sequences^9^. These aberrant expression or modifications peptides turn into auto-antigens, if these peptides are presented by antigen-presenting cells, and recognized by CD8^+^T cells, T cell response will be initiated. The vast majority of circulating CD8^+^T cells express αβ cell receptors (TCRs), which play integral role in the adaptive immune response by recognizing peptides bound to major histocompatibility complex class I (MHC I) molecules^10^. Therefore, formation of auto-antigens is the critical factor to initiate the autoimmune disease. Importantly, autoreactive T cells with a very low affinity can escape negative selection in the thymus^11^,^12^, this subset of T cells could activated by auto-antigens to initiate autoimmune disease^13^. For autoreactive CD8^+^T cells, to execute their function, they must differentiate into cytotoxic T lymphocytes (CTLs), a process that is initiated following stimulation with specific antigens. In recent years, several auto-antigens, especially the melanocyte differentiation antigens, including gp100^14^, tyrosinase^15^ and melanA/MART-1^16^, have been proved to promote T lymphocytes activation and differentiation in vitiligo^17^,^18^,^19^.

In the vitiligo pathogenesis, auto-antigens are mainly melanocyte differentiation proteins, which merely expressed in melanocytes and melanomas. Previous studies have demonstrated that tyrosinase, gp100 and melanA/Mart-1were involved in the process of melanin synthesis and melanin body transportation in the physiological situation, but in pathological situation, they might be exposed outside of the cell membrane of melanocytes, and might produce new auto-antigens that initiated the autoimmune disease^20^. In vitiligo, several epitopes originated from these three proteins have been demonstrated to trigger the T cell response in different races. However, due to different populations and sample sizes, conflicting results have been obtained from these studies regarding T cell responses against the same epitopes^8^,^17^,^21^. In addition, in the previous studies, only limited epitopes from the three auto-antigens were analyzed. Whether there are any other novel epitopes deriving from these auto-antigens are still unknown.

In this study, we aimed to find new epitopes from the melanocyte differentiation antigens in HLA-A*0201 positive Han Chinese vitiligo patients. Firstly, we predicted all the possible HLA-A*0201 restricted epitopes of the three melanocyte differentiation antigens by online software, screened out the candidate peptides by Enzyme linked immunospot assay (Elispot assay) through the signaling of IFN-γ spots forming. Moreover, to explore the immunogenetic properties of these epitopes, we tested the proliferation rate and cytokines production of CD8^+^T cells after co-culture of the candidate peptides and peripheral blood mononuclear cells (PBMCs) isolated from vitiligo patients. Our studies identified two novel epitopes originated from proteins of gp100 and tyrosinase, which may contribute to the immunotherapy of vitiligo.

## Results

### Identification of HLA-A*0201 restricted epitopes originated from Tyrosinase, gp100 and MelanA/Mart-1

A total of 170 peptides were predicted, about every 10 peptides were mixed to form a pool. Firstly, the reactivity of PBMCs to peptide pools were tested by Elispot assay, and the pools with higher spot forming cells (SFC) were screened out as positive pools. In order to sort out the specific peptide epitopes, peptides included in positive pools were further analyzed respectively and the candidate peptides were identified.

Among the 170 peptides, 51 peptides were derived from tyrosinase, 106 peptides from gp100, and 13 peptides from MelanA/Mart-1, the peptides were numbered from P1 to P169, and the corresponding sequences are listed in Tables S1–S3. Furthermore, approximately every 10 peptides were mixed to form a peptide pool, a total of 18 peptide pools are generated and numbered from M1 to M18. The peptides included in each pool are listed in Table S4.

**Figure 1:**
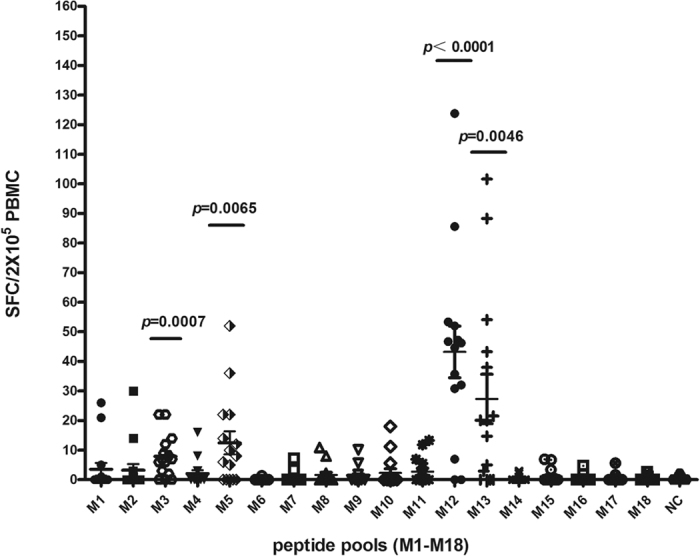
Positive peptide pools were screened by elispot assay through the signaling of SFC. The SFC values were significantly increased after co-culture of PBMCs with the peptide pools of M3 (*P* = 0.0007 versus control group), M5 (*P* = 0.0065 versus control group), M12 (*P* < 0.0001 versus control group) and M13 (*P* = 0.0046 versus control group), no significant difference were seen in other peptide pools (n = 15).

The PBMCs isolated from 15 patients (HLA-A*0201) were used for testing the reactivity to each peptide pools. The results showed that the SFC was significantly elevated after co-culture of PBMCs with peptide pool M3, M5, M12 and M13, whereas no obvious changes were detected in any other pools compared with control groups (Fig. 1). The results indicated that in each pool there was at least one specific epitopes which can be recognized by PBMCs, and could futher activate PBMCs to secrete IFN-γ. This result suggested that the specific epitopes were included in M3, M5, M12 or M13.

**Figure 2:**
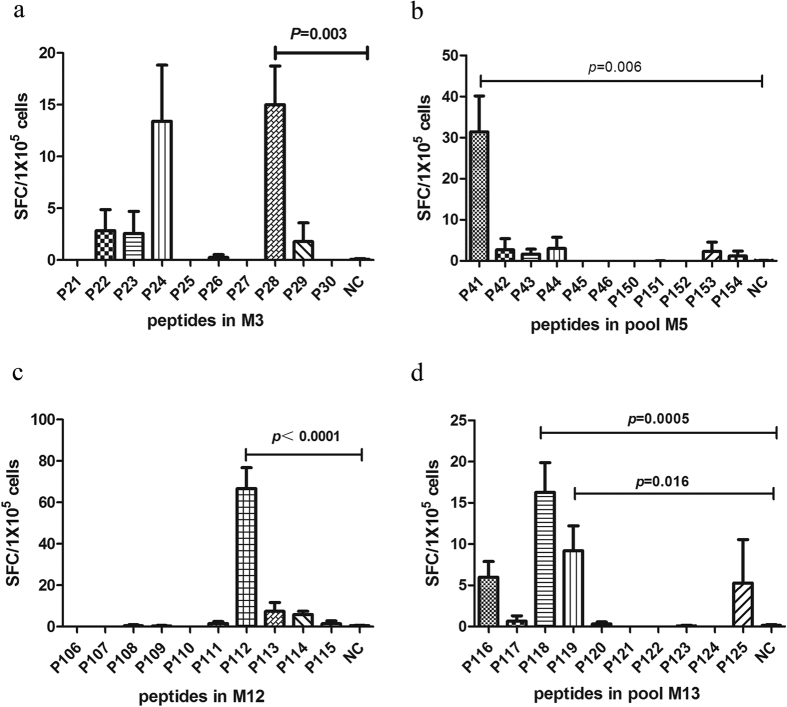
Candidate epitopes were identified by elispot assay through the signaling of SFC. (**a**) P28 in M3 was identified as candidate epitope (*P* = 0.003 versus control group). (**b**) P41 in M5 was identified as candidate epitope (p = 0.006). (**c**) P112 in M12 was identified as candidate epitope (*P* < 0.0001 versus control group). (**d**) P118 and P119 in M13 were identified as candidate epitopes (*P* = 0.0005, *P* = 0.016 versus control group), (n = 15).

To further find out the specific epitopes which play the key role in activating PBMCs of vitiligo patients, the peptides included in M3, M5, M12 and M13 were tested respectively. When co-culture of the PBMCs with peptide P28, P41, P112, P118 and P119, respectively, the SFC values were significantly elevated compared with irrelevant peptides or other peptides (Fig. 2a–d). Thus, we identified these 5 peptides as candidate epitopes, and the corresponding sequences were listed in Table 1.

### Higher binding affinity of P28 and P119 to MHC I molecules and capability of inducing PBMCs of vitiligo to release IFN-γ

MHC I molecules presented processed antigens in the form of short peptides to CD8^+^T lymphocytes. To evaluate the binding affinity of candidate peptides to CD8^+^T cells, T2 cell lines were employed, T2 cells were characterized by deficiency of transport antigen protein, the expression of HLA-A*0201 molecules was only detected on T2 cells after binding with signal sequence-derived peptides. In this study, after co-culture of T2 cells with our candidate peptides for 24 h, the cells were harvested and the binding affinity was analyzed by flow cytometry through signaling of anti-HLA-DR.

**Figure 3:**
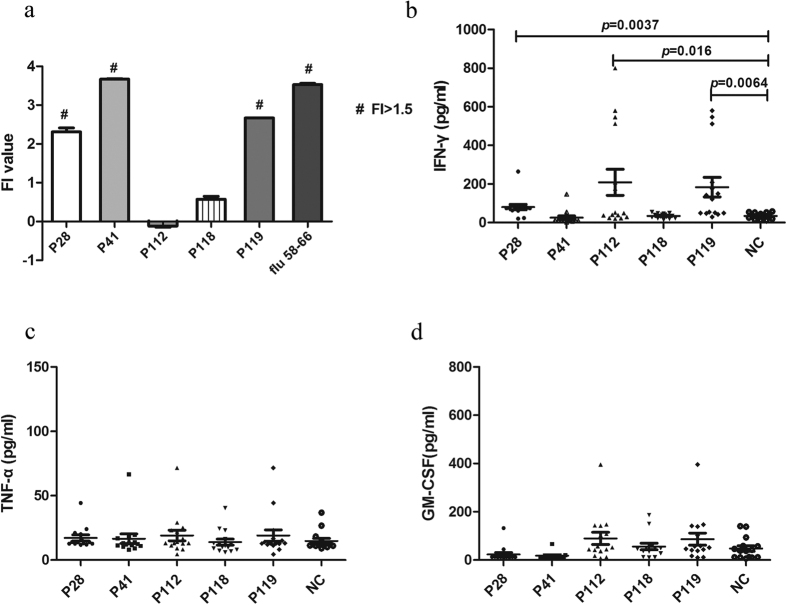
Higher binding affinity of P28 and P119 to MHC I molecules and capability to induce IFN-γ release from PBMCs of vitiligo. (**a**) The binding affinity of candidate epitopes to MHC I molecular were tested by T2 assay. P28, P41 and P119 have the high binding affinity with FI > 1.5, P112 and P118 have low binding affinity with FI < 1.5. (**b**) IFN-γ levels in supernatants after co-culture of PBMCs of vitiligo with each candidate epitopes. The contents were significantly increased in P28 (*P* = 0.0037 versus control group), P112 (*P* = 0.016 versus control group) and P119 (*P* = 0.0064 versus control group) treated groups compared with control group. (**c**) TNF-α levels in supernatants after co-culture of PBMCs of vitiligo with each candidate epitopes. (**d**) GM-CSF levels in supernatants after co-culture of PBMCs of vitiligo with each candidate epitopes.

Notably, 3 peptides, including P28, P41 and P119 had the higher binding affinity (FI > 1.5) to MHC I molecules, whereas the other two peptides (P112 and P118), had lower binding affinity (FI < 1.5, Fig. 3a). This result suggested that 3 of our candidate peptides, P28, P41 and P119 bound MHC I molecules more easily than other peptides and might be candidate epitopes involved in activation of CD8^+^T cells.

Cytokine production was considered to be another marker of T cell activation. As to determine whether these candidate peptides could induce the PBMCs to release cytokines, we detected the IFN-γ, Tumor necrosis factor-α (TNF-α) and Granulocyte-macrophage colony stimulating factor (GM-CSF) contents in the supernatants after co-culture of PBMCs with each candidate peptides respectively.

We found that the IFN-γ contents in the supernatants were significantly increased after co-culture with P28 (*P* < 0.01), P119 (*P* < 0.01) and P112 (*P* < 0.05) compared with the irrelevant peptides (NC), but no obvious changes were detected in P24 and P118-treated groups (Fig. 3b), Futhermore, there were no significant difference on the levels of TNF-α and GM-CSF after incubation with P28 and P119 (Fig. 3c,d). These results suggested that P28 and P119 were capable of inducing T cells to secret cytokines of IFN-γ. Combining with the results of T2 assay, these results strongly proved the immunological competence of P28 and P119.

### P28 and P119 can effectively promote CD8^+^T cells proliferation *in vitro*

As with CTL response, functional epitopes should stimulate the T cell proliferation. To further determine the immunological activities of P28 and P119, we tested the proliferation rate of CD8^+^T cells after co-culture of CD8^+^T cells from vitiligo patients and healthy controls with P28, P119 or irrelevant peptide respectively *in vitro*.

**Figure 4:**
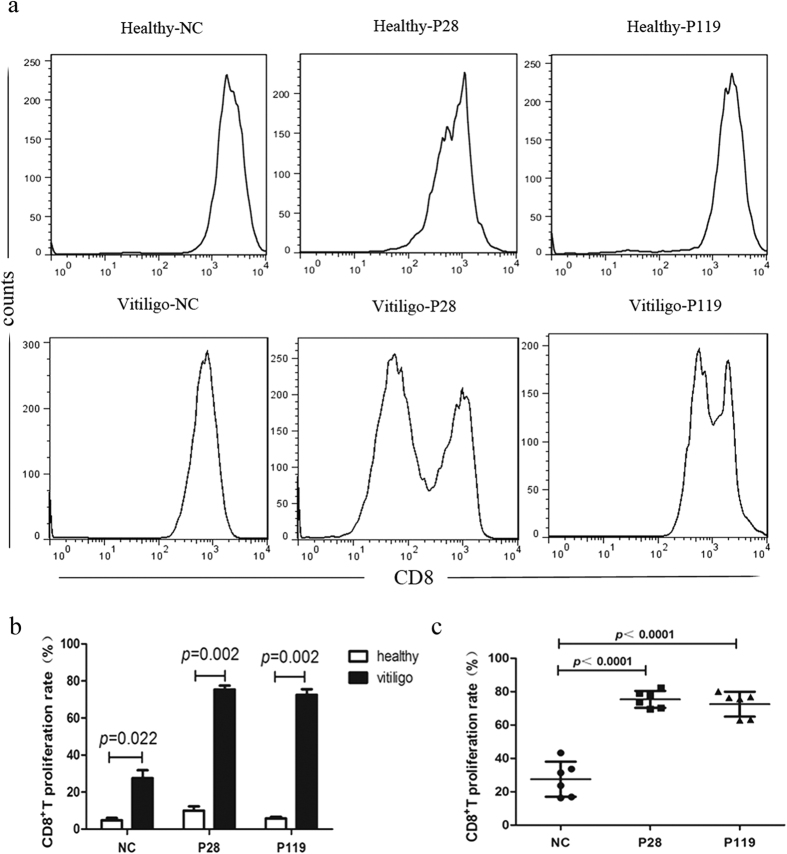
P28 and P119 activated the CD8^+^T cells proliferation *in vitro*. (**a**) Flow cytometry to detect the proliferation rate of CD8^+^T cells from healthy controls and vitiligo patients after co-culture with candidate peptides. (**b**) The proliferation rate of CD8^+^T cells from vitiligo patients were significantly increased compared with healthy controls after stimulation with P28 and P119 (*P* = 0.002 versus healthy controls group for both). (**c**) The proliferation rate of CD8^+^T cells from vitiligo patients were significantly elevated after stimulation with P28 and P119 compared with irrelevant peptide (*P* < 0.0001 versus irrelevant peptide group for both).

As shown in Fig. 4b, the proliferation rate of CD8^+^T cells from vitiligo patients reactive to P28 and P119 significantly increased compared with the healthy controls (*P* = 0.002 versus healthy controls group for both). Furthermore, we compared the proliferation rate of CD8^+^T cells from vitiligo patients reactive to the P28 or P119 peptide with irrelevant pepdide. The results showed that more than 70% of CD8^+^T cells proliferated in response to P28 and P119 stimulation, while only less than 30% of CD8^+^T cells proliferated in response to the irrelevant peptide, the proliferation index of CD8^+^T cells was significantly increased in response to P28 (*P* < 0.0001) and P119 (*P* < 0.0001) (Fig. 4c). These results indicated that P28 and P119 had functional activities to promote proliferation of CD8^+^T cells of vitiligo.

### P28 and P119 can evaluate the intracellular cytokine production of CD8^+^T cells *in vitro*

**Figure 5:**
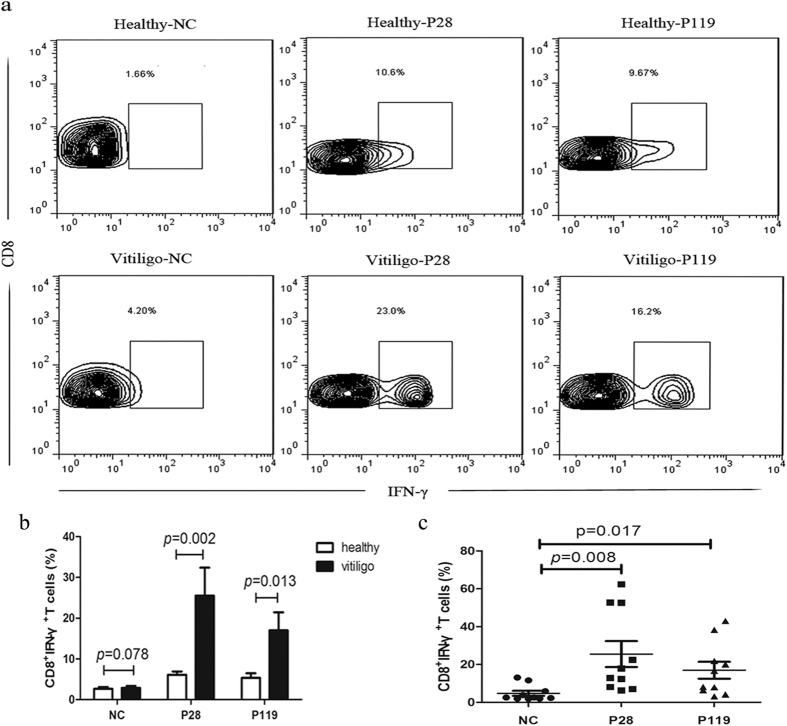
P28 and P119 elevated the CD8^+^T cells to differentiate into functional CTLs *in vitro*. (**a**) Intracellular cytokine staining assay to detect IFN-γ producing CTLs after co-culture of CD8^+^T cells from healthy controls and vitiligo patients with candidate peptides. (**b**) The proportion of IFN-γ^+^CD8^+^T cells from vitiligo patients were significantly elevated compared with healthy controls after stimulation with P28 (*P* = 0.002 versus healthy control group) and P119 (*P* = 0.013 versus healthy control group). (**c**) The proportion of IFN-γ^+^CD8^+^T cells from vitiligo patients were significantly increased after stimulation with P28 and P119 (*P* = 0.008, 0.017 versus irrelevant peptide, respectively) compared with irrelevant peptide.

To accurately measure the percentage of functional CTLs, we further evaluated the intracellular cytokine production of CD8^+^T cells in 10 vitiligo patients and heathy controls by flow cytometry respectively. As shown in Fig. 5b, The proportion of IFN-γ^+^CD8^+^T cells from vitiligo patients were significantly elevated compared with healthy controls after stimulation with P28 (*P* = 0.002) and P119 (*P* = 0.013). Futhermore, the ratio of IFN-γ^+^CD8^+^T cells from vitiligo patients were significantly increased after stimulation with P28 and P119 (*P* = 0.008, 0.017 versus irrelevant peptide, respectively) compared with control pepdide (Fig. 5c). These results indicated that the functional CTLs could be induced by our candidate peptides P28 and P119.

## Discussion

Our finding expands the theoretical basis to seek the immunologic pathogenesis of vitiligo. In this study we demonstrated an increasing frequency of gp100 and tyrosinase antigen-specific CD8^+^T cells in the circulation of HLA-A*0201-positive vitiligo patients. This reactivity further supports the notion that there is a T lymphocytes-mediated component of vitiligo progression. CD8^+^T cell reactivity to gp100 and tyrosinase-originated peptide epitopes (P28 and P119) were significantly higher in vitiligo patients and was marked with CD8^+^T cell proliferation rate significantly increased compared with irrelevant peptides. In addition, the proportion of functional CTLs was also significantly increased, reaching 4–5 folds of that in irrelevant peptide treated group. The CTLs proportion is more closely reflect a truer measurement of an active response to previous encountered antigen. Overall, these results indicate a clear relevance of CD8^+^T lymphocyte reactivity to melanocyte gp100 and tyrosinase in vitiligo, which suggesting a pathogenic role for gp100 and tyrosinase-specific T cells in vitiligo. Otherwise, CD8^+^ T lymphocyte reactivity to MelanA/Mart-1 peptide *ex vivo* was not seen in this vitiligo patient population. These findings suggest that although reactivity to MelanA/Mart-1 may exist in vitiligo patients, it occurs at low levels. These results were the same as previously described by Mandelcorn-Monson^17^. The lower level of reactivity to MelanA/MART-1 may relate to the possible presence of nonreactive melanocyte-specific T cells in vitiligo patients^17^.

Previous studies suggested that class I allele HLA-A2 is associated with vitiligo^22^. It was reported that antigen-specific T lymphocyte reactivy to gp100 peptides was seen in HLA-A2-positive Caucasian patients with vitiligo^17^. Furthermore, Singh *et al*. identified three specific alleles, HLA-A*33:01, HLA-B*44:03, and HLA-DRB1*07:01 to be significantly increased in vitiligo patients as compared with controls in both studies of North Indians an Gujarat cases^23^. The ratio of HLA type might be different in distinct ethnic groups. According to our study in Chinese Han populations, the vitiligo patients with HLA-A*0201 subtype made the maximum proportion (Data were not shown). Therefore, we chose the HLA-A*0201-positive vitiligo patients to perform the experiments in this study. It’s also important that detecting other HLA types of Chinese vitiligo patients in our further study.

Our study was expanding upon previous studies on the identification of specific epitopes recognized by CD8^+^T cells of vitiligo. In this study, we predicted all the possible 170 HLA-A*0201 restricted nonapeptides deriving from gp100 (106 peptides), tyrosinase (51 peptides) and MelanA/Mart-1 (13 peptides). Based on the prediction results, all of the 170 epitopes were synthesized and their abilities to induce IFN-γ production from PBMCs were tested in 15 HLA-A*0201-positive vitiligo patients by Elispot assay. So far, this is the first study that tested immune activities of all the HLA-A*0201 restricted epitopes originated from these auto-antigens in vitiligo patients, and we identified two novel critical epitopes, P28 and P119, which played dominant roles in activating CD8^+^T cells, thus leading to the development of vitiligo pathogenesis.

Epitopes specific-PBMCs also supporting the T cell activation, and on the other side inducing cytotoxic T cell to kill melanocytes *in vivo*. The differences of cell fate are dependent on its differentiation into functional CTLs. Here, we show that our identified two novel peptides bound to MHC I molecules trigger the expansion of epitopes-specific CTLs in vitiligo patients. For this reason, we suggested that these two epitopes might have the potential abilities to function *in vivo*.

In the past decades, a variety of melanocytes-expressing proteins have been proved to participant in cellular immunity of vitiligo. Besides of gp100, tyrosinase and MelanA/Mart-1, the autoantibodies to tyrosinase-related protein 1 and 2 (TRP1 and TRP2)^24^,^25^, tyrosine hydroxylase^26^, lamin A^27^, HSP70^28^ were detected in the serum of vitiligo. Many researchers attempted to find the accurate epitopes in these auto-antigens, but owing to the limitation on patient number and population, conflicting results were obtained. Previous studies demonstrated that T cells isolated from 9 perilesional skin biopsies and autologous PBMC showed similar increases in melanocyte antigen recognition^29^. Elispot assays in another study demonstrated 88% of HLA-A*02 positive patients showed reactivity to gp100-originated and modified epitopes, gp100 209–217, 210 M and gp100 280–288, 288 V, but T cell reactivity to tyrosinase or MelanA/Mart-1 peptides were not detected^17^. Lang *et al*. reported that after testing T cell reactivity to peptides from gp100, MelanA/MART1, and tyrosinase, they found MelanA/Mart-1 peptides were immunodominant in nine patients reacting against EAAGIGILTV and three patients reacting against ILTVILGVL. Furthermore, they indicated that 70% patients with actively progressive disease showed CD8^+^T cell reactivity to the epitopes vs those of 18% patients with moderate disease activity^19^. These studies were partially consistent with our findings, besides of a nonapeptide originated from gp100, P119 (gp100 585–593), we also identified another nonapeptide P28 (tyrosinase 343–351) which was originated from tyrosinase. Whereas, another previous study failed to detect circulating antigen-specific T-cell responses to tyrosinase, gp100, Melan-A, and TRP-2 in vitiligo patients, and multimer staining in this same study only detected low or borderline frequencies of Mart-1^+^ CD8^+^ T cells in vitiligo patients^21^. Besides the above studies, other articles also obtained conflicting results regarding the frequency of melanocyte-specific cytotoxic T lymphocytes in vitiligo patients^18^,^29^.

Several reasons may account for the discrepancy between theses studies. First, the circulating CD8^+^T cells may be more active in patients at the progressive stage, therefore, we excluded the subjects at the stable stage. Second, patients from previous studies were mostly from Caucasians, while we focused on the Chinese population, we hypothesized that antigenic-specific T cell responses might react differently even to the same epitopic peptides in different populations.

Our findings may also have implications for the development of immunotherapies for vitiligo and melanoma. Casares *et al*. found that systemic delivery of nanoparticles coated with autoimmune-disease-relevant peptides bound to MHC II molecules triggered antigen-specific regulatory CD4^+^T cell type 1-like cells in different mouse models, including mice humanized with lymphocytes from patients, leading to resolution of established autoimmune phenomena^30^. If the dominant epitopes that mediate the occurrence of vitiligo can be identified, it is possible to develop therapies to treat this disease using the approach above. Many studies have shown that among melanoma patients receiving immunotherapy, definitive anti-tumor responses are accompanied with high levels of circulating antigen-specific CD8^+^T cells^31^,^32^,^33^,^34^. If the specific epitopes can be identified, induction or augmentation of T cell responses to the specific epitopes to induce vitiligo might be an effective therapy for melanoma.

In summary, we found 5 accurate epitopes that can induce the activation of PBMCs from vitiligo patients in HLA-A*0201 positive Han Chinese population. Furthermore, we identified two dominant epitopes, gp100 343–351 and tyrosinase 585–593, which could activate the CD8^+^T cells to be functional CTLs. Our findings further contribute to the immunopathologic mechanism in vitiligo, in which cell-mediated responses to normal melanocyte antigens plays a crucial part. Further studies are needed to discuss whether these eiptopes can be applied in treatment of vitiligo or melanoma in animal models.

## Material and Methods

### Study subjects

Generalized vitiligo patients were recruited from Xijing Hospital, Fourth Military Medical University. For consideration of T lymphocytes activities, only patients in progressive stage and with depigmentation area more than 5% of the body surface were enrolled in this study. All vitiligo patients did not receive any systemic treatment for at least 3 months prior to the procedures, including immunosuppressive agents or phototherapy. Information on demographics and other characteristics were obtained with questionnaires. A total of 77 patients were enrolled in this study and the corresponding information were summarized in Table 2, in which, 15 patients were used for testing the reactivity to each peptide pools, another 15 patients were used for testing reactivity to the specific peptide by Elispot assays. 15 patients were used for testing cytokine production. Besides, 6 and 10 patients were used for testing the T cell proliferation and intracellular cytokines detection for IFN-γ respectively. All the subjects voluntarily agreed to participate in this study and signed informed consent forms, each of them donated 20 ml of blood, which was collected in heparinized tubes for separation of PBMC and extraction of DNA. This study was approved by the ethics review board of Fourth Military Medical University and was conducted according to the principles of Helsinki Declaration.

### Lymphocytes preparation

The Peripheral Blood Mononuclear Cells (PBMCs) were isolated from 20 ml of blood samples using human lymphocyte separation medium (Dakewe, Beijing, China) according to the the manufacturer’s recommendations. For T cell proliferation assay and cytokines production assay, the isolated PBMCs were freshly cultured in RPMI-1640 containing 20% fetal bovine serum. For elispot assay, the PBMCs were frozen in fetal bovine serum with 10% dimethyl sulfoxide (DMSO) and stored in liquid nitrogen until enough samples were collected.

### DNA extraction and HLA genotyping

Genomic DNA was extracted from 1 ml fresh blood by using RelaxGene Blood DNA System kit (Tiangen Biotech, Beijing, China) according to the instruction. DNA quality was confirmed by 1% agarose gel electrophoresis. HLA genotyping were performed by Songon Biotech company (Shanghai, China) using PCR-SBT. About a third of the subjects have been confirmed to carry HLA-A*0201allele in Han Chinese population, and the PBMCs isolated from these patients were used for further analysis.

### Epitope prediction and peptide synthesis

The online software BIMAS (http://www-bimas.cit.nih.gov/molbio/hla_bind/) and SYFPEITHI (http://www.syfpeithi.de/bin/MHCServer.dll/EpitopePrediction.htm) were applied to predict the possible HLA-A*0201-restricted antigenic epitopes originated from tyrosinase, gp100, and MART-1. The details were provided in Supplementary data 1 (data S1). A total of 170 epitope peptides were finally chosen for analysis, in which 51 peptides were derived from tyrosinase, 106 peptides from gp100 and 13 peptides from MART-1. The HIV p17Gag protein derived peptide SLYNTVATL was used as negative control, which cannot cause a specific reaction in anti-melanocyte T cells^17^. All the peptides were synthesized by Sangon Biotech company (Shanghai, China), and were confirmed by reverse phase-high performance liquid chromatography (Supplementary data 2, data S2).

### Elispot assay

IFN-γ ELISPOT assays were performed using cytokine capture and detection reagents according to the manufacturer’s instructions (ELISpotPRO for human IFN-γ, Mabtech, Stockholm, Sweden). Briefly, 96-well nitrocellulose plates pre-coated with anti- IFN-γ mAb were seeded with PBMCs at a density of 2 × 10^5^ cells in 200 μl medium per well, followed by addition of 4 μl (1 μg/μl) peptides or 40 μl mixed peptide (pools), the final concentration of each peptide is 4 μg/well, CD3 antibody was used as positive control in a dilution of 1:1000. After incubation for 48 h at 37 °C, the cells were discarded, and captured IFN-γ was detected with a biotinylated anti- IFN-γ Ab, followed by addition of an alkaline phosphatase substrate solution (BCIP/NBT-plus). Reaction was stopped by washing extensively in tap water until distinct spots emerged, and spots were counted using an ELISPOT Image Analyzer and software (Cell Technology Inc. Jessup, MD). The spots forming cells (SFC) are defined as: SFC = experimental group- negative control group.

### Elisa assay for detection of cytokines production

To quantify the concentration of cytokines in the supernatants after co-culture of T lymphocytes and peptides, enzyme-linked immune sorbent assay (Elisa assay) were performed. The PBMCs were plated in 96 well plates at a density of 2 × 10^6^ in 200 μl medium per well, followed by addition of 20 μl (1 μg/μl) peptides. After incubation for 48 h, the supernatants were collected, and the concentration of IFN-γ, TNF-α and GM-CSF were measured using Microtitre 96-well polystyrene plates (Shanghai’s male technology co., Shanghai, China) according to the manufacturer’s recommendations. Each sample was analyzed in triplicates.

### Peptide binding affinity assay

To evaluate the binding affinity of each candidate peptides to HLA-A*0201 molecules, the classical T2 peptide-binding assay was performed. T2 cells, a cell line characterized by TAP-deficient and HLA-A*0201-positive, were incubated overnight with peptides (100 μM) in PRMI 1640 medium containing 20% fetal bovine serum and β2-microglobulin (3 μg/ml) at 37 °C for 24 h. Then the cells were harvested, washed, and stained with FITC-conjugated anti-HLA-DR mAb for 30 min at 4 °C, the fluorescence intensity was analyzed by flow cytometry. The mean fluorescence index (FI) were calculated as: FI = [mean fluorescence intensity (MFI)_sample_ − MFI_background_]/MFI_background_, where MFI_background_ represents the value without peptide. FI > 1.5 indicated a high affinity to HLA-A*0201 molecules, 1.0 < FI < 1.5 indicated a moderate affinity to the HLA-A*0201 molecule, and 0.5 < FI < 1.0 indicated that the peptide had low affinity to the HLA-A*0201 molecule. All samples were tested in triplicates.

### T cell proliferation assay

CD8^+^T cells were isolated from the PBMCs by flow cytometry and then labeled with carboxy-fluorescein diacetate, 12 succinimidyl ester (CFSE) at room temperature for 1 h. The rest of cells were treated with mitomycin C for 1 h to inhibit cell proliferation, then counted and seeded into a 96 well U-bottom plate at a density of 2 × 10^5^ cells per well, the anti-CD3/28 antibodies were pre-coated in the U-bottom plate, followed by addition of 4 μg (1 μg/μl) candidate peptides and rhIL-2. After incubation for 7 days, the cells were harvested and the proliferation rates of CD8^+^T cells were analyzed by flow cytometry through the signaling of CFSE. All samples were tested in triplicates.

### Intracellular cytokines detection for IFN-γ

PBMCs from patients were counted and seeded at a density of 3 × 10^5^ cells per hole in a 24-well plate. 6 μl (1 μg/μl) candidate peptides were then added into the culture and incubated for 48 hours. For intracellular cytokine detection, the protein transportation inhibitors (Cell stimulation cocktail (plus protein transport inhibitors), eBioscience, Affymetrix, USA) were added into the co-culture system for 4 to 6 h before staining of CD8^+^ T cells with PercpCy5.5-conjugated anti-CD8a mAb. Cells were then fixed, permeabilized and stained with FITC-conjugated anti- IFN-γ antibody for 1 h at 4 °C. The IFN-γ producing CD8^+^T cells were tested by flow cytometry. All samples were tested in triplicates.

### Statistical analysis

The data was analyzed by using GraphPad Prism 5.0. Software (GraphPad Software Inc, San Diego, CA, USA). Differences between two groups were subjected to non-parametric student’s t-test. *P* values less than 0.05 were considered statistically significant.

## Additional Information

**How to cite this article**: Cui, T. *et al*. Identification of Novel HLA-A*0201-Restricted CTL Epitopes in Chinese Vitiligo Patients. *Sci. Rep*. **6**, 36360; doi: 10.1038/srep36360 (2016).

**Publisher’s note:** Springer Nature remains neutral with regard to jurisdictional claims in published maps and institutional affiliations.

## Supplementary Material

Supplementary Information

## Figures and Tables

**Table 1:** Candidate epitopes identified by Elispot assay.

Proteins	Epitope peptide No.	Sequences (AA sites)*
Tyrosinase	P28	TLEGFASPL (343–351)
Tyrosinase	P41	AMVGAVLTA (482–490)
Gp100	P112	QILKGGSGT (556–564)
Gp100	P118	QLIMPGQEA (583–591)
Gp100	P119	IMPGQEAGL (585–593)

^*^AA sites: amino acid sites.

**Table 2:** HLA-A*0201 Vitiligo patient’s characteristics, vitiligo type, disease duration, autoimmunity and disease activity.

Patient no.	Sex/age	Vitiligo type	Duration of disease (month)	Other autoimmune disease	Disease activity
6	M/22	Generalized	24	No	progressive
10	F/18	Generalized	204	No	progressive
11	F/39	Generalized	36	No	progressive
19	F/44	Generalized	240	Yes	progressive
20	M/19	Generalized	36	No	progressive
21	M/42	Generalized	240	Yes	progressive
22	F/29	Generalized	1.5	No	progressive
26	M/6	Generalized	60	Yes	progressive
28	M/21	Generalized	240	No	progressive
30	F/22	Generalized	16	No	progressive
31	M/30	Generalized	84	No	progressive
33	M/19	Generalized	4	No	progressive
35	F/35	Generalized	180	No	progressive
42	F/22	Generalized	96	No	progressive
47	M/7	Generalized	204	No	progressive
48	M/24	Generalized	108	No	progressive
52	F/30	Generalized	15	No	progressive
53	M/36	Generalized	240	No	progressive
57	M/21	Generalized	60	No	progressive
58	M/30	Generalized	24	No	progressive
59	M/32	Generalized	18	No	progressive
64	M/34	Generalized	18	No	progressive
67	M/8	Generalized	84	No	progressive
68	M/25	Generalized	40	No	progressive
71	F/16	Generalized	1	No	progressive
75	F/59	Generalized	7	No	progressive
77	F/15	Generalized	6	No	progressive
84	M/46	Generalized	3	No	progressive
86	F/47	Generalized	36	No	progressive
90	M/26	Generalized	12	No	progressive
92	M/19	Generalized	9	No	progressive
95	M/54	Generalized	240	No	progressive
103	M/14	Generalized	300	No	progressive
104	M/41	Generalized	24	No	progressive
109	M/8	Generalized	84	No	progressive
112	F/22	Generalized	120	No	progressive
116	M/27	Generalized	24	No	progressive
118	M/41	Generalized	72	No	progressive
121	F/39	Generalized	4	No	progressive
122	F/20	Generalized	2	No	progressive
126	F/48	Generalized	324	No	progressive
127	M/18	Generalized	20	No	progressive
132	F/50	Generalized	72	Yes	progressive
133	M/22	Generalized	12	No	progressive
141	M/16	Generalized	72	Yes	progressive
146	M/25	Generalized	72	No	progressive
147	F/18	Generalized	84	No	progressive
150	M/16	Generalized	6	No	progressive
156	F/41	Generalized	36	No	progressive
165	M/19	Generalized	19	No	progressive
174	M/25	Generalized	48	No	progressive
177	M/37	Generalized	120	No	progressive
181	F/49	Generalized	36	No	progressive
184	F/36	Generalized	180	No	progressive
187	M/39	Generalized	12	No	progressive
190	F/35	Generalized	312	No	progressive
194	F/37	Generalized	252	No	progressive
202	M/29	Generalized	120	No	progressive
203	M/33	Generalized	180	No	progressive
209	F/40	Generalized	7	Yes	progressive
213	M/21	Generalized	84	No	progressive
215	M/14	Generalized	24	No	progressive
224	M/24	Generalized	2	No	progressive
225	M/35	Generalized	180	No	progressive
230	F/20	Generalized	264	No	progressive
232	M/46	Generalized	72	No	progressive
233	F/32	Generalized	120	No	progressive
239	M/8	Generalized	96	No	progressive
245	M/20	Generalized	324	No	progressive
247	M/35	Generalized	180	No	progressive
251	M/32	Generalized	65	No	progressive
252	F/49	Generalized	36	No	progressive
255	F/31	Generalized	168	No	progressive
260	M/32	Generalized	288	No	progressive
263	F/28	Generalized	180	No	progressive
264	F/56	Generalized	72	No	progressive
266	M/36	Generalized	180	No	progressive
