# Supplementary material for: Identification of Novel HLA-A*0201-Restricted CTL Epitopes in Chinese Vitiligo Patients
Source: Sci Rep. 2016 Nov 8;6:36360. doi: 10.1038/srep36360 (PMC5099573; doi:10.1038/srep36360)
Supplement: Supplementary Information [file srep36360-s1.pdf]

Identification of Novel HLA-A*0201-Restricted CTL Epitopes in Chinese Vitiligo Patients

Tingting Cui[1], Xiuli Yi[1], Sen Guo[1], Fubo Zhou[1], Ling Liu[1], Kai Li[1], Chunying Li[1], Tianwen Gao[1]*

[1]Department of Dermatology, Xijing Hospital, Fourth Military Medical University,

127 Changle West Road, Xi'an, shaanxi, 710032, China.

**Corresponding author: Tianwen Gao, MD, PhD, Department of Dermatology, Xijing Hospital,*

Fourth Military Medical University, Xi'an 710032, China.

Tel: +86 29 8477 5401; fax: +86 29 8477 5401; E-mail: gaotw@fmmu.edu.cn.

Table S1 Numbers and sequences of 51 predicted nonapeptides from tyrosinase

Numbers	AA sites	Sequences
P1	1-9	MLLAVLYCL
P 2	2-10	LLAVLYCLL
P 3	5-13	VLYCLLWSF
P 4	7-15	YCLLWSFQT
P 5	9-17	LLWSFQTSA
P 6	55-63	CQNILLSNA
P 7	57-65	NILLSNAPL
P 8	64-72	PLGPQFPFT
P 9	111-119	NCTERRLLV
P 10	130-138	EKDKFFAYL
P 11	133-141	KFFAYLTLA
P 12	137-145	YLTLAKHTI
P 13	171-179	NIYDLFVWM
P 14	193-201	EIWRDIDFA
P 15	200-208	FAHEAPAFL
P 16	207-215	FLPWHRLFL
P 17	208-216	LPWHRLFLL
P 18	212-220	RLFLLRWEQ
P 19	214-222	FLLRWEQEI
P 20	224-232	KLTGDENFT
P 21	254-262	GQHPTNPNL
P 22	261-269	NLLSPASFF
P 23	266-274	ASFFSSWQI
P 24	314-322	SSADVEFCL
P 25	316-324	ADVEFCLSL
P 26	323-331	SLTQYESGS
P 27	336-344	ANFSFRNTL
P 28	343-351	TLEGFASPL
P 29	358-366	SQSSMHNAL
P 30	365-373	ALHIYMNGT
P 31	380-388	SANDPIFLL
P 32	385-393	IFLLHHAFV
P 33	393-401	VDSIFEQWL

P 34	406-414	PLQEVYPEA
P 35	425-433	YMVPFIPLY
P 36	451-459	YLQDSDPDS
P 37	460-468	FQDYIKSYL
P 38	473-481	RIWSWLLGA
P 39	475-483	WSWLLGAAM
P 40	478-486	LLGAAMVGA
P 41	482-490	AMVGAVLTA
P 42	487-495	VLTALLAGL
P 43	490-498	ALLAGLVSL
P 44	491-499	LLAGLVSLL
P 45	506-514	QLPEEKQPL
P 46	444-452	DLGYDYSYL
P 150	483-491	MVGAVLTAL
P 151	225-233	LTGDENFTI
P 152	484-492	VGAVLTALL
P 153	169-177	DINIYDLFV
P 154	476-484	SWLLGAAMV

Table S2 Numbers and sequences of 106 predicted nonapeptides from gp100

Numbers	Amino acid sites	Sequences
P46-2	2-10	DLVLKRCLL
P47	4-12	VLKRCLLHL
P48	9-17	LLHLAVIGA
P49	13-21	AVIGALLAV
P50	18-26	LLAVGATKV
P51	25-33	KVPRNQDWL
P52	47-55	QLYPEWTEA
P53	50-58	PEWTEAQRL
P54	69-77	KVSNDGPTL
P55	88-96	LNFPGSQKV
P56	95-103	KVLPDGQVI
P57	97-105	LPDGQVIWV

P58	100-108	GQVIWVNNT
P59	102-110	VIWVNNTII
P60	113-121	SQVWGGQPV
P61	144-152	SQKRSFVYV
P62	147-155	RSFVYVWKT
P63	154-162	KTWGQYWQV
P64	162-170	VLGGPVSGL
P65	171-179	SIGTGRAML
P66	178-186	MLGTHTMEV
P67	180-188	GTHTMEVTV
P68	205-213	SAFTITDQV
P69	209-217	ITDQVPFSV
P70	211-219	DQVPFSVSV
P71	230-238	KHFLRNQPL
P72	236-244	QPLTFALQL
P73	243-251	QLHDPSGYL
P74	248-256	SGYLAEADL
P75	250-258	YLAEADLSY
P76	267-275	GTLISRALV
P77	268-276	TLISRALVV
P78	269-277	LISRALVVT
P79	273-281	ALVVTHTYL
P80	280-288	YLEPGPVTA
P81	286-294	VTAQVVLQA
P82	288-296	AQVVLQAAI
P83	290-298	VVLQAAIPL
P84	291-299	VLQAAIPLT
P85	298-306	LTSCGSSPV
P86	325-333	GQVPTTEVV
P87	343-351	AEPSGTTSV
P88	350-358	SVQVPTTEV
P89	356-364	TEVISTAPV
P90	358-366	VISTAPVQM
P91	364-372	VQMPTAEST
P92	373-381	GMTPEKVPV

P93	384-392	VMGTTLAEM
P94	400-408	MTPAEVSIV
P95	408-416	VVLSGTTAA
P96	416-424	AQVTTTEWV
P97	443-451	SIMSTESIT
P98	449-457	SITGSLGP
P99	453-461	SLGPLLDGT
P100	456-464	PLLDGTATL
P101	463-471	TLRLVKRQV
P102	465-473	RLVKRQVPL
P103	469-477	RQVPLDCVL
P104	472-480	PLDCVLYRY
P105	476-484	VLYRYGSFS
P106	479-487	RYGSFSVTL
P107	485-493	VTLDIVQGI
P108	490-498	VQGIESAEI
P109	506-514	EGDAFELTV
P110	544-552	VLPSPACQL
P111	550-558	CQLVLHQIL
P112	556-564	QILKGGSGT
P113	563-571	GTYCLNVSL
P114	566-574	CLNVSLADT
P115	570-578	SLADTNSLA
P116	576-584	SLAVVSTQL
P117	578-586	AVVSTQLIM
P118	583-591	QLIMPGQEA
P119	585-593	IMPGQEAGL
P120	592-600	GLGQVPLIV
P121	594-603	GQVPLIVGI
P122	597-605	PLIVGILLV
P123	599-607	IVGILLVLM
P124	601-609	GILLVLMAV
P125	602-610	ILLVLMAVV
P126	603-611	LLVLMAVVL

P127	604-612	LVLMAVVLA
P128	605-613	VLMAVVLAS
P129	606-614	LMAVVLASL
P130	613-621	SLIYRRRLM
P131	619-627	RLMKQDFSV
P132	622-630	KQDFSVPQL
P133	630-638	LPHSSSHWL
P134	637-645	WLRLPRIFC
P135	639-647	RLPRIFCSC
P136	653-661	SPLLSGQQV
P155	11-19	HLAVIGALL
P156	17-25	KVSNDGPTL
P157	10-18	LHLAVIGAL
P158	169-177	GLSIGTGRA
P159	598-606	LIVGILLVL
P160	399-407	GMTPAEVSI
P161	591-599	AGLGQVPLI
P162	371-379	STGMTPEKV
P163	409-417	VLSGTTAAQ
P164	450-458	ITGSLGPLL
P165	569-577	VSLADTNSL
P166	571-579	LADTNSLAV
P167	595-603	Q**V**PLIVGI**L**
P168	397-405	ATGMTPAEV
P169	648-656	PIGENSPLL

Table S3 Numbers and sequences of 13 predicted nonapeptides from MelanA/Mart-1

Numbers	Amino acid sites	Sequences
P137	27-35	AAGIGILTV
P138	29-37	GIGILTVIL
P139	31-39	GILTVILGV
P140	32-40	ILTVILGVL
P141	34-42	TVILGVLLL

P142	35-43	VILGVLLLI
P143	39-47	VLLLIGCWY
P144	40-48	LLLIGCWYC
P145	56-64	ALMDKSLHV
P146	61-69	SLHVGTQCA
P147	95-103	PVVPNAPPA
P148	99-107	NAPPAYEKL
P149	41-49	LLIGCWYCR

Table S4 Numbers of peptide pools and peptides included in each pools

Numbers	Peptides included
M1	P1-P10
M2	P11-P20
M3	P21-P30
M4	P31-P40
M5	P41-P45、P46-1、P150-P154
M6	P46-2、P47-P55
M7	P56-P65
M8	P66-P75
M9	P76-P85
M10	P86-P95
M11	P96-P105
M12	P106-P115
M13	P116-P125
M14	P126-P135
M15	P136、P155-P163
M16	P164-P169
M17	P137-P146
M18	P147-P149

Data S1 Epitope prediction

The online software BIMAS (http://www-bimas.cit.nih.gov/molbio/hla_bind/) and SYFPEITHI (http://www.syfpeithi.de/bin/MHCServer.dll/EpitopePrediction.htm) were applied to predict the possible HLA-A*0201-restricted antigenic epitopes originated from tyrosinase, gp100, and MART-1. There were four steps to predict the peptides by the both online softwares. Firstly, we shoud select HLA-A* 02:01as MHC type; secondly, we chose nonamers (9 amino acid); thirdly, we enterd an amino acid sequence to analyze; finally, we submitted the above information and made the software run, then chose the peptides with high scores as peptides used in the elispot assay. Amino acids sequences are as follows:

tyrosinase (529 amino acids)

1 mllavlycll wsfqtsaghf pracvssknl mekeccppws gdrspcgqls grgscqnill

61 snaplgpqfp ftgvdd resw psvfynrtcq csgnfmgfnc gnckfgfwgp ncterrllvr

121 rnifdlsape kdkffayltl akhtissdyv ipigtygqmk ngstpmfndi niydlfvwmh

181 yyvsmdallg gseiwrdidf aheapaflpw hrlfllrweq eiqkltgden ftipywdwrd

241 aekcdictde ymggqhptnp nllspasffs swqivcsrle eynshqslcn gtpegplrrn

301 pgnhdksrtp rlpssadvef clsltqyesg smdkaanfsf rntlegfasp ltgiadasqs

361 smhnalhiym ngtmsqvqgs andpifllhh afvdsifeqw lrrhrplqev ypeanapigh

421 nresymvpfi plyrngdffi sskdlgydys ylqdsdpdsf qdyiksyleq asriwswllg

481 aamvgavlta llaglvsllc rhkrkqlpee kqpllmeked yhslyqshl

gp100 (661 amino acids)

1 mdlvlkrcll hlavigalla vgatkvprnq dwlgvsrqlr tkawnrqlyp ewteaqrldc

61 wrggqvslkv sndgptliga nasfsialnf pgsqkvlpdg qviwvnntii ngsqvwggqp

121 vypqetddac ifpdggpcps gswsqkrsfv yvwktwgqyw qvlggpvsgl sigtgramlg

181 ththmevtvyh rrgsrsyvpl ahsssaftit dqvpfsvsvs qlraldggnk hflrnqpltf

241 alqlhdpsgy laeadlsytw dfgdssgtli sralvvthty lepgpvtaqv vlqaaiplts

301 cgsspvpgtt dghrptaeap nttagqvptt evvgttpgqa ptaepsgtts vqvpttevis

361 tapvqmptae stgmtpekvp vsevmgttla emstpeatgm tpaevsivvl sgttaaqvtt

421 tewvettare lpipepegpd assimstesi tgslgplldg tatlrlvkrq vpldcvlyry

481 gsfsvtldiv qgiesaeilq avpsgegdaf eltvscqggl pkeacmeiss pgcqppaqrl

541 cqpvlpspac qlvlhqilkg gsgtyclnvs ladtnslavv stqlimpgqe aglgqvpliv

601 gillvlmavv lasliyrrrl mkqdfsvpql phssshwlrl prifcscpig enspllsgqq

661 v

MART-1 (118 amino acids)

1 mpredahfiy gypkkghghs yttaeeaagi giltvilgvl lligcwycrr rngyralmdk

61 slhvgtqcal trrcpqegfd hrdskvslqe kncepvvpna ppayeklsae qspppysp

Data S2 Reverse phase-high performance liquid chromatography results for peptides

Tyrosinase (51 peptides, P1~P46, P150~P154)

P1:

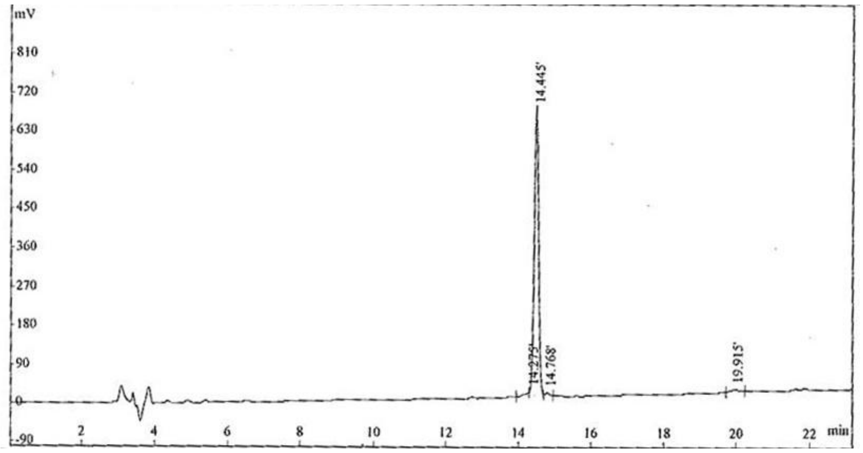

P2:

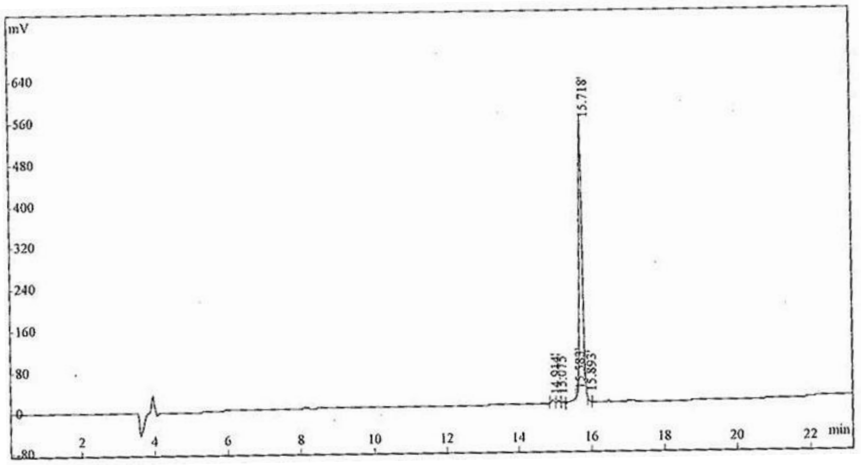

P3:

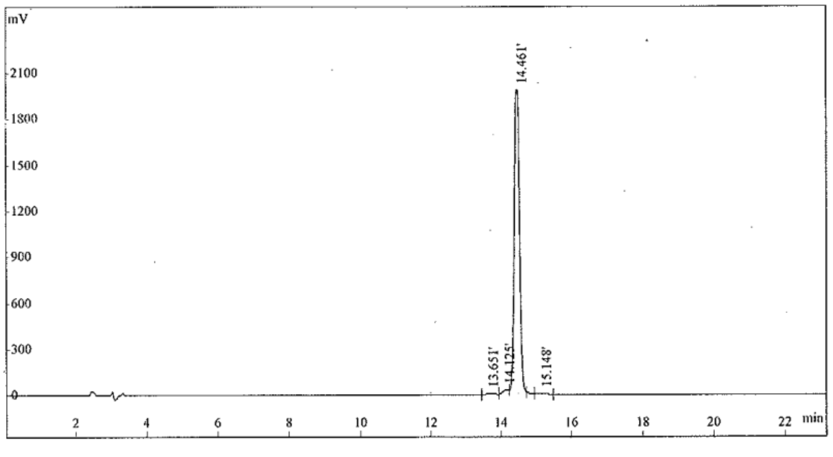

P4:

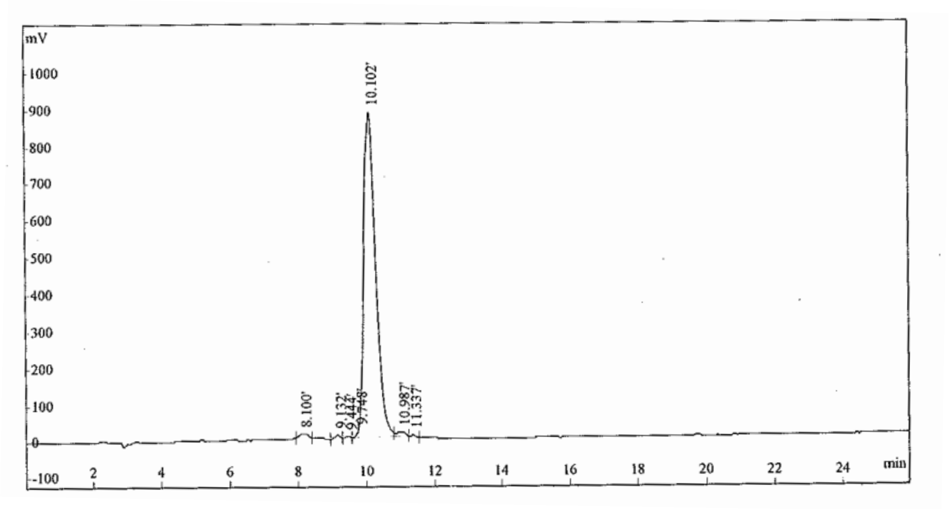
mV
1000
900
800
700
600
500
400
300
200
100
0
-100
10.102'
8.100'
9.132'
9.444'
9.748'
10.987'
11.337'
2
4
6
8
10
12
14
16
18
20
22
24
min

P5:

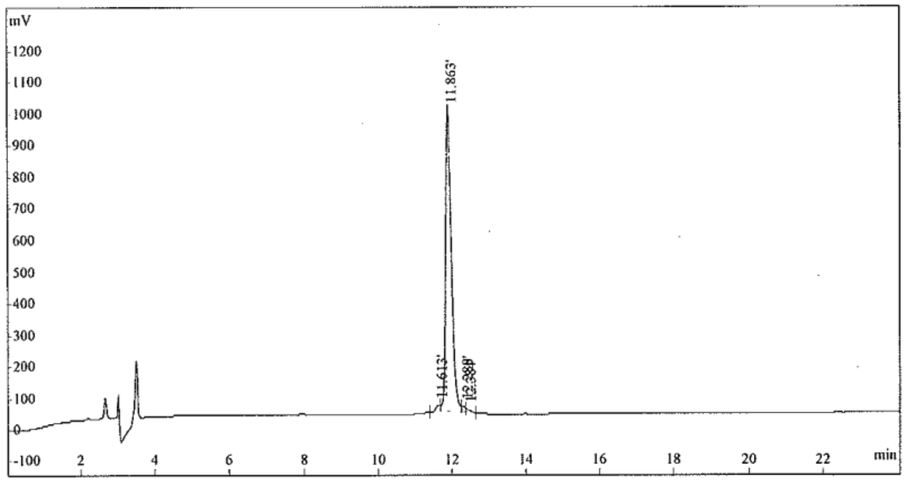
mV
1200
1100
1000
900
800
700
600
500
400
300
200
100
0
-100
11.863'
11.613'
2
4
6
8
10
12
14
16
18
20
22
min

P6:

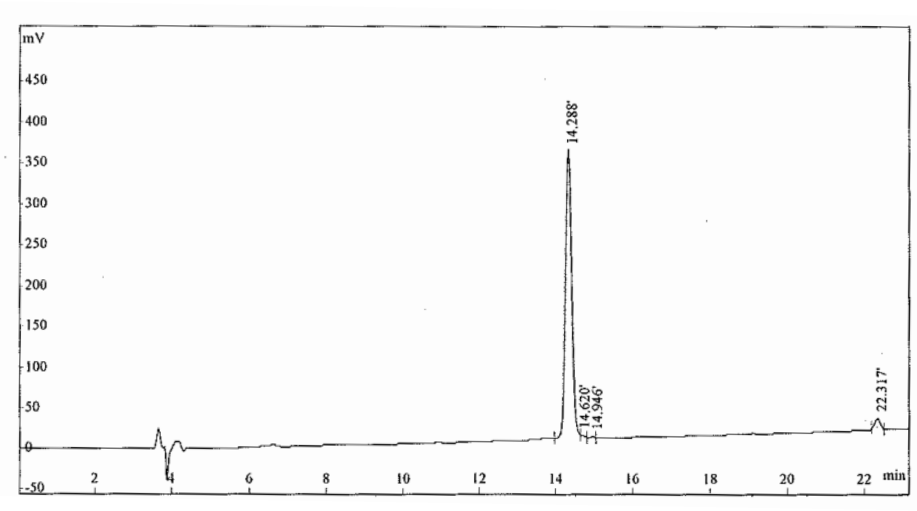
mV
450
400
350
300
250
200
150
100
50
0
-50
14.288'
14.620'
14.946'
22.317'
2
4
6
8
10
12
14
16
18
20
22
min

P7:

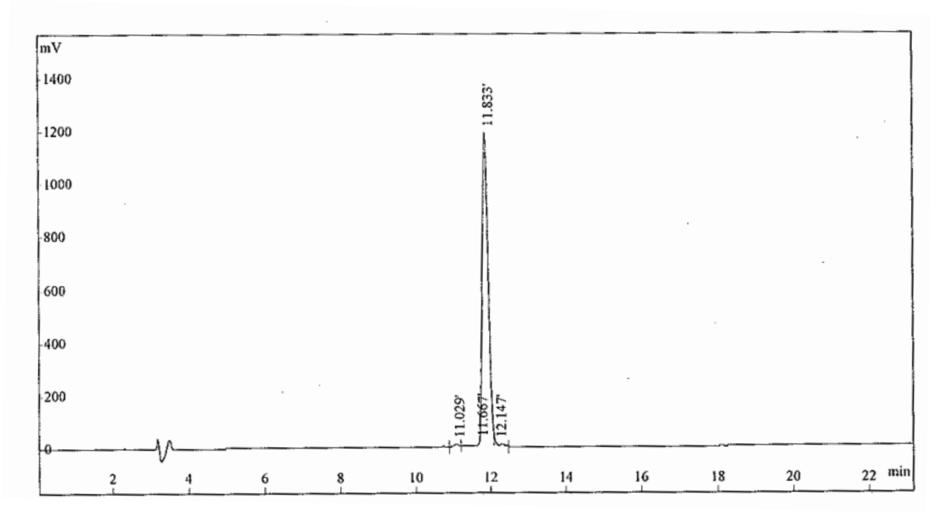
mV
1400
1200
1000
800
600
400
200
0
11.833'
11.029'
11.667'
12.147'
2
4
6
8
10
12
14
16
18
20
22
min

P8:

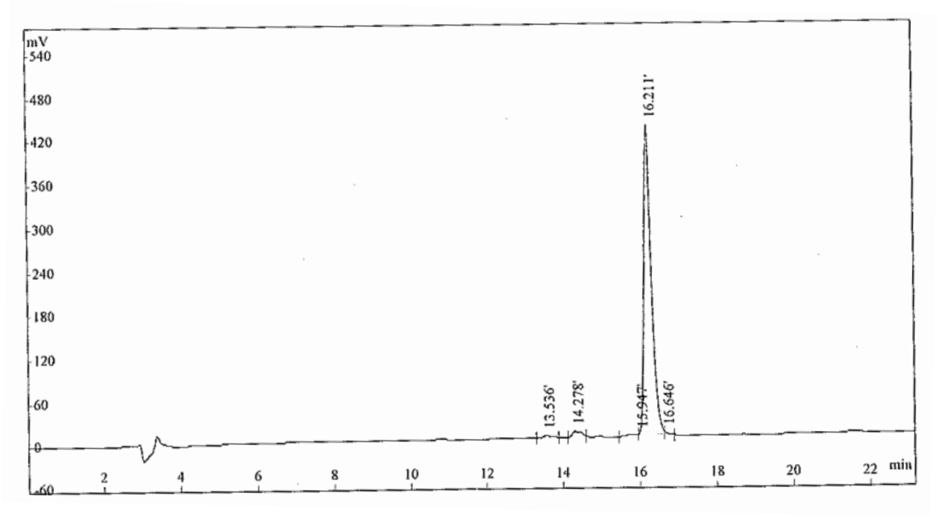
mV
540
480
420
360
300
240
180
120
60
0
-60
16.211'
13.536'
14.278'
15.947'
16.646'
2
4
6
8
10
12
14
16
18
20
22
min

P9:

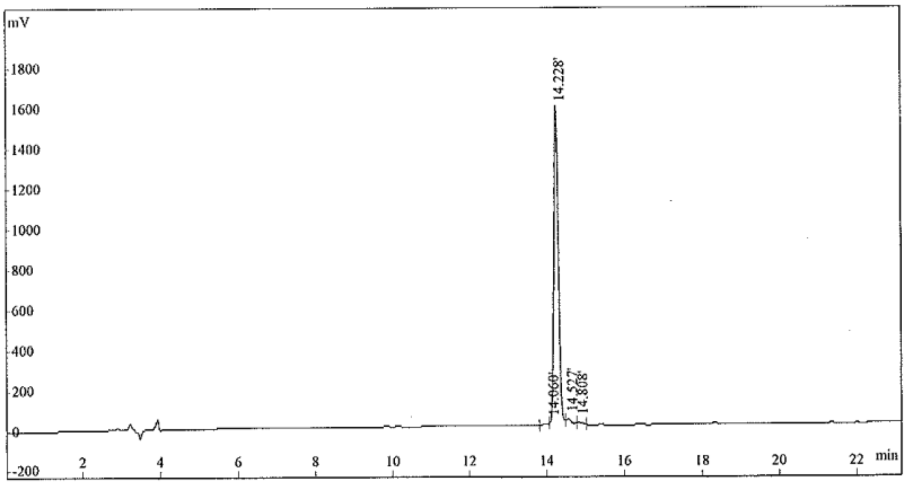
mV
1800
1600
1400
1200
1000
800
600
400
200
0
-200
14.228'
14.060'
14.527'
14.808'
2
4
6
8
10
12
14
16
18
20
22
min

P10:

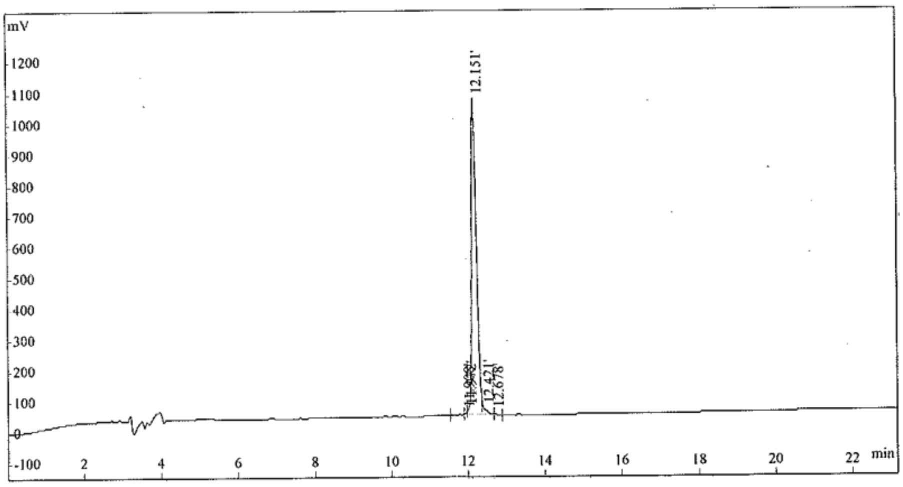

P11:

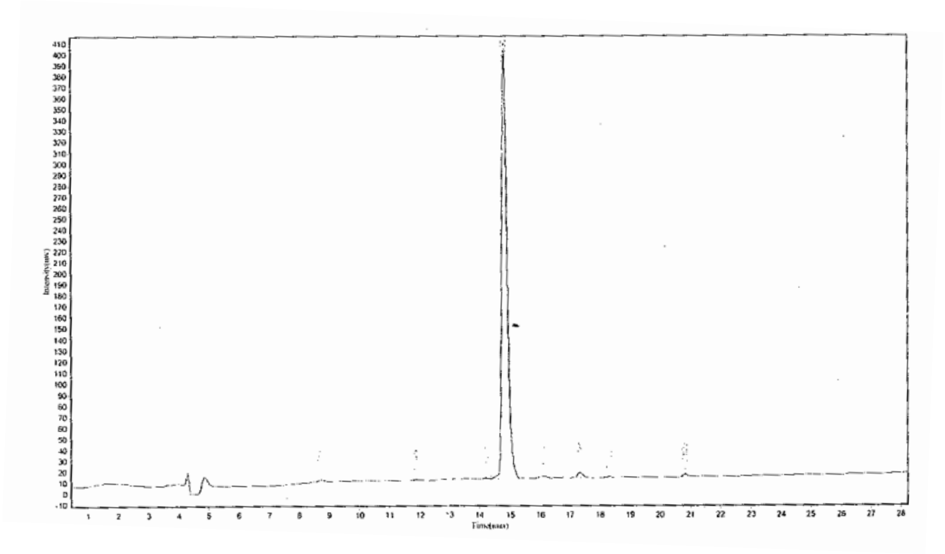

P12:

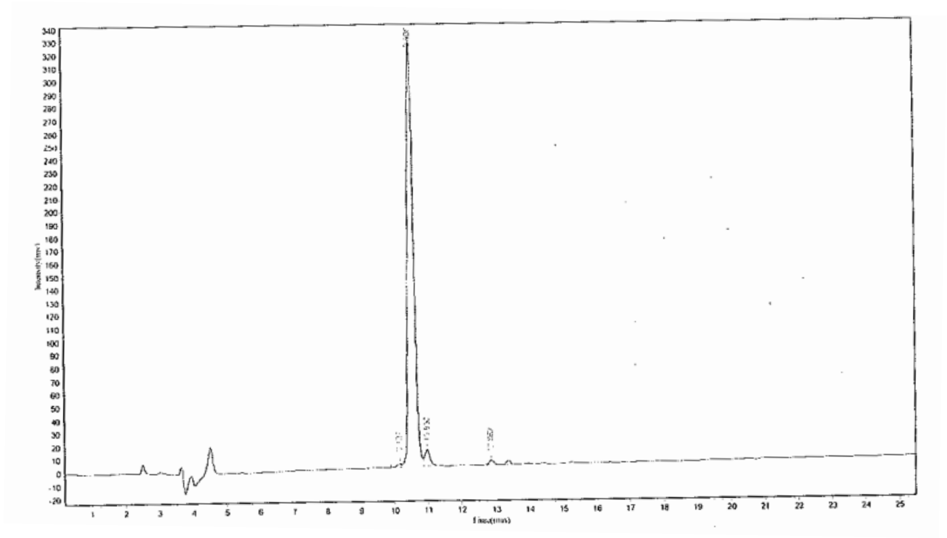

P13:

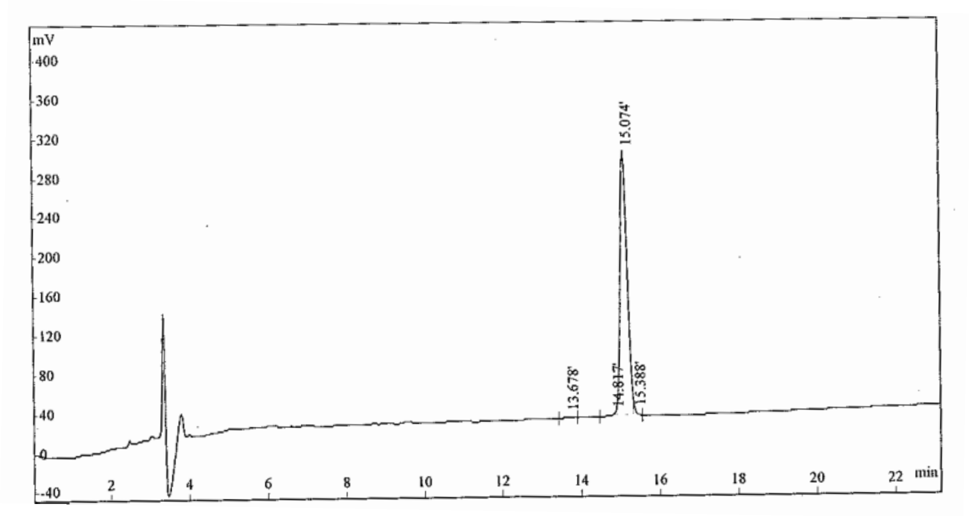
mV
400
360
320
280
240
200
160
120
80
40
0
-40
13.678'
14.817'
15.074'
15.388'
2
4
6
8
10
12
14
16
18
20
22
min

P14:

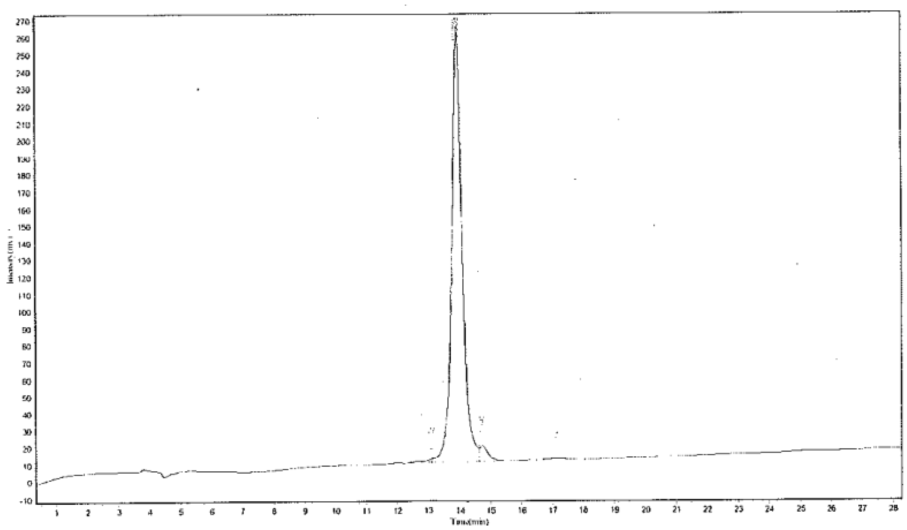

P15:

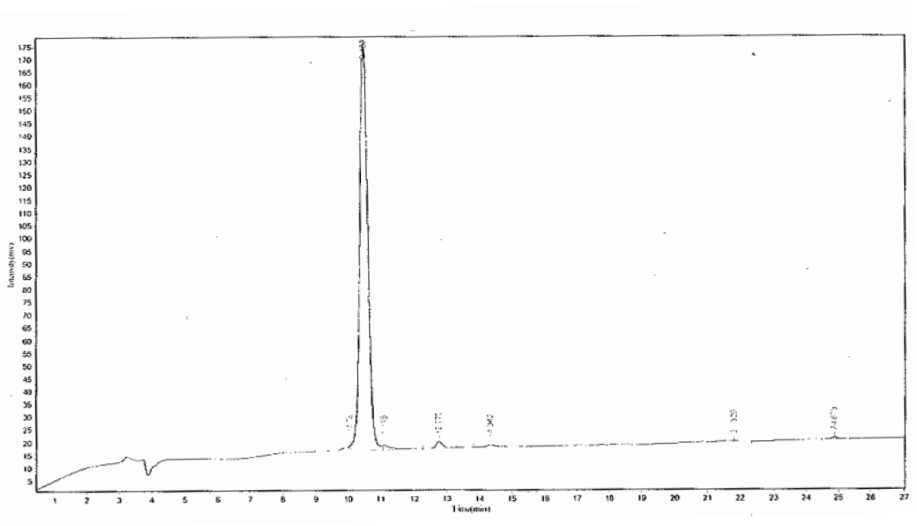

P16:

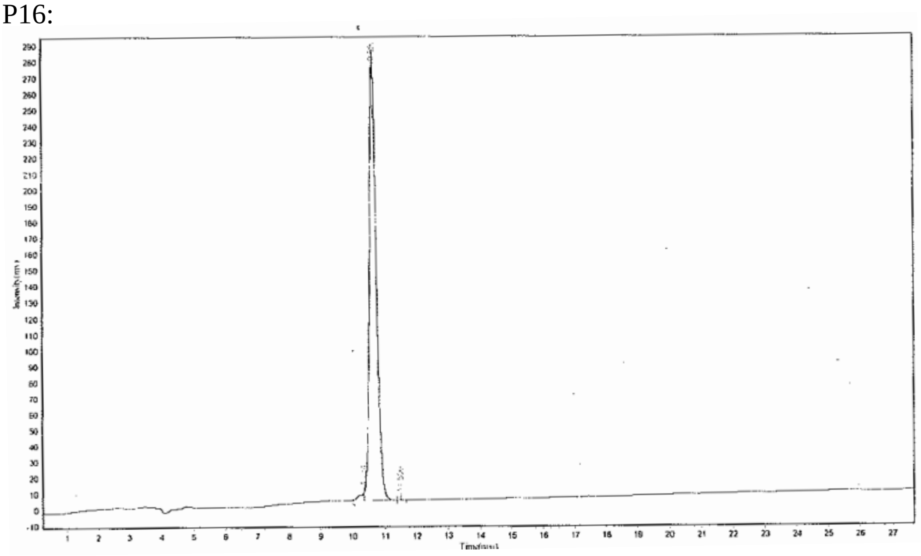

P17:

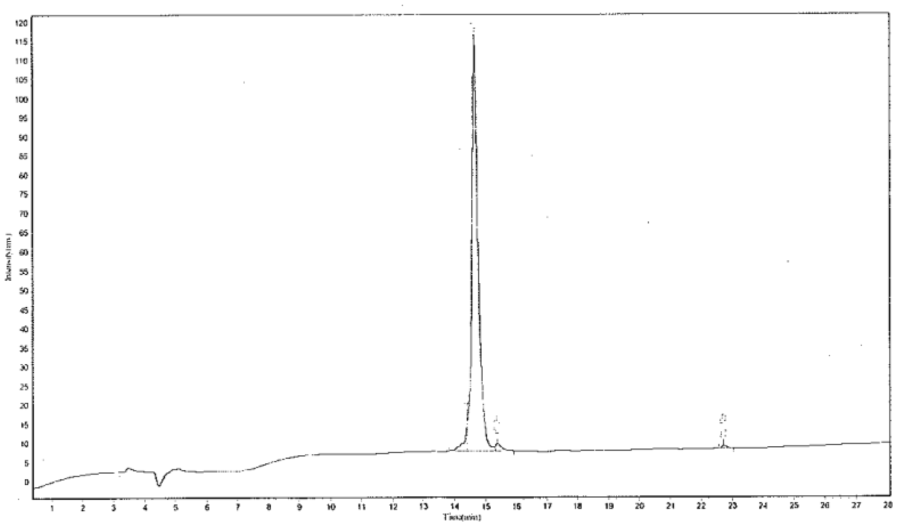

P18:

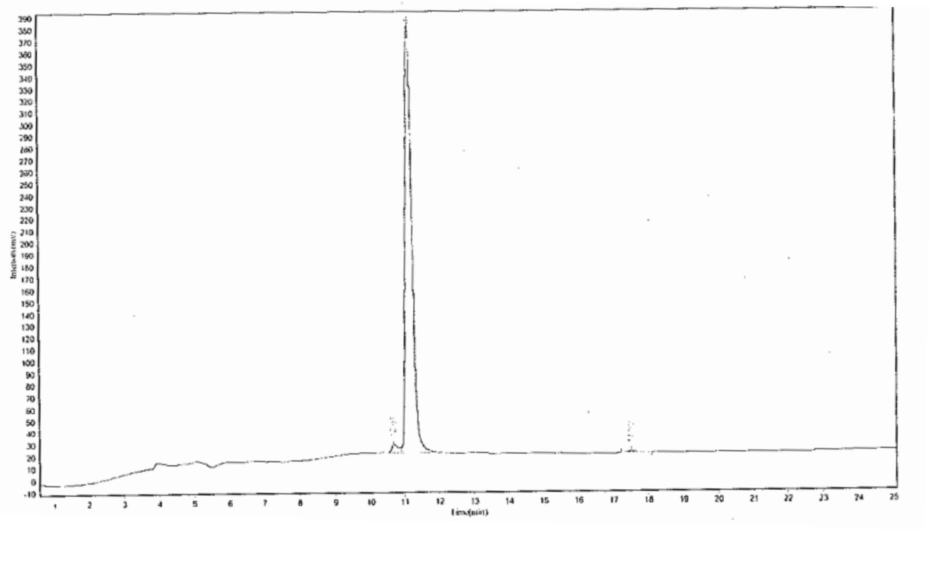

P19:

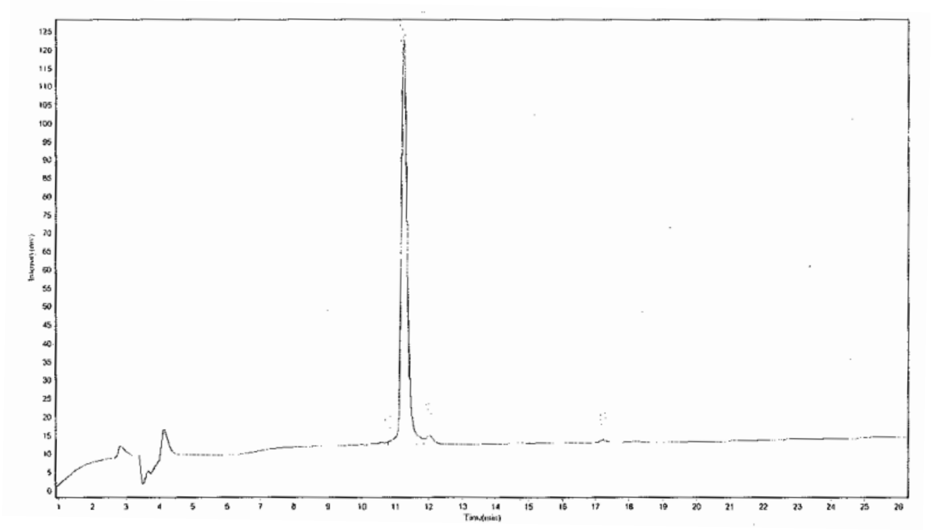

P20:

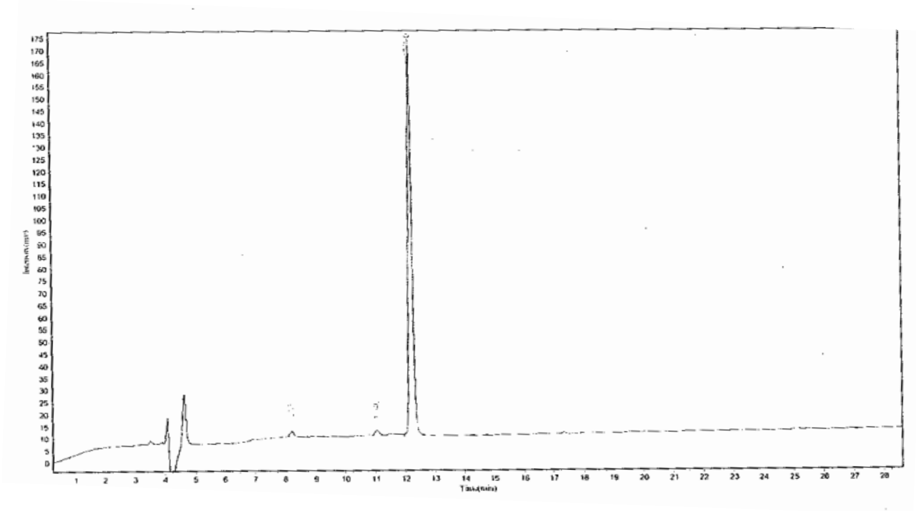

P21:

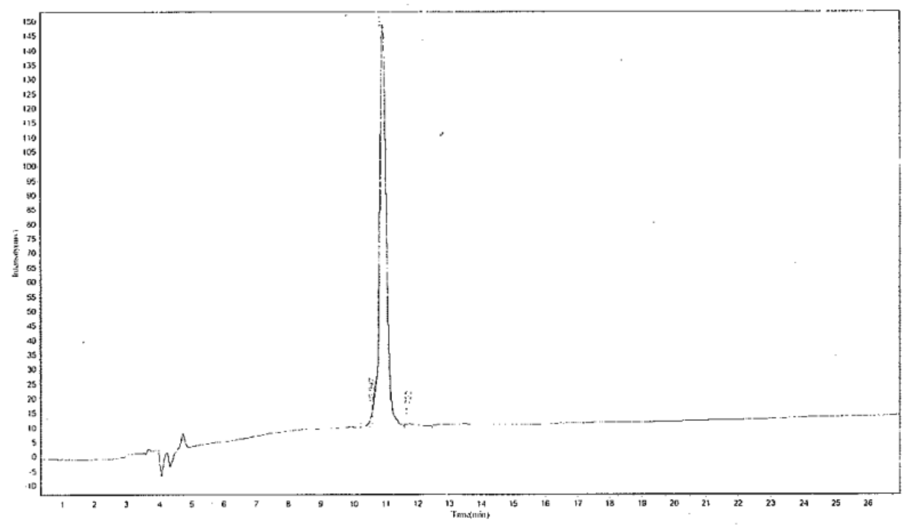

P22:

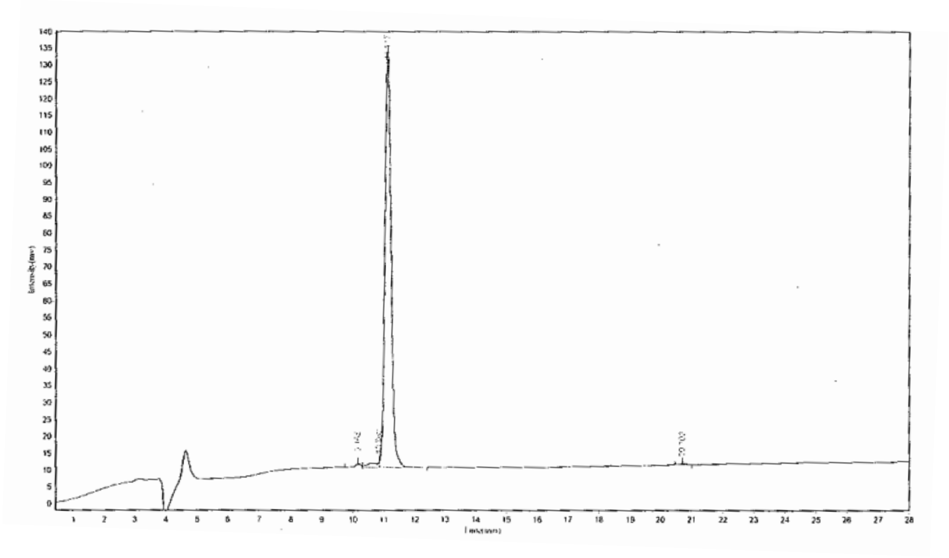

P23:

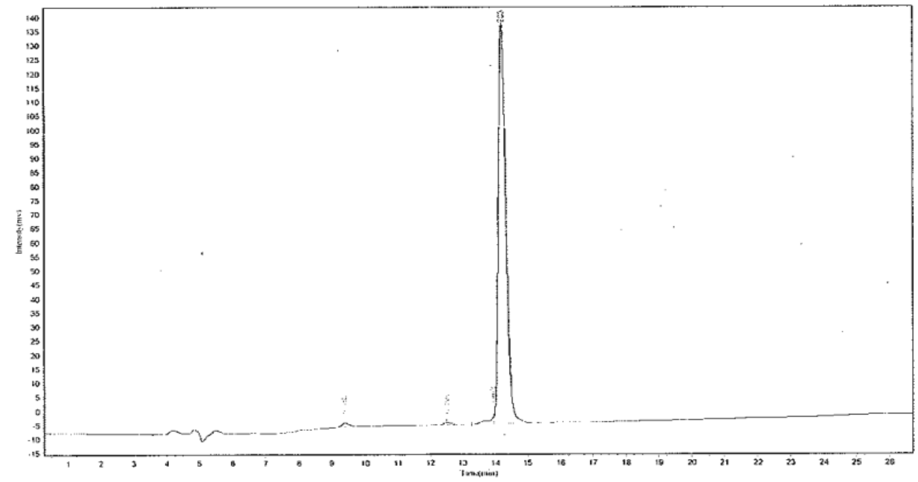

P24:

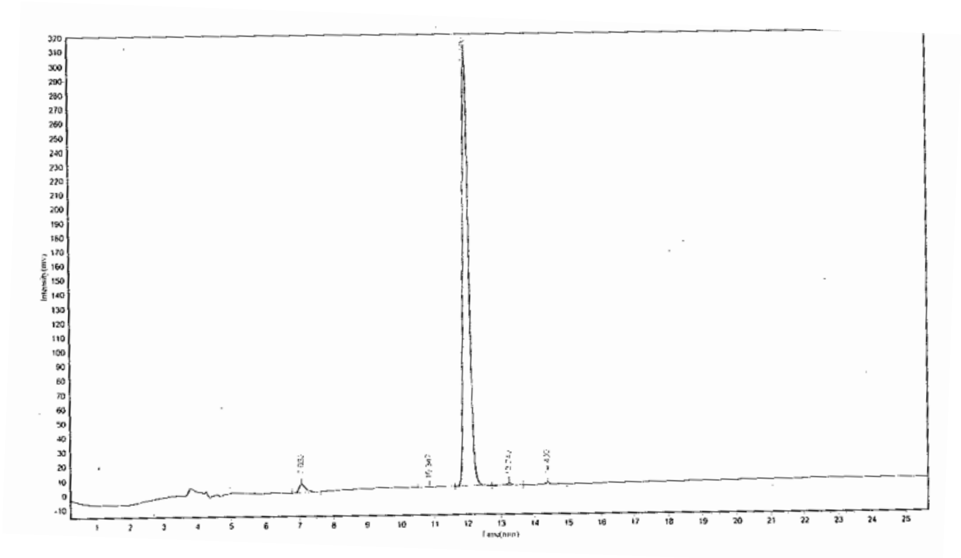

P25:

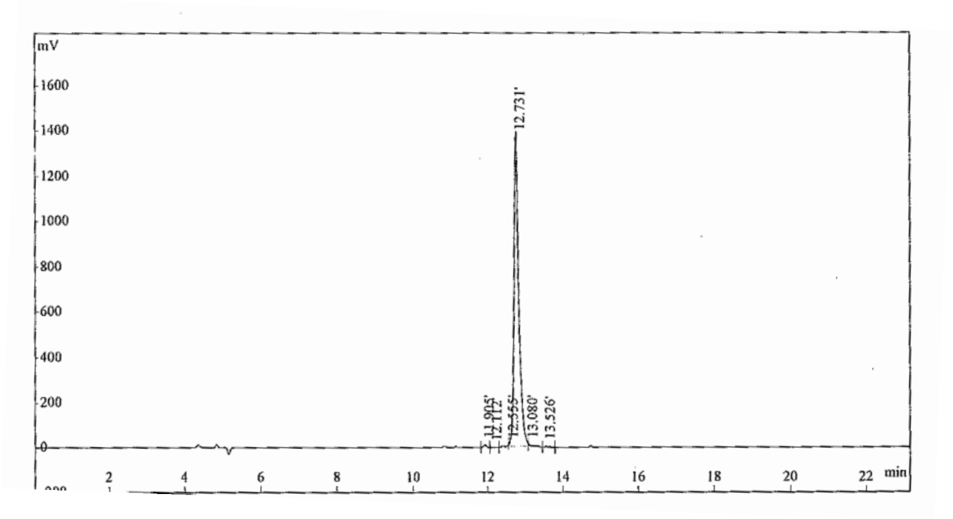
mV
1600
1400
1200
1000
800
600
400
200
0
12.731'
11.905'
12.112'
12.555'
13.080'
13.526'
2
4
6
8
10
12
14
16
18
20
22
min

P26:

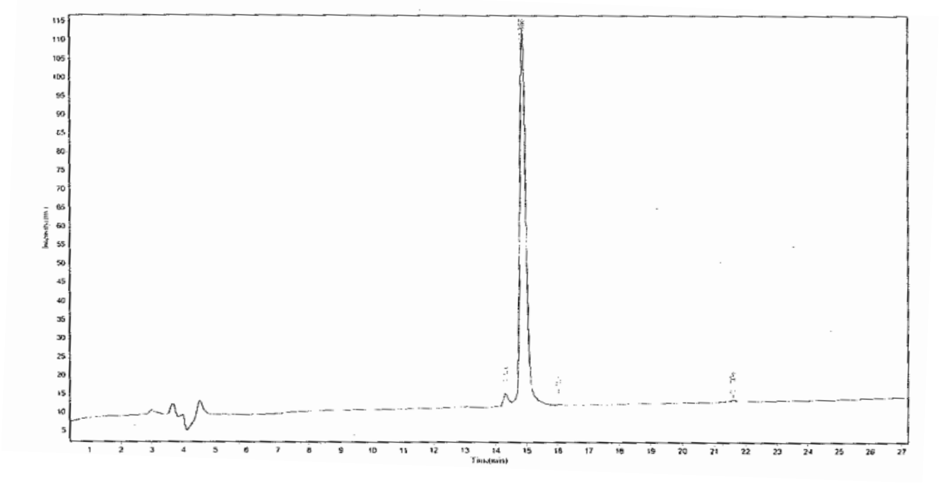

P27:

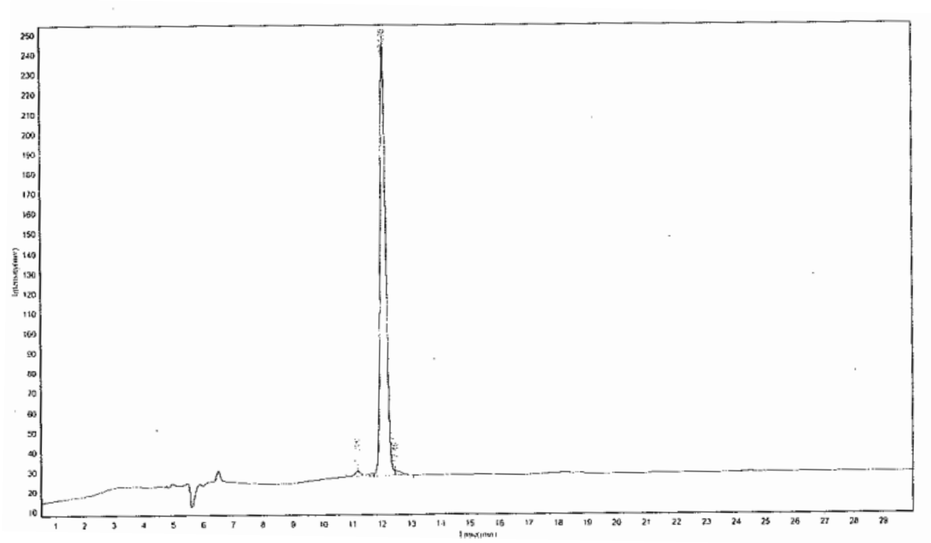

P28:

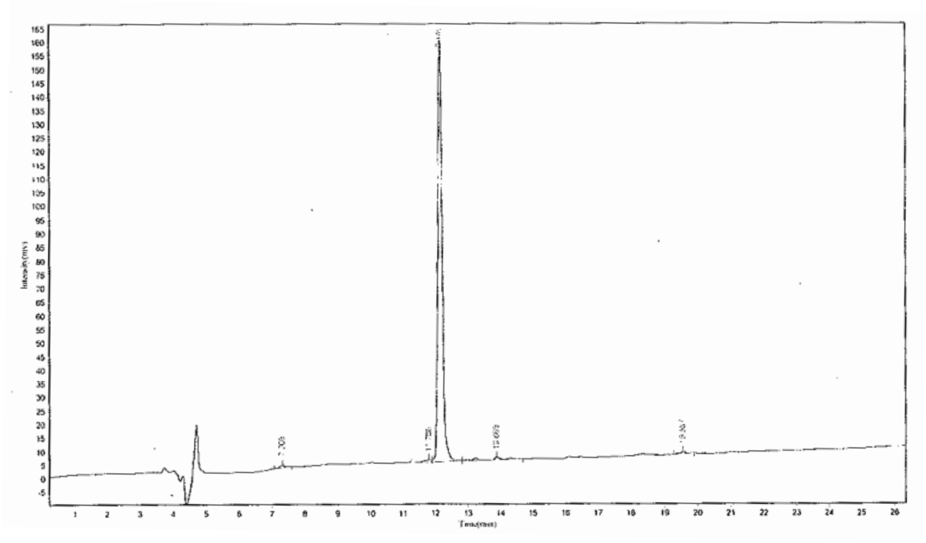

P29:

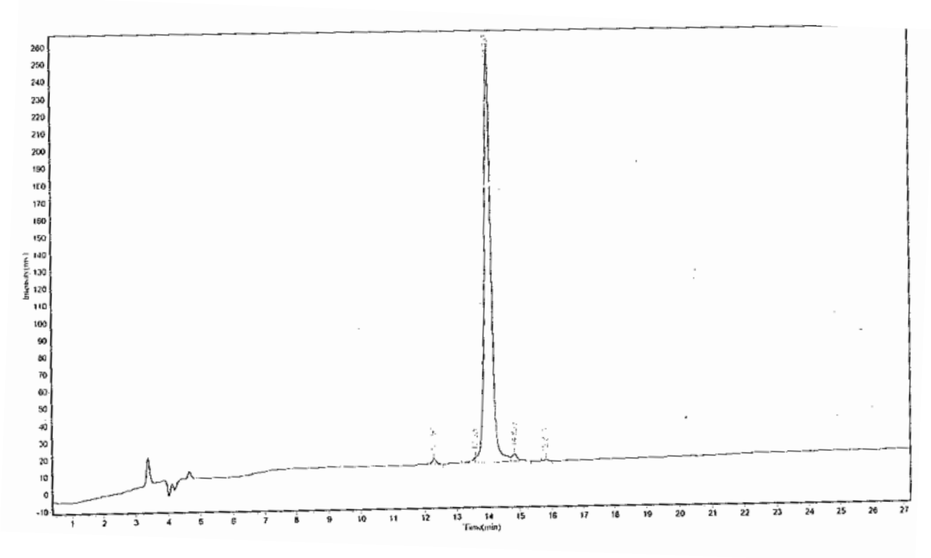

P30:

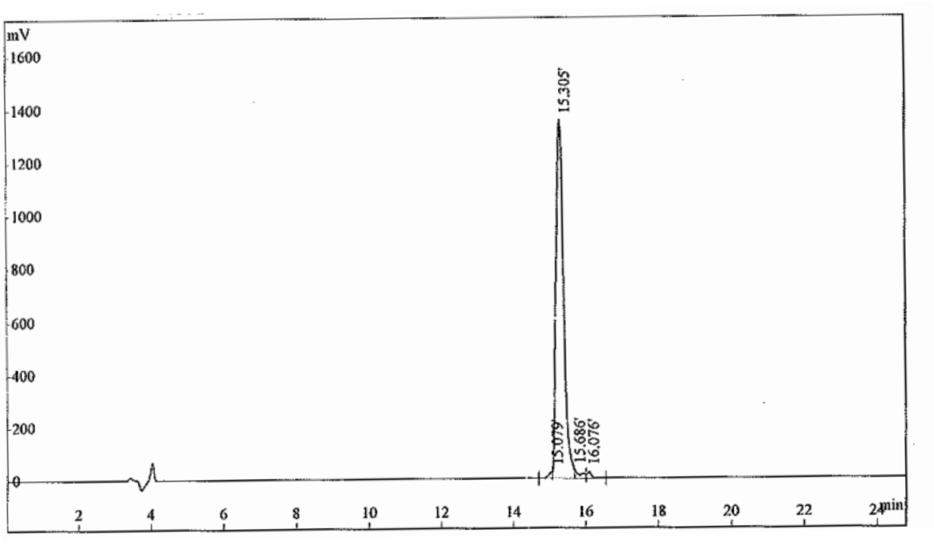
mV
1600
1400
1200
1000
800
600
400
200
0
15.305'
15.079'
15.686'
16.076'
2
4
6
8
10
12
14
16
18
20
22
24
min

P31:

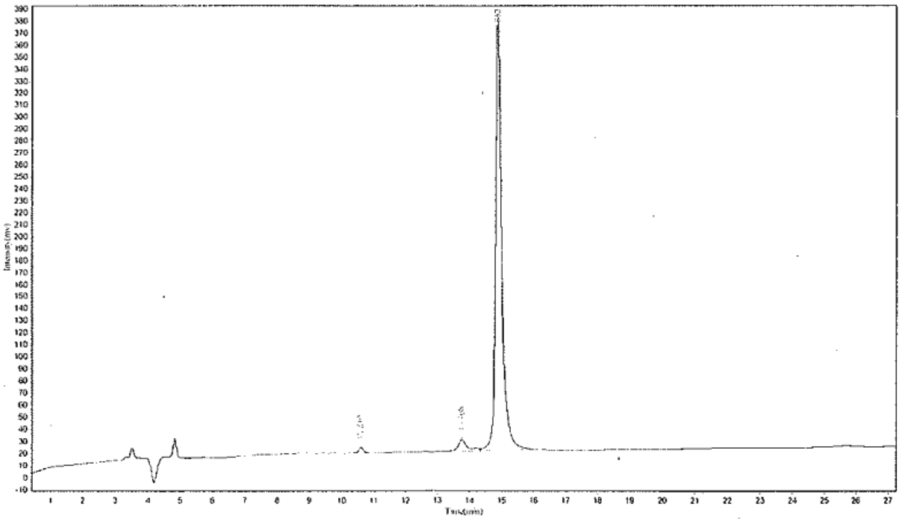

P32:

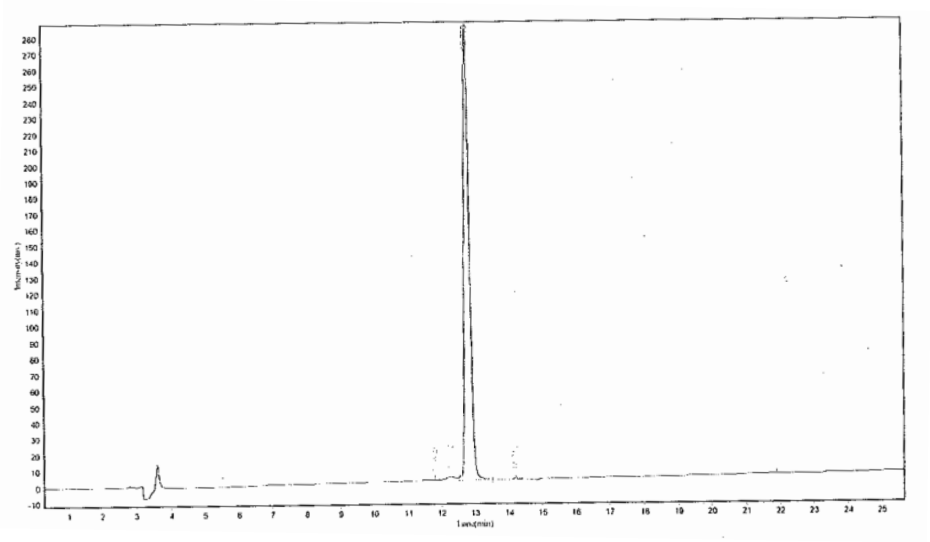

P33:

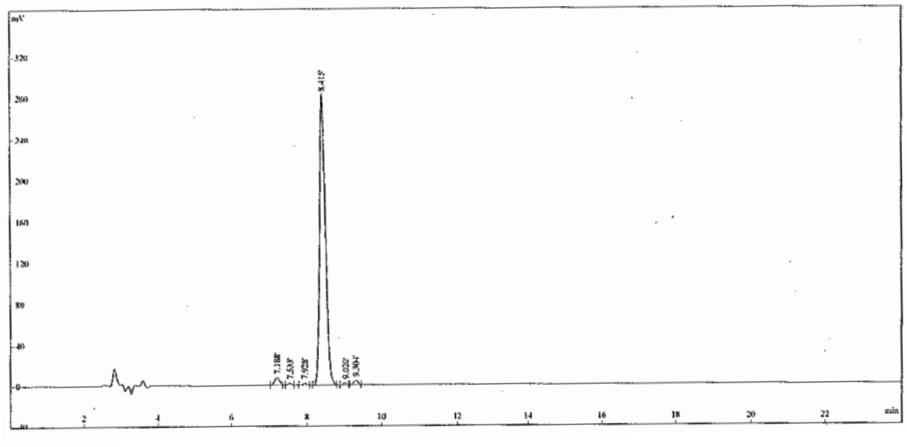

P34:

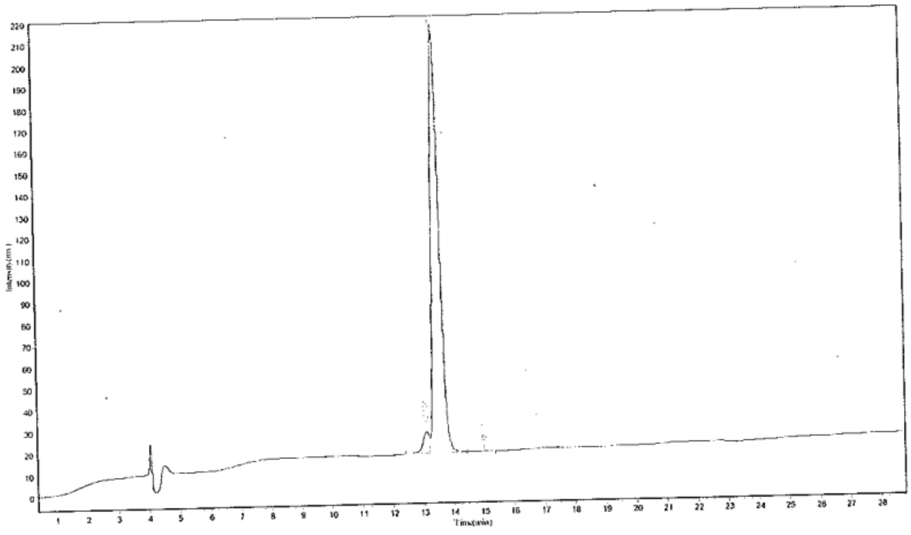

P35:

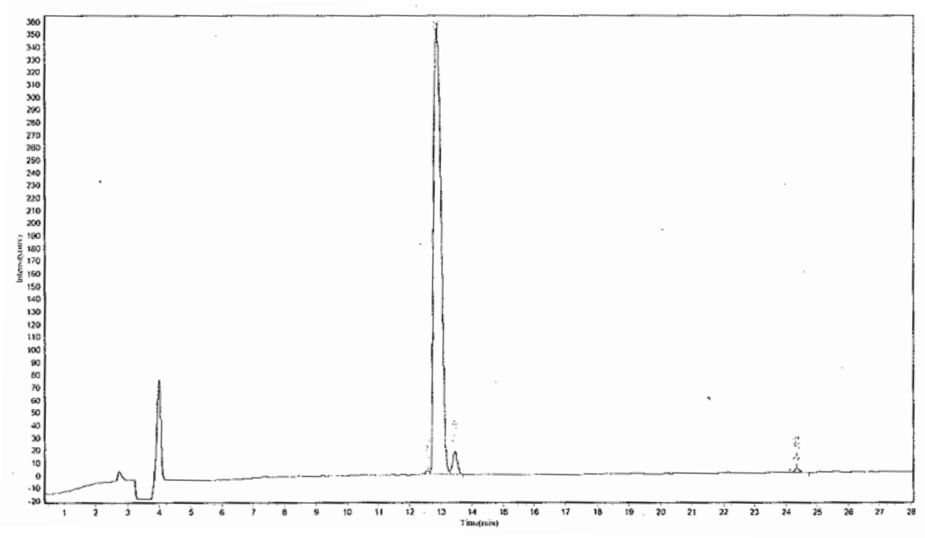

P36:

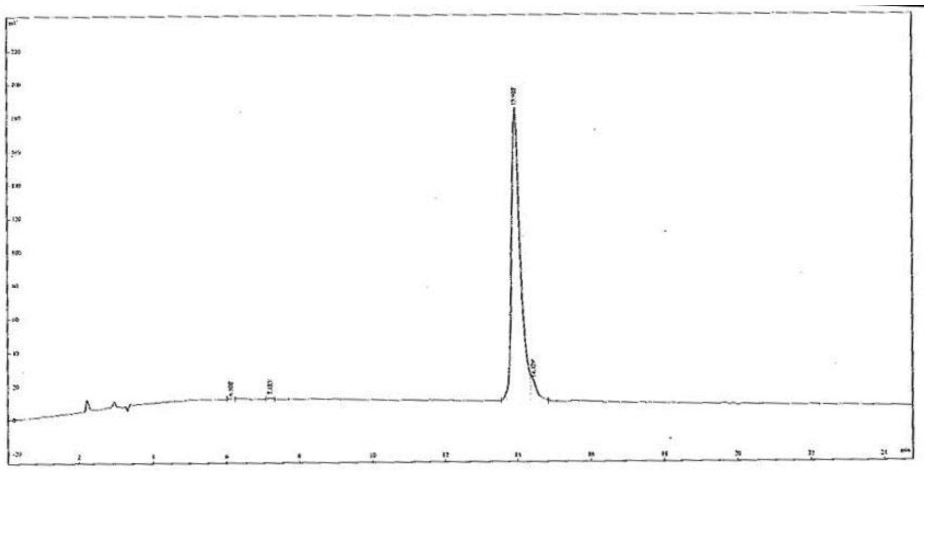

P37:

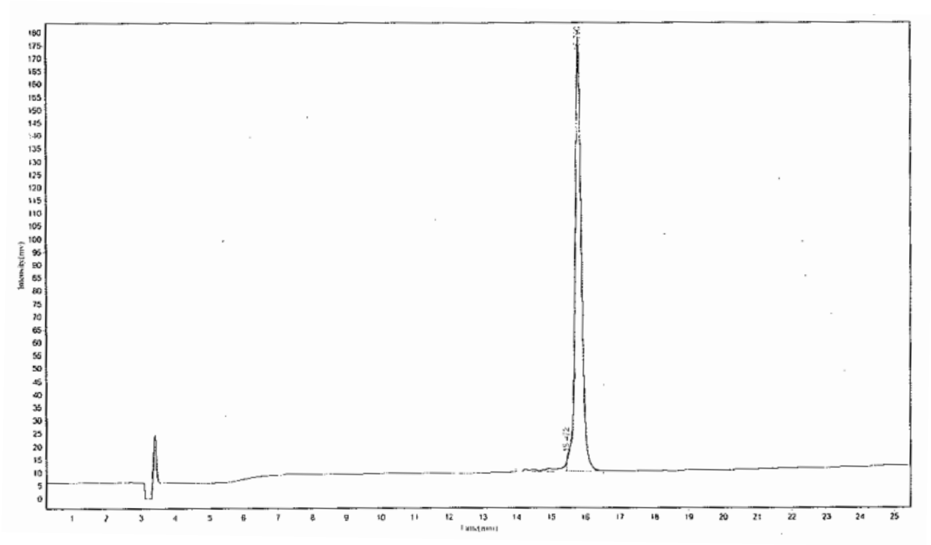

P38:

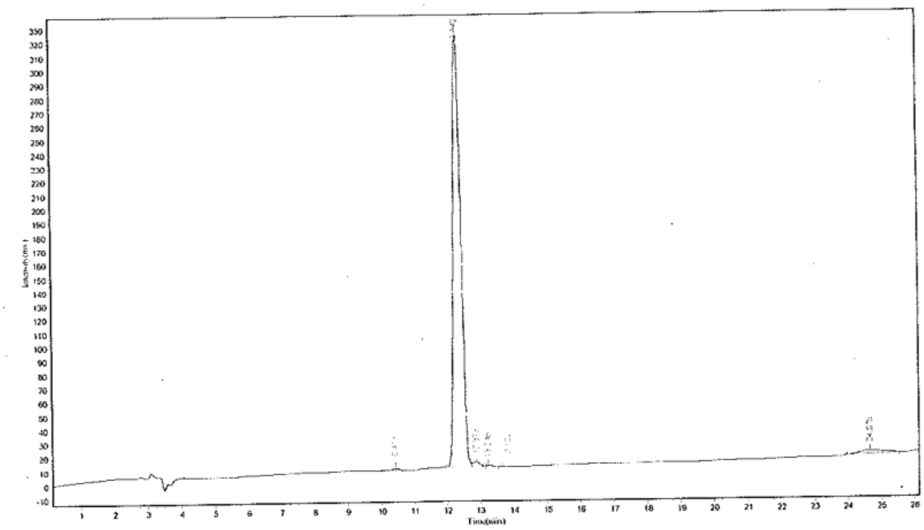

P39:

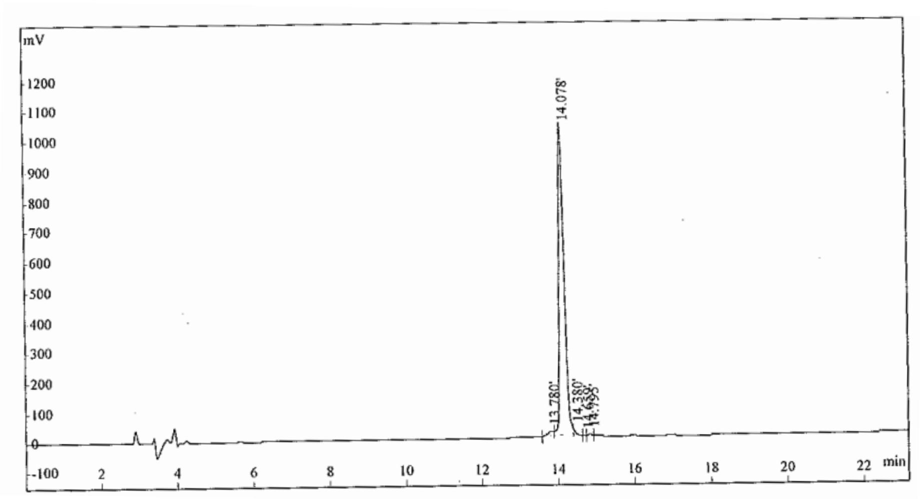
mV
1200
1100
1000
900
800
700
600
500
400
300
200
100
0
-100
14.078
13.780
14.380
2
4
6
8
10
12
14
16
18
20
22
min

P40:

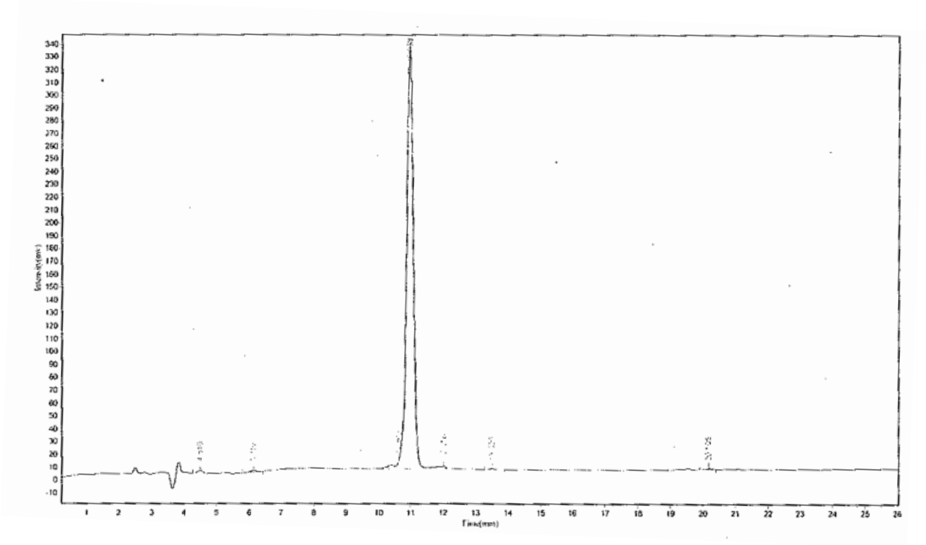

P41:

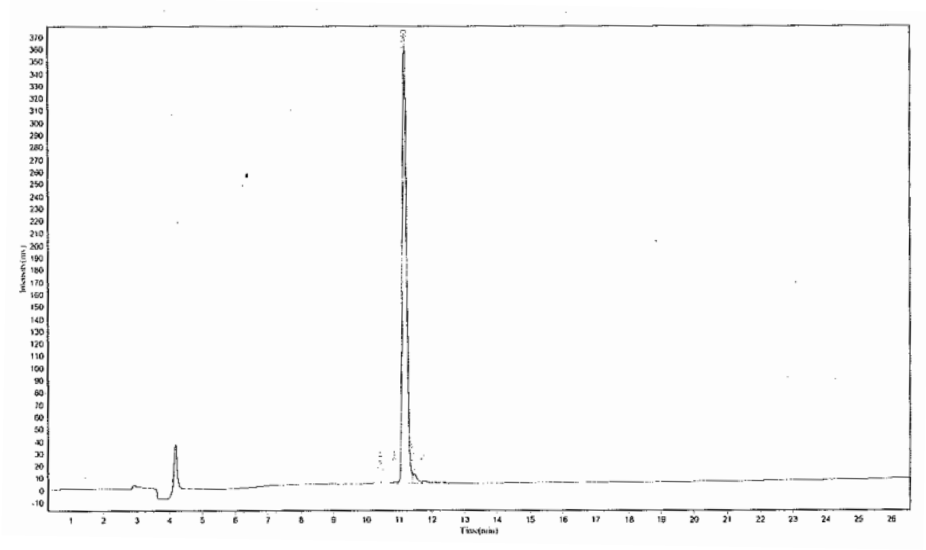

P42:

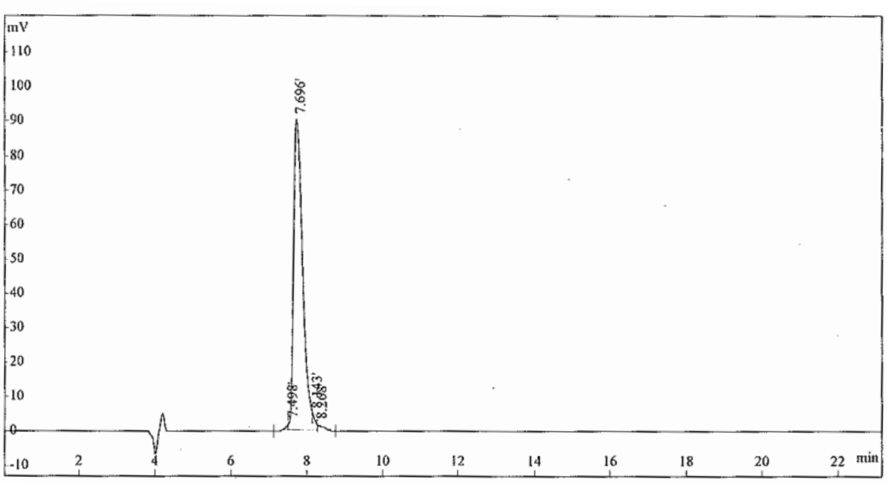

P43:

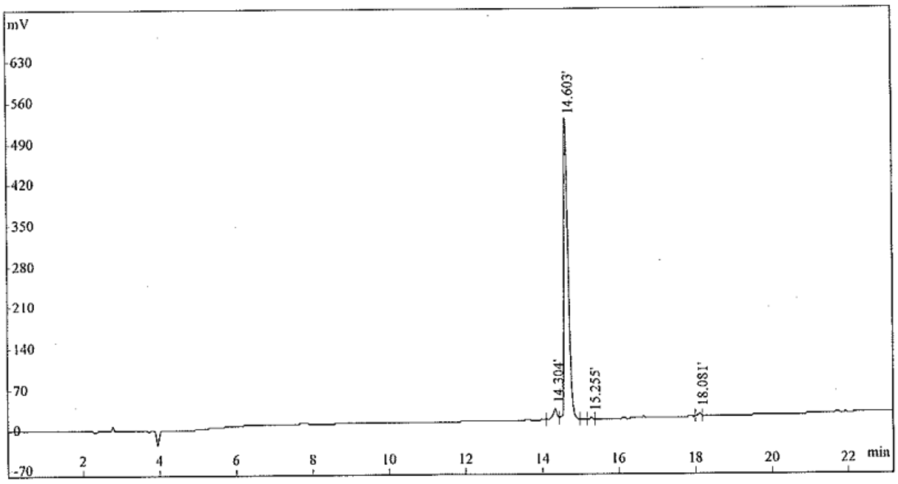

mV
630
560
490
420
350
280
210
140
70
0
-70
14.603'
14.304'
15.255'
18.081'
2
4
6
8
10
12
14
16
18
20
22
min

P44:

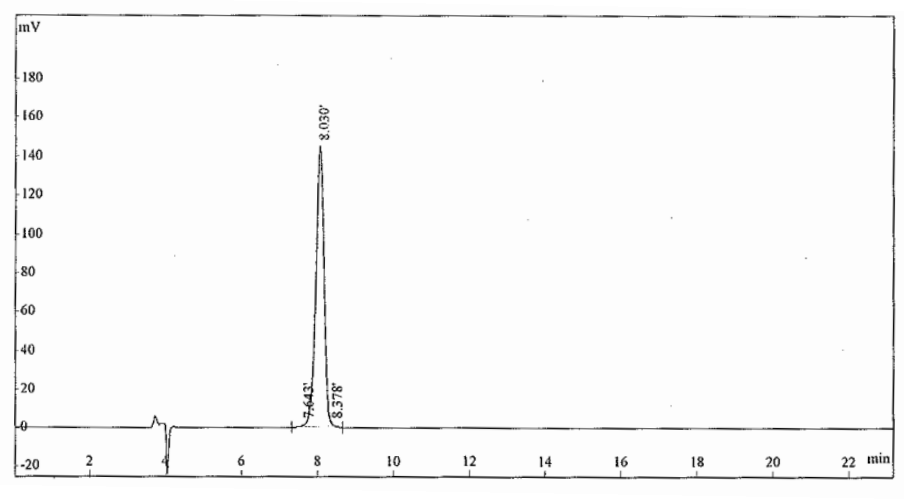

mV
180
160
140
120
100
80
60
40
20
0
-20
8.030'
7.643'
8.378'
2
4
6
8
10
12
14
16
18
20
22
min

P45:

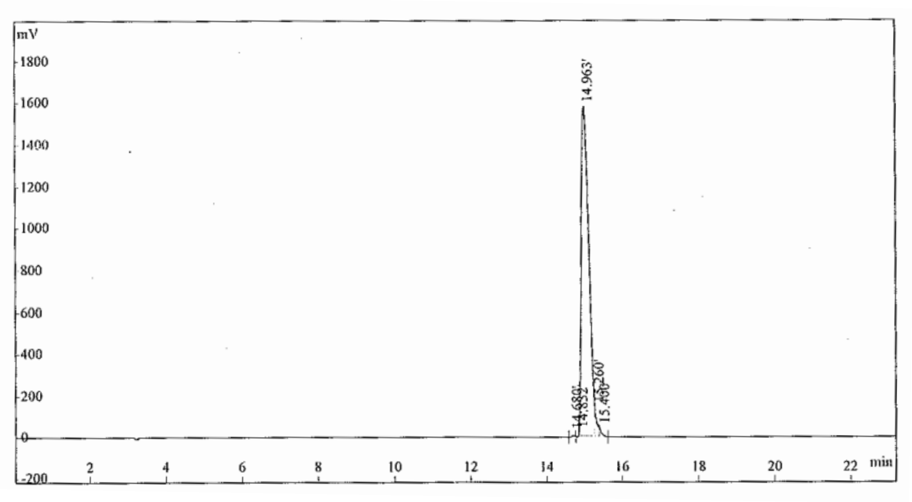

mV
1800
1600
1400
1200
1000
800
600
400
200
0
-200
14.963'
2
4
6
8
10
12
14
16
18
20
22
min

P46:

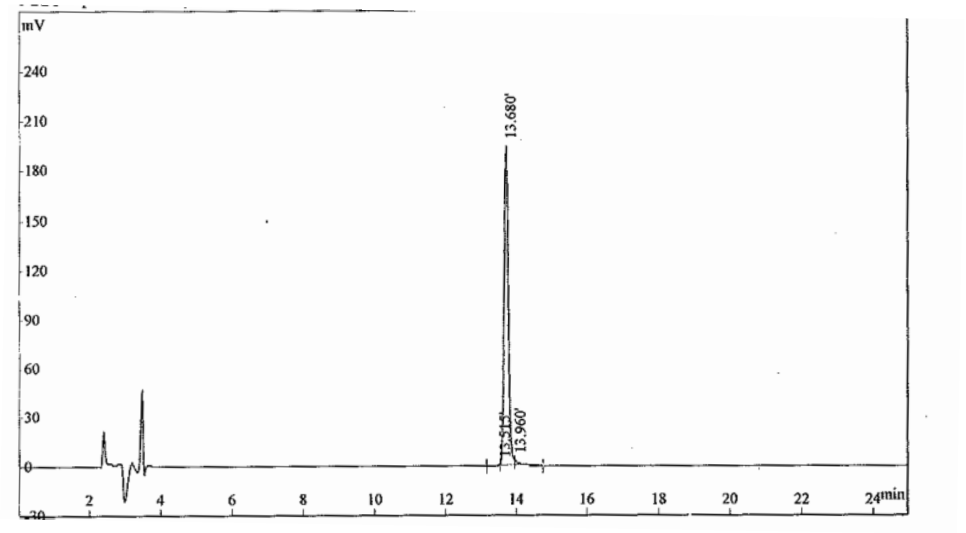

P150:

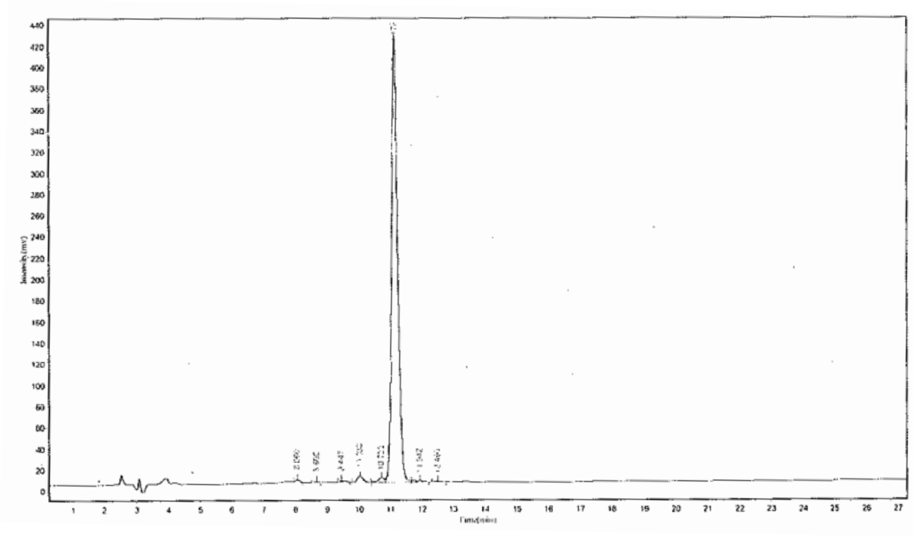

P151:

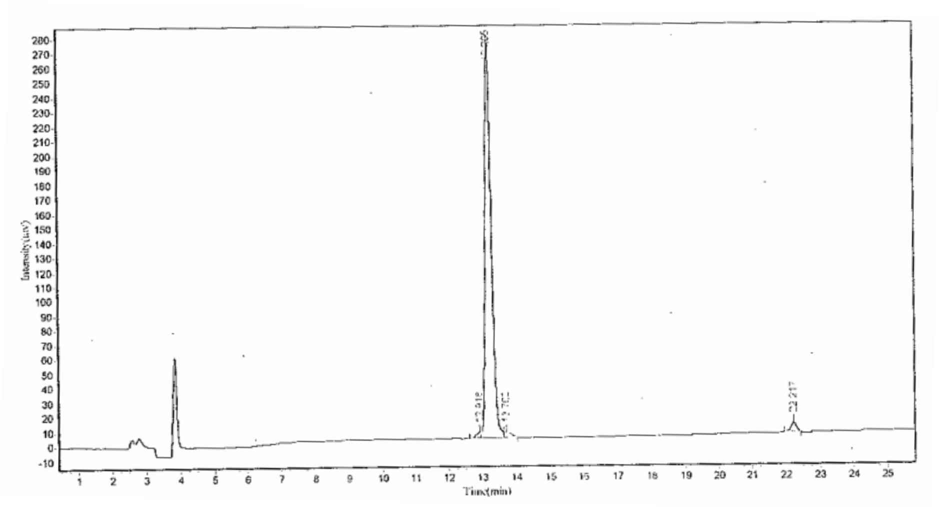

P152:

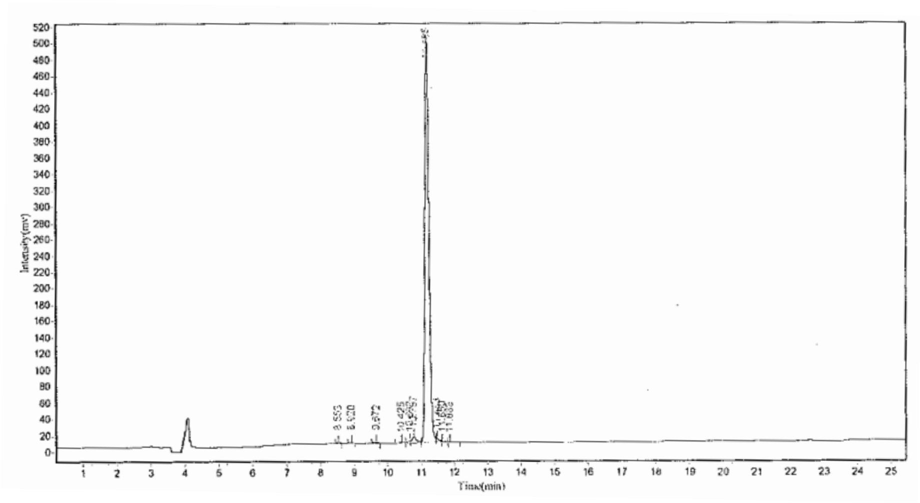

P153:

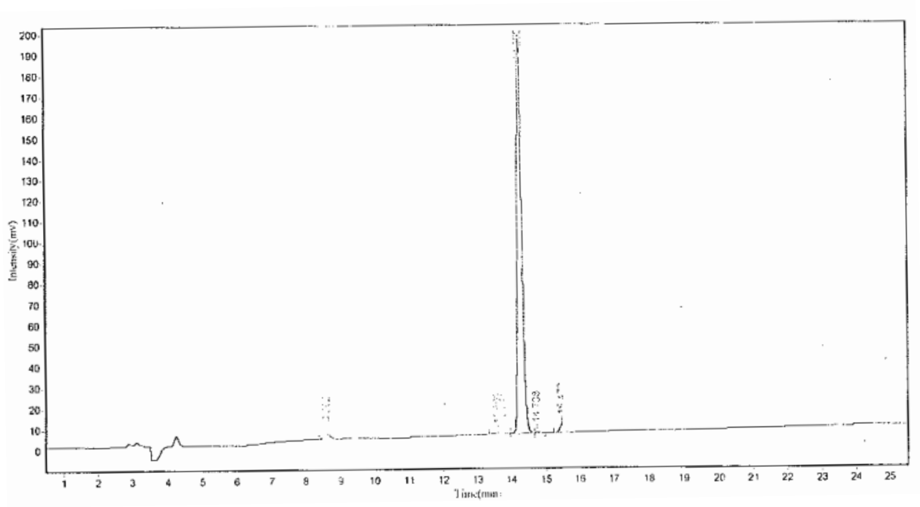

P154:

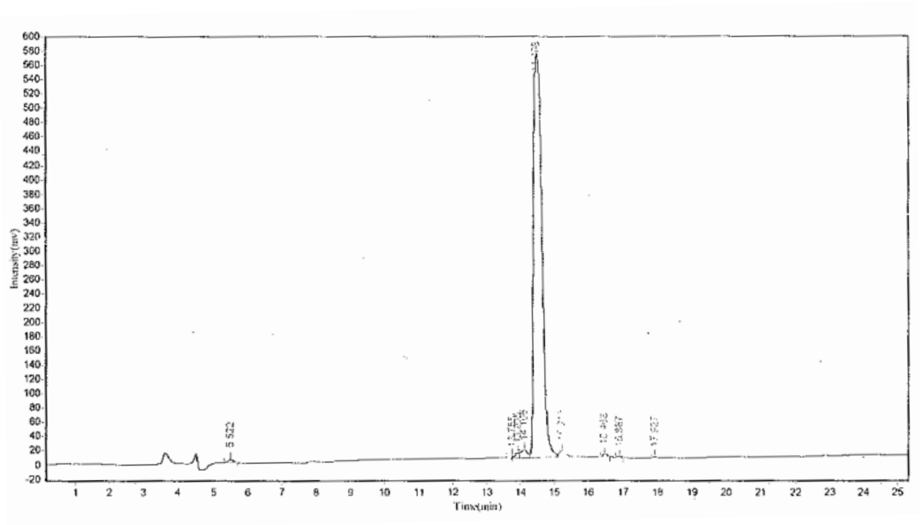

Gp100 (106 peptides, P46-2, P47~P136, P155~P169)

P46-2:

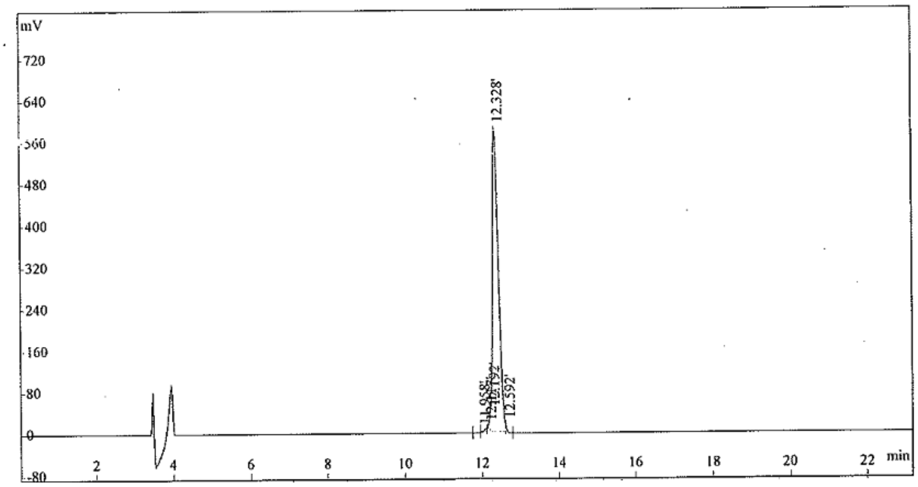

P47:

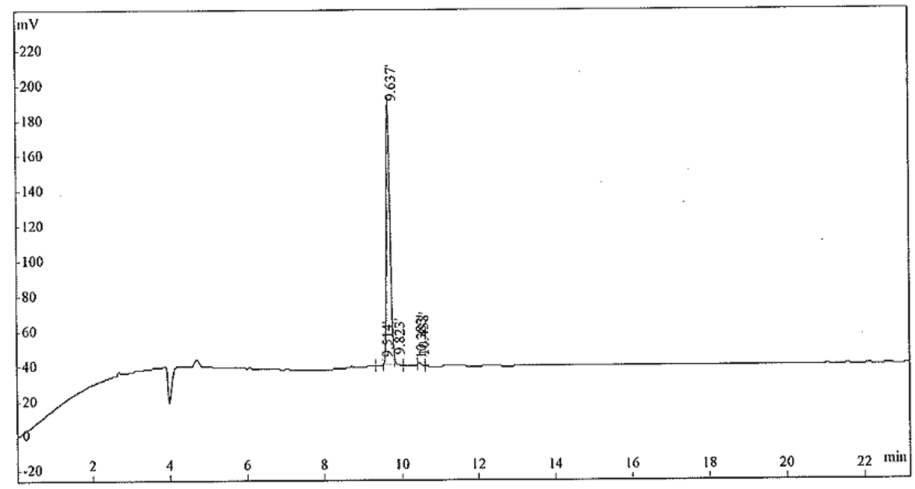

P48:

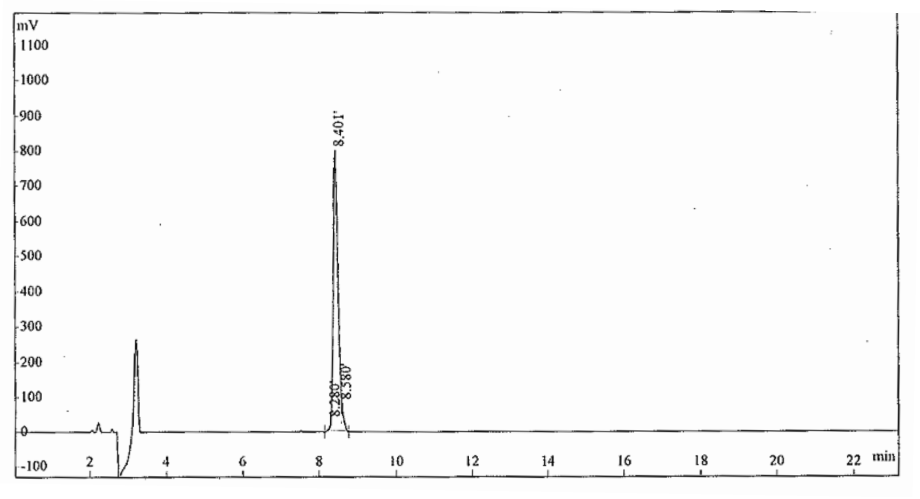

P49:

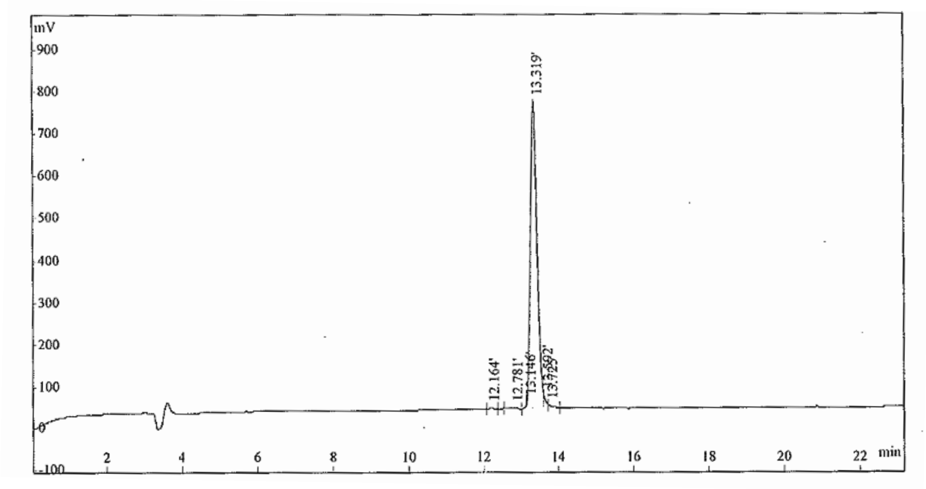
mV
900
800
700
600
500
400
300
200
100
0
-100
13.319'
12.164'
12.781'
13.146'
13.592'
13.729'
2
4
6
8
10
12
14
16
18
20
22
min

P50:

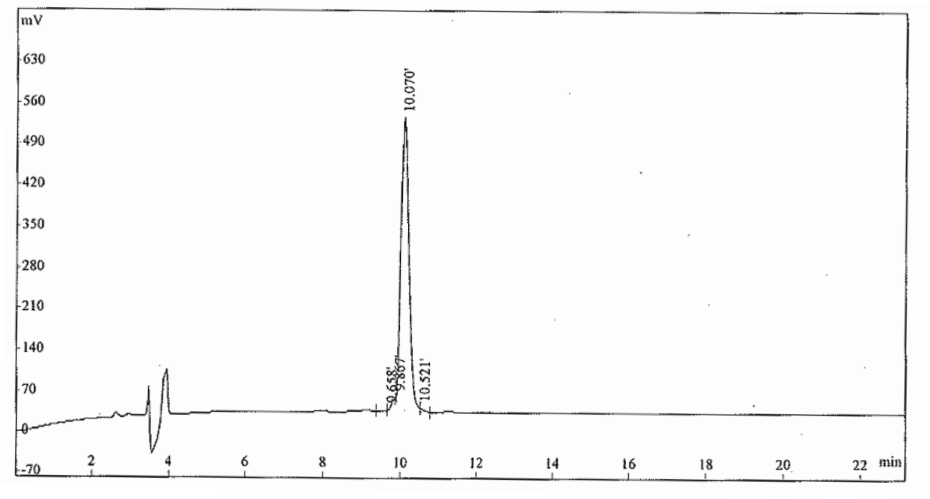
mV
630
560
490
420
350
280
210
140
70
0
-70
10.070'
9.658'
9.867'
10.521'
2
4
6
8
10
12
14
16
18
20
22
min

P51:

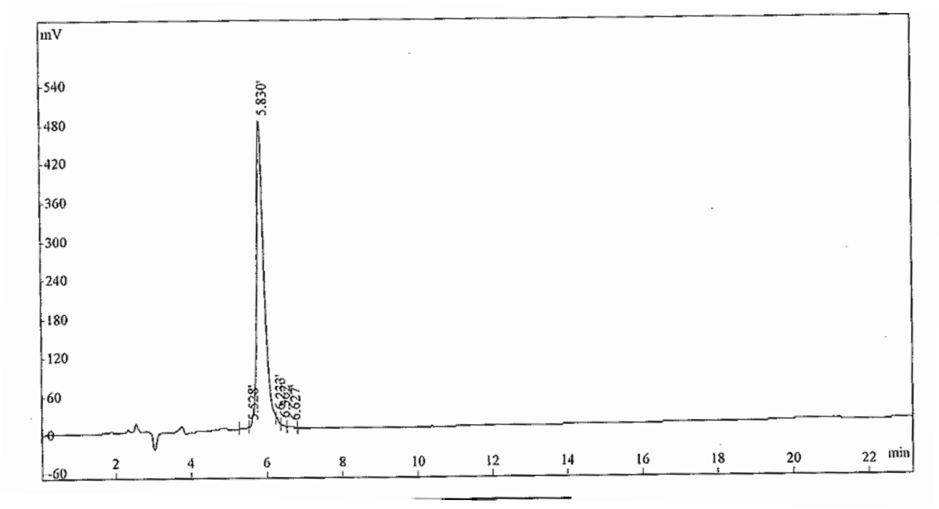
mV
540
480
420
360
300
240
180
120
60
0
-60
5.830'
5.528'
6.238'
6.627'
2
4
6
8
10
12
14
16
18
20
22
min

P52:

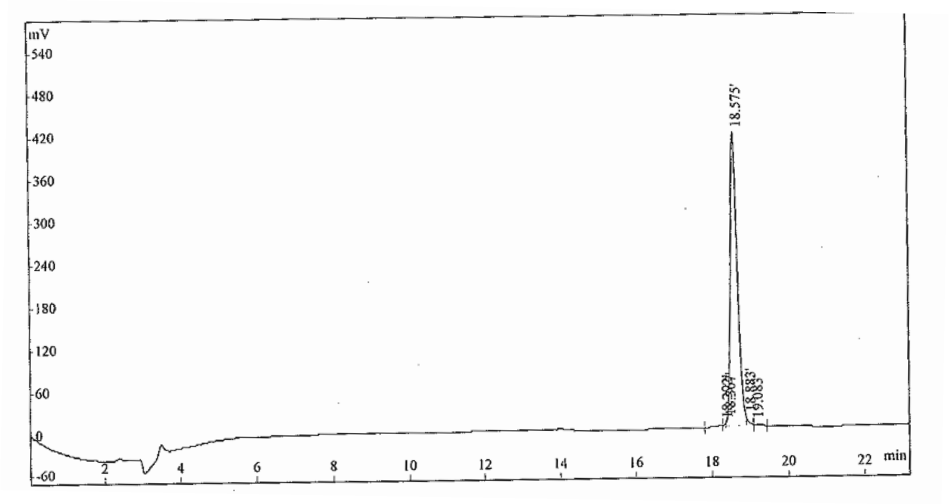
mV
540
480
420
360
300
240
180
120
60
0
-60
18.575'
18.883'
19.083'
2
4
6
8
10
12
14
16
18
20
22
min

P53:

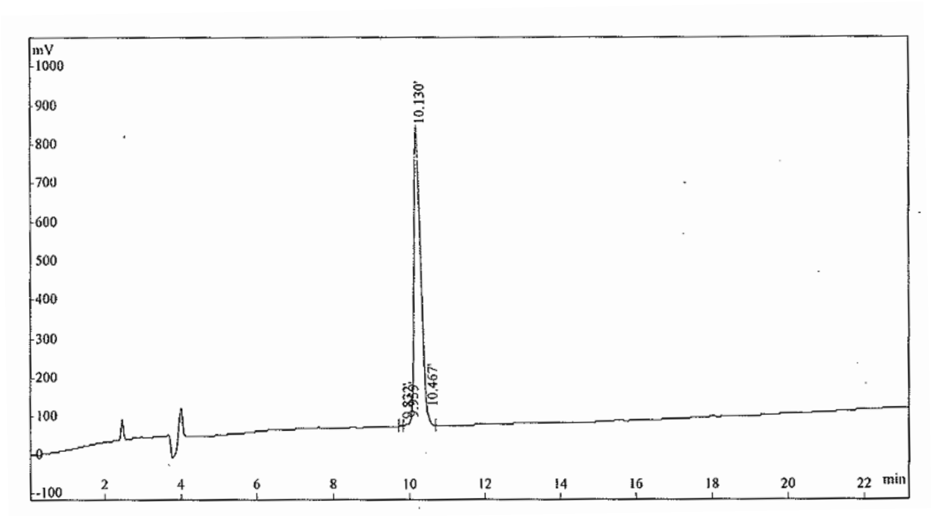
mV
1000
900
800
700
600
500
400
300
200
100
0
-100
10.130'
10.467'
2
4
6
8
10
12
14
16
18
20
22
min

P54:

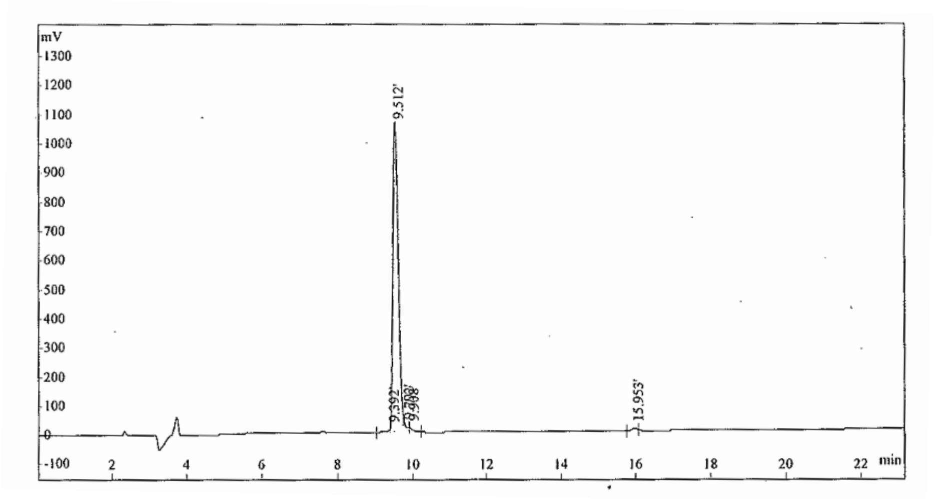
mV
1300
1200
1100
1000
900
800
700
600
500
400
300
200
100
0
-100
9.512'
9.392'
15.953'
2
4
6
8
10
12
14
16
18
20
22
min

P55:

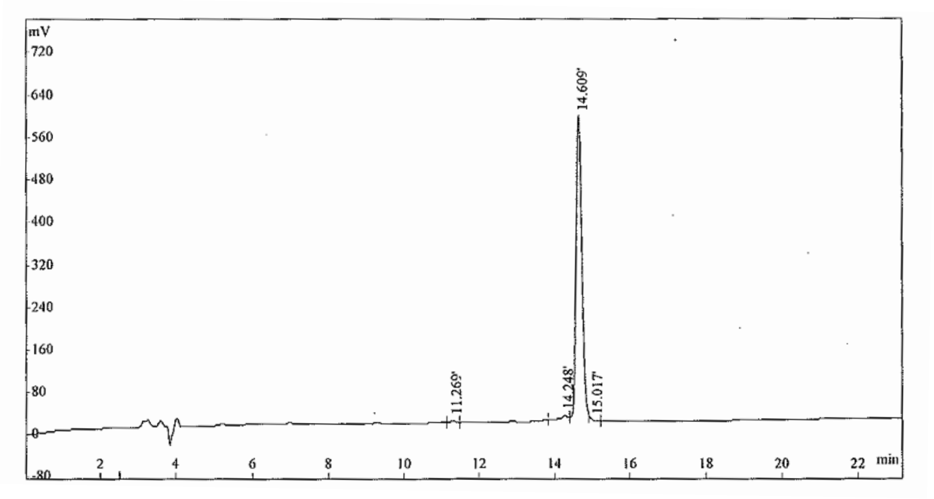
mV
720
640
560
480
400
320
240
160
80
0
-80
2
4
6
8
10
12
14
16
18
20
22
min
14.609'
11.269'
14.248'
15.017'

P56:

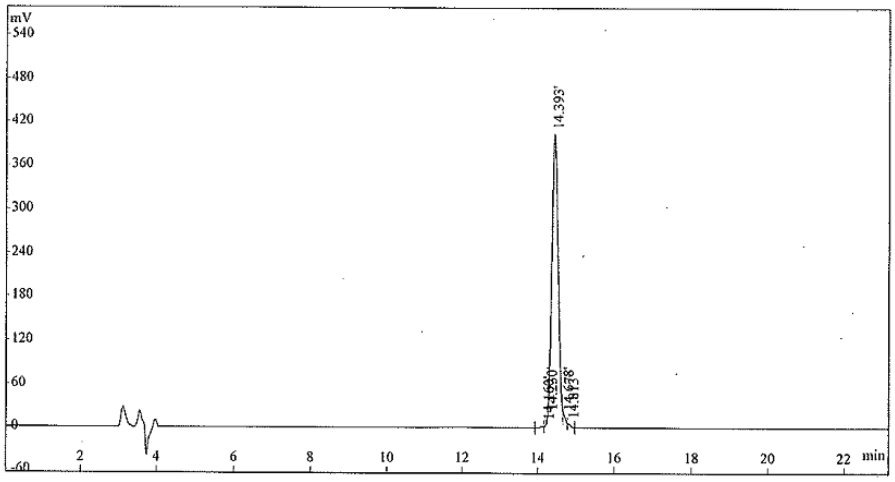
mV
540
480
420
360
300
240
180
120
60
0
-60
2
4
6
8
10
12
14
16
18
20
22
min
14.393'

P57:

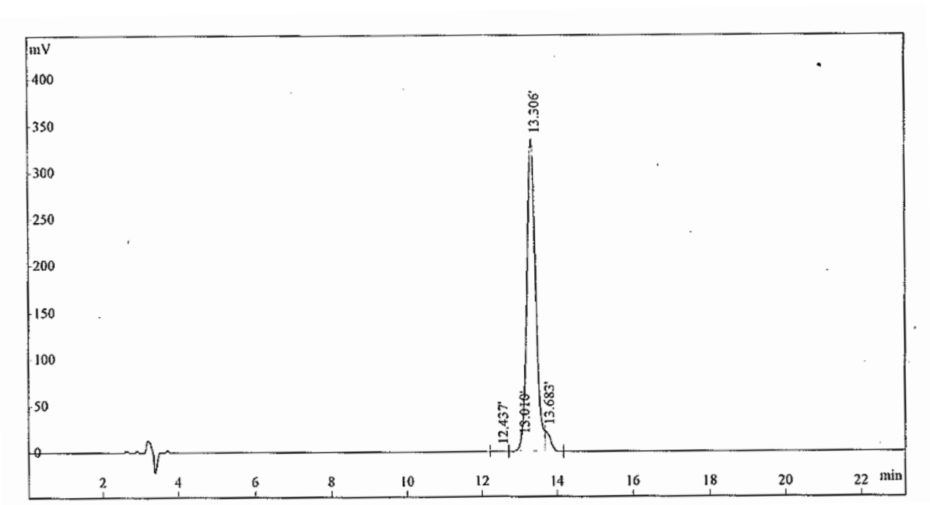
mV
400
350
300
250
200
150
100
50
0
2
4
6
8
10
12
14
16
18
20
22
min
13.306'
12.437'
13.010'
13.683'

P58:

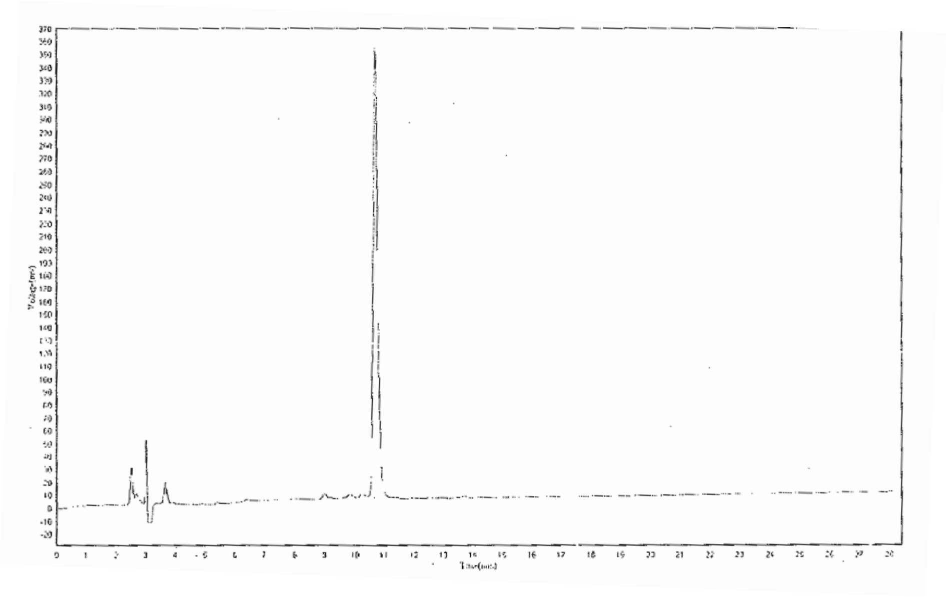

P59:

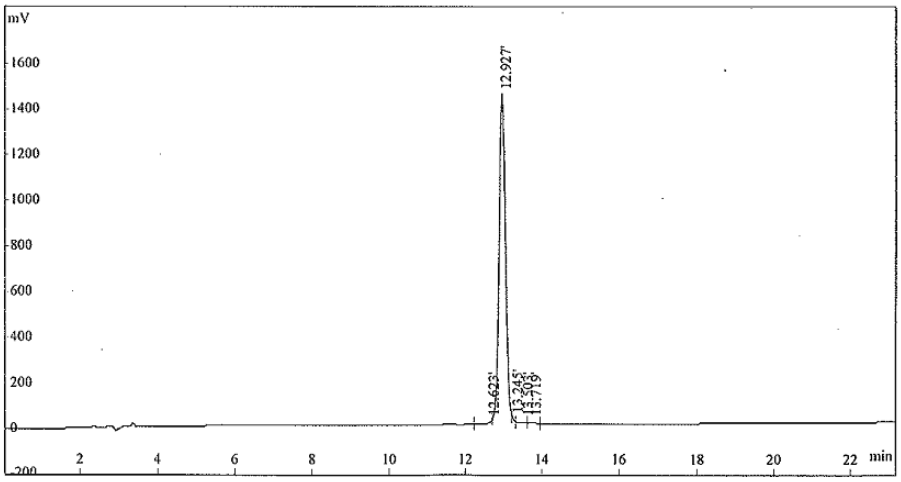

mV
1600
1400
1200
1000
800
600
400
200
0
-200
12.927'
12.623'
13.245'
13.719'
2
4
6
8
10
12
14
16
18
20
22
min

P60:

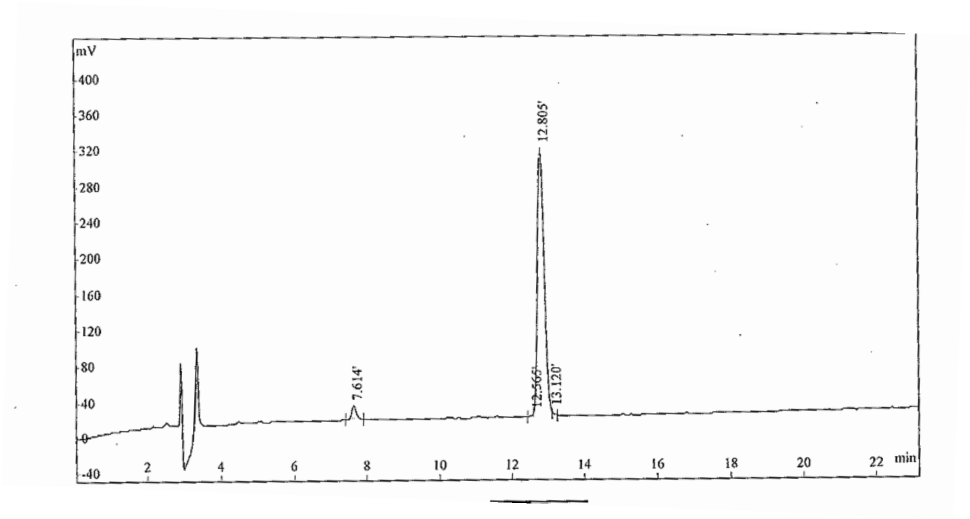

mV
400
360
320
280
240
200
160
120
80
40
0
-40
7.614'
12.805'
12.565'
13.120'
2
4
6
8
10
12
14
16
18
20
22
min

P61:

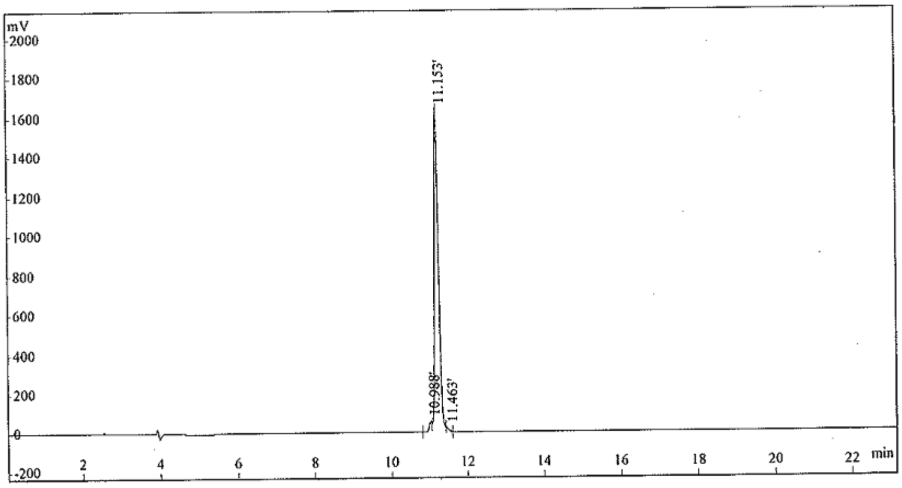

mV
2000
1800
1600
1400
1200
1000
800
600
400
200
0
-200
11.153'
10.988'
11.463'
2
4
6
8
10
12
14
16
18
20
22
min

P62:

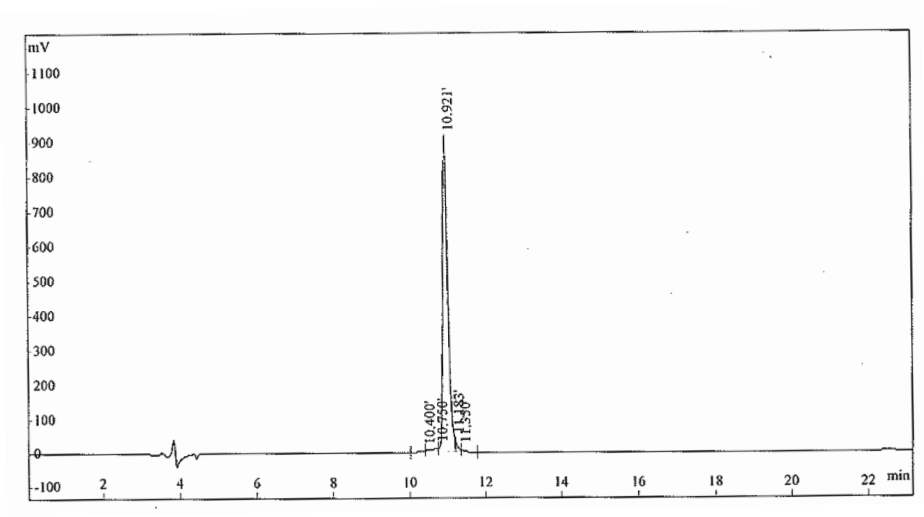

mV
1100
1000
900
800
700
600
500
400
300
200
100
0
-100
10.921'
10.400'
10.750'
11.183'
11.550'
2
4
6
8
10
12
14
16
18
20
22
min

P63:

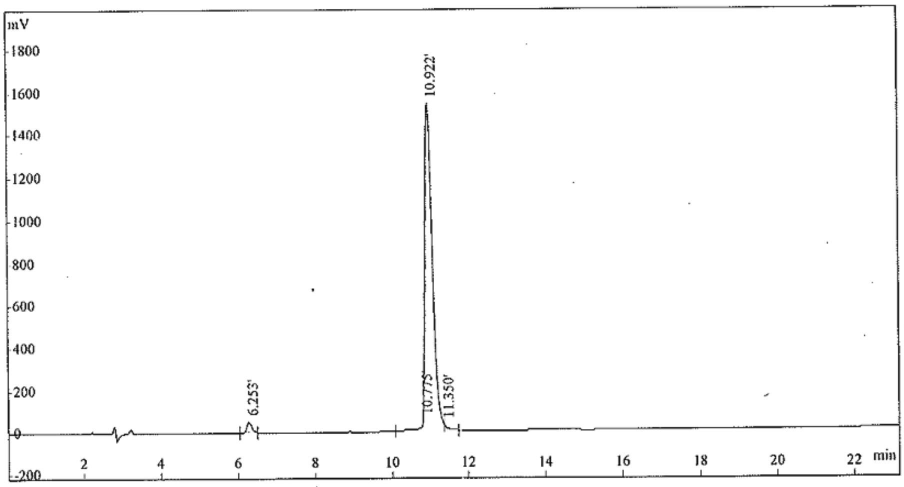

mV
1800
1600
1400
1200
1000
800
600
400
200
0
-200
10.922'
6.253'
10.775'
11.350'
2
4
6
8
10
12
14
16
18
20
22
min

P64:

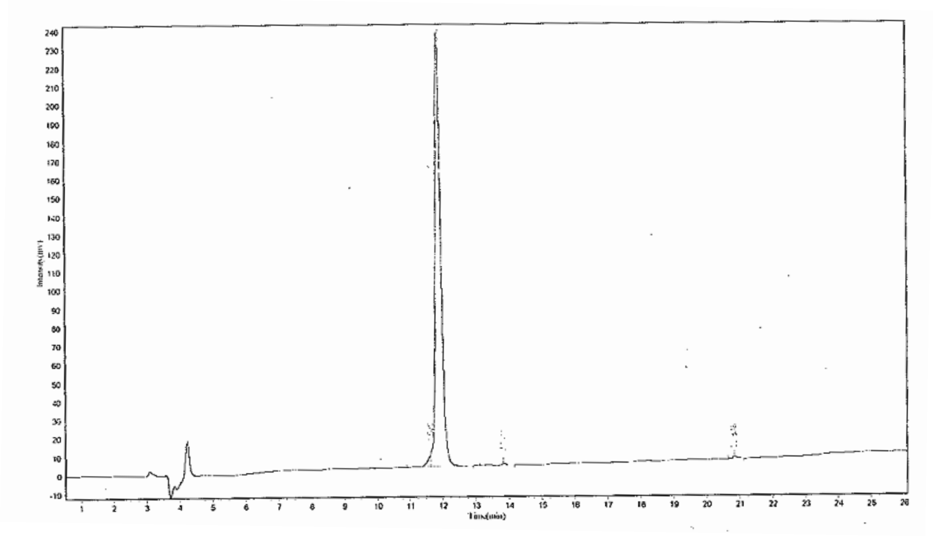

P65:

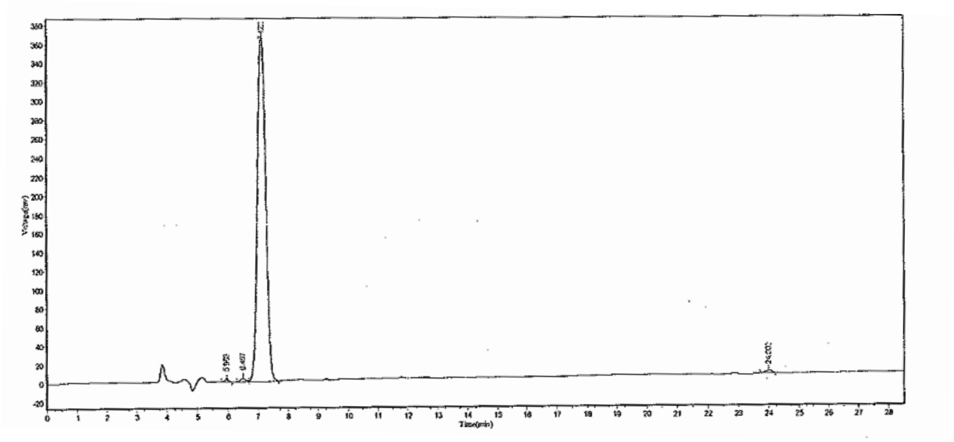

P66:

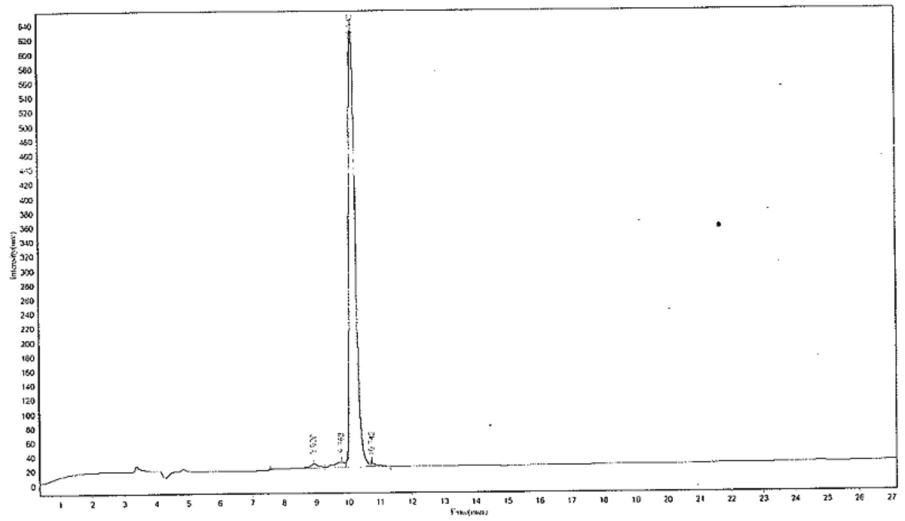

P67:

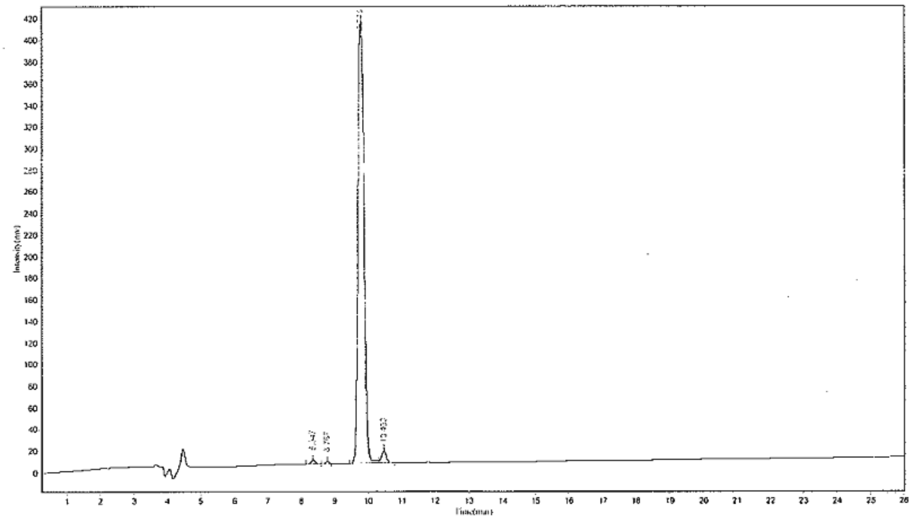

P68:

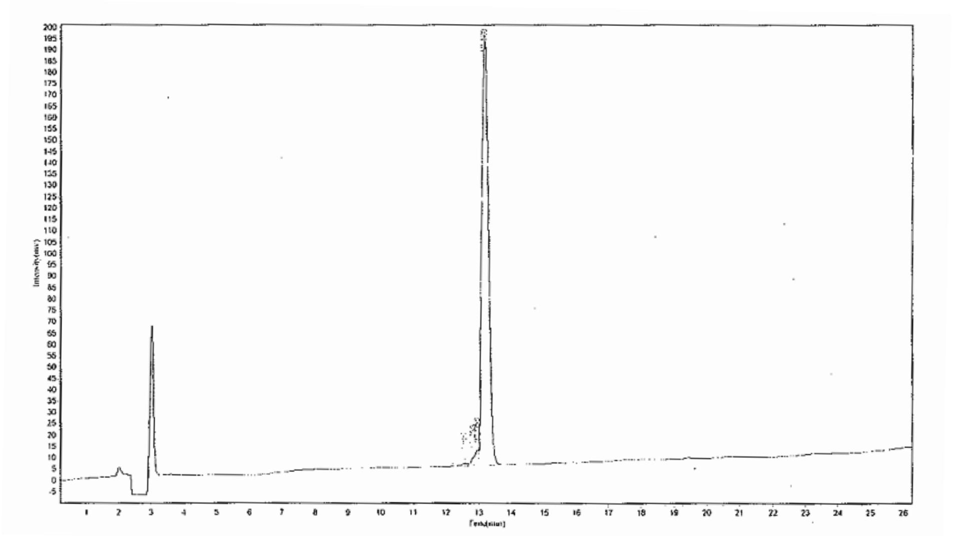

P69:

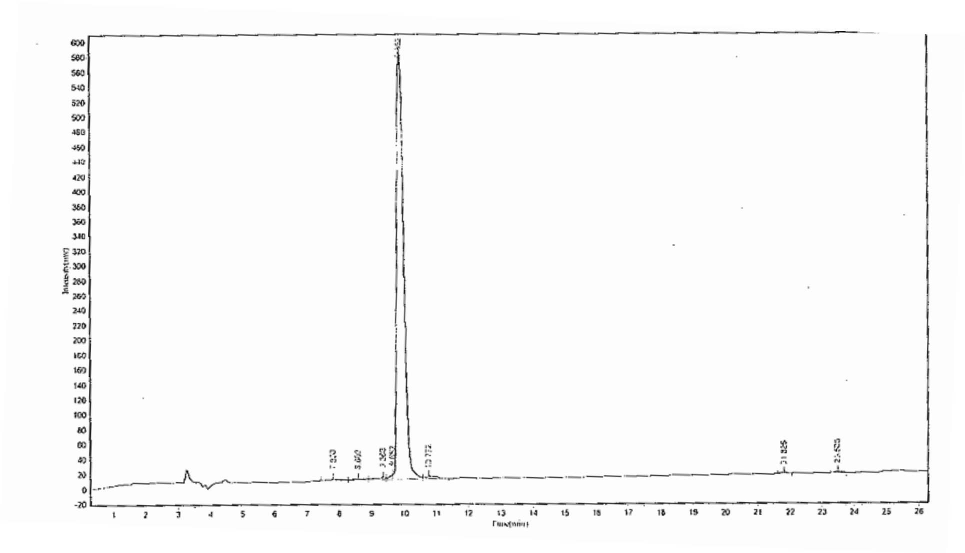

P70:

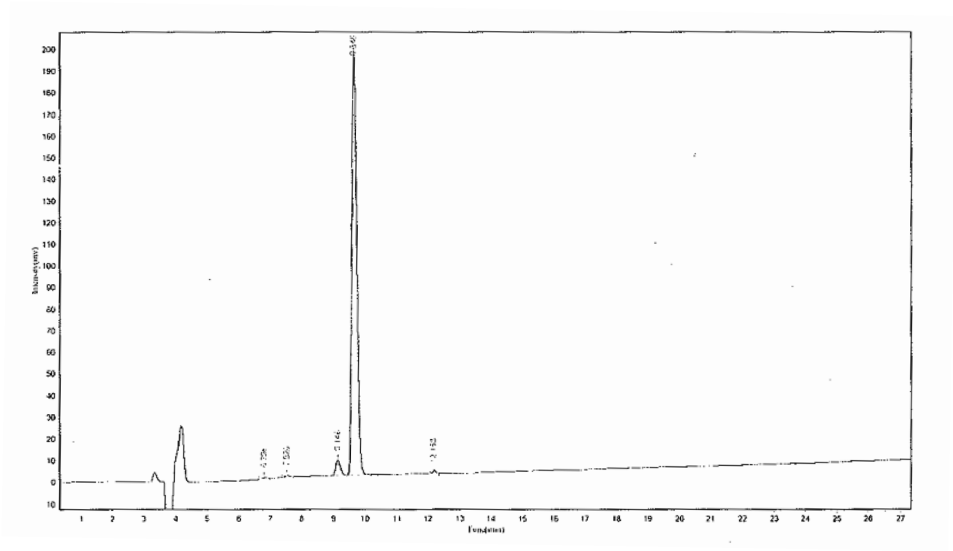

P71:

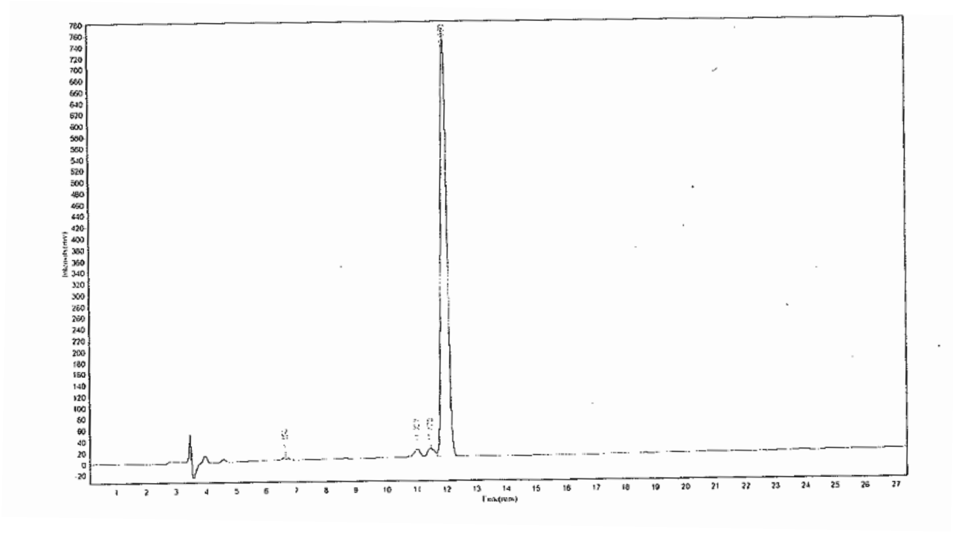

P72:

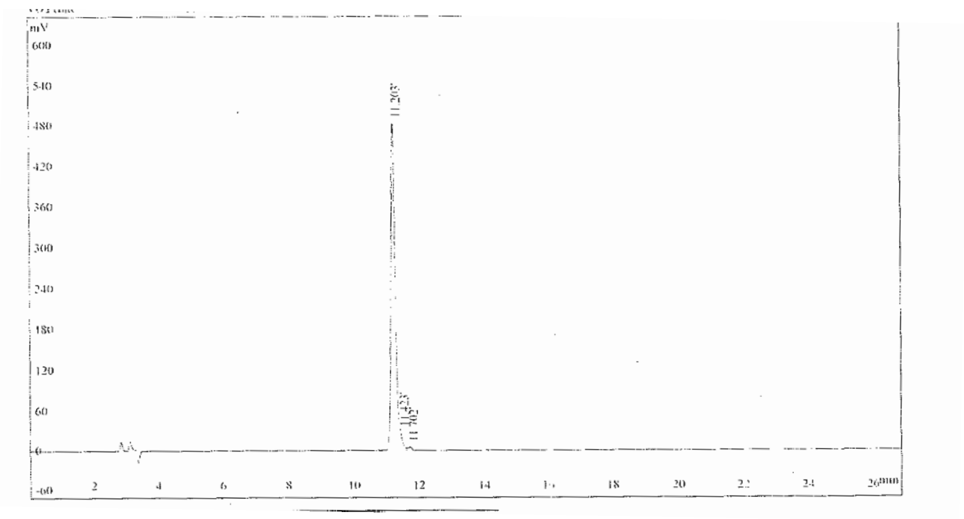
mV
600
540
480
420
360
300
240
180
120
60
0
-60
2
4
6
8
10
12
14
18
20
24
min

P73:

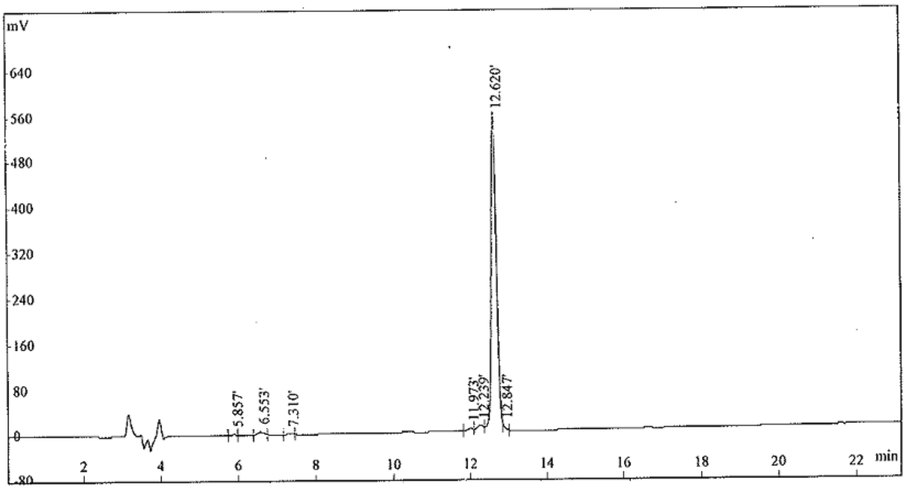

mV
640
560
480
400
320
240
160
80
0
-80
5.857'
6.553'
7.310'
11.973'
12.259'
12.620'
12.847'
2
4
6
8
10
12
14
16
18
20
22
min

P74:

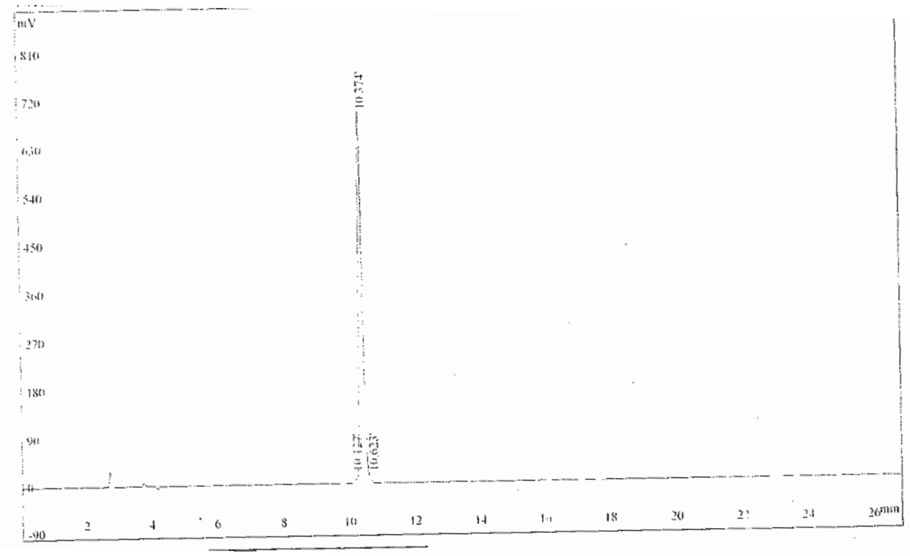

mV
810
720
630
540
450
360
270
180
90
10.374'
10.623'
-90
2
4
6
8
10
12
14
18
20
24
26min

P75:

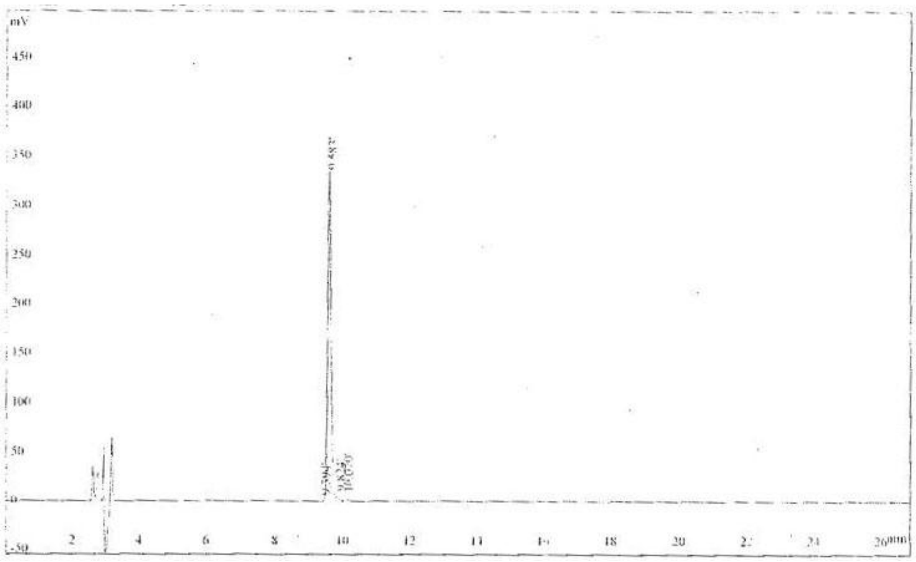

mV
450
400
350
300
250
200
150
100
50
-50
2
4
6
8
10
12
18
20
24
26min

P76:

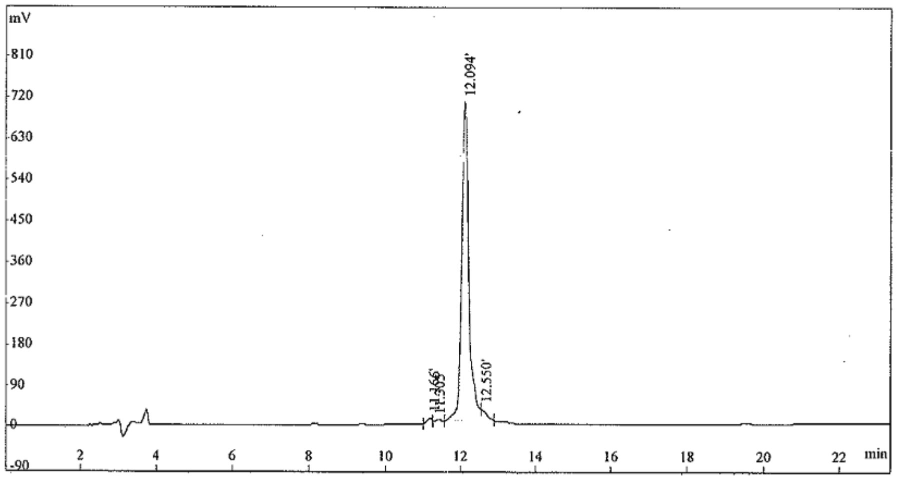
mV
810
720
630
540
450
360
270
180
90
0
-90
12.094'
12.550'
2
4
6
8
10
12
14
16
18
20
22
min

P77:

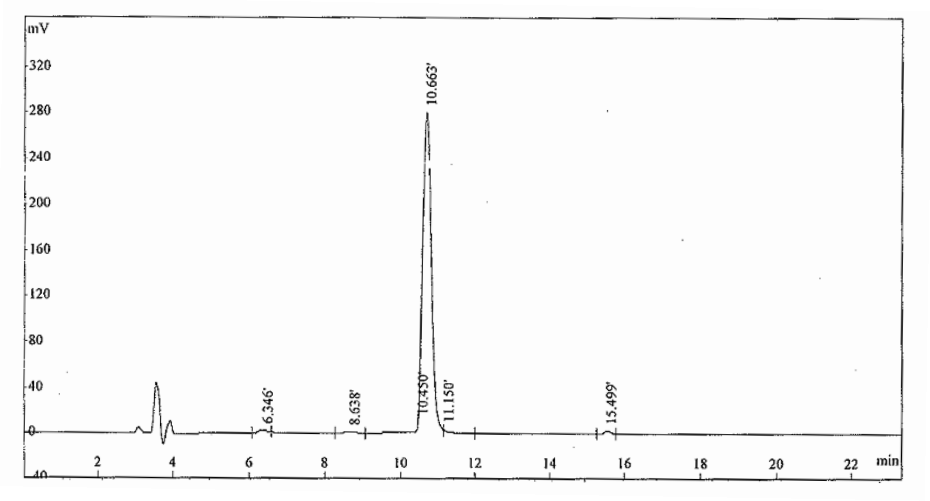
mV
320
280
240
200
160
120
80
40
0
-40
10.663'
6.346'
8.638'
10.450'
11.150'
15.499'
2
4
6
8
10
12
14
16
18
20
22
min

P78:

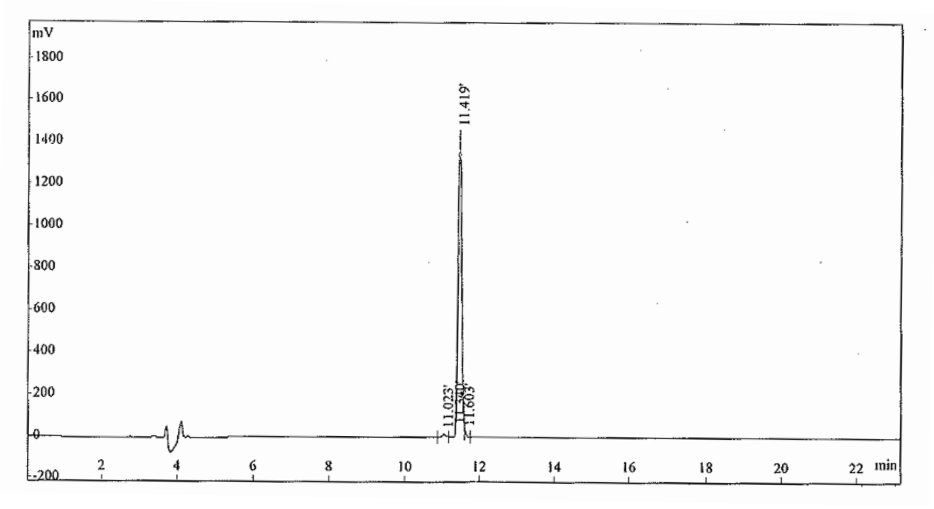
mV
1800
1600
1400
1200
1000
800
600
400
200
0
-200
11.419'
11.023'
11.603'
2
4
6
8
10
12
14
16
18
20
22
min

P79:

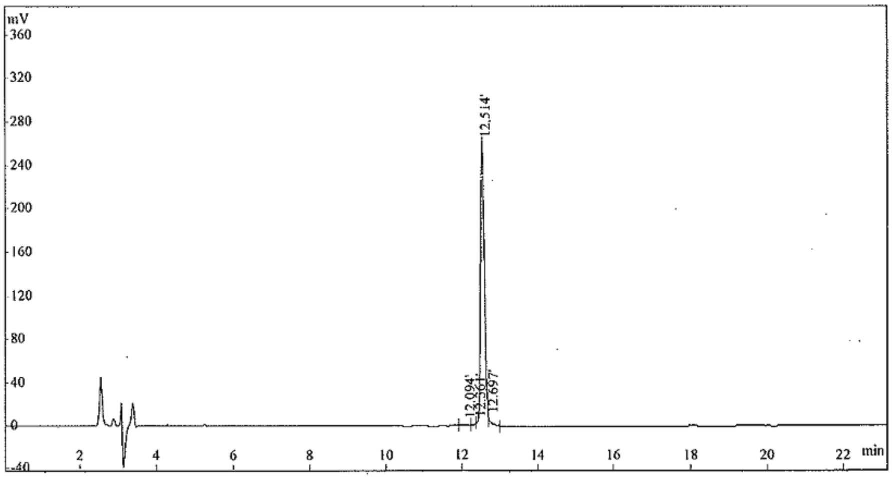
mV
360
320
280
240
200
160
120
80
40
0
-40
2
4
6
8
10
12
14
16
18
20
22
min
12.514'
12.094'
12.697'

P80:

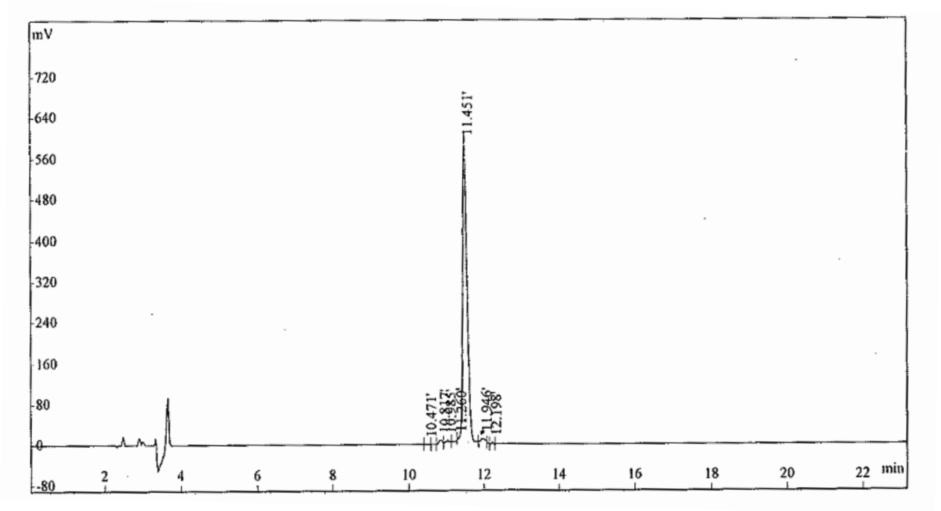
mV
720
640
560
480
400
320
240
160
80
0
-80
2
4
6
8
10
12
14
16
18
20
22
min
11.451'
10.471'
11.946'
12.198'

P81:

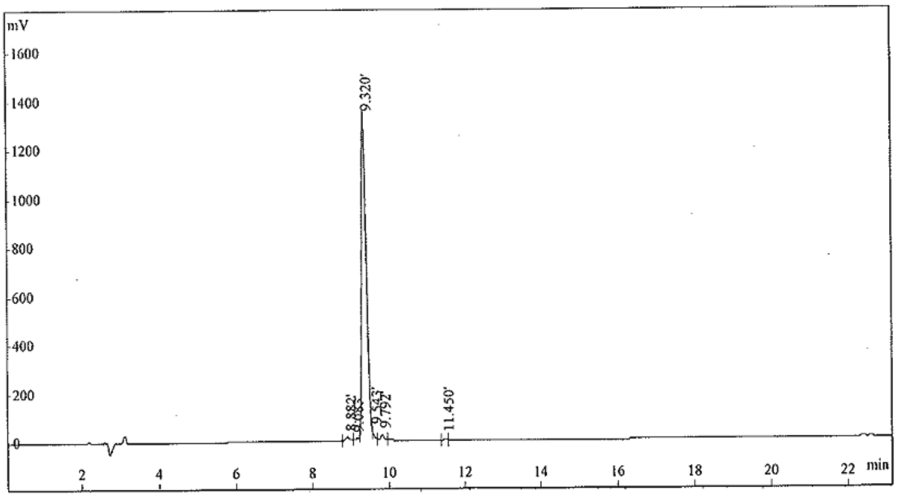
mV
1600
1400
1200
1000
800
600
400
200
0
2
4
6
8
10
12
14
16
18
20
22
min
9.320'
8.882'
9.543'
9.792'
11.450'

P82:

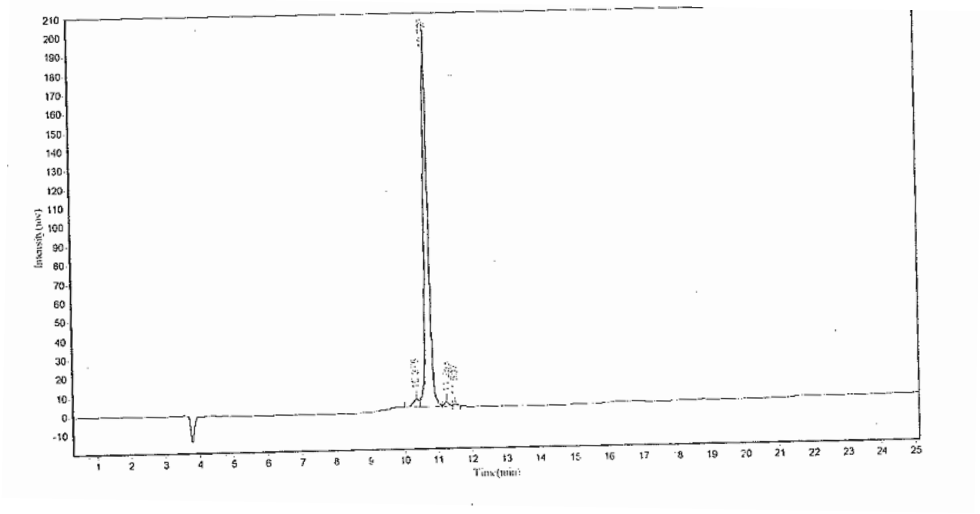
Intensity(mv)
Time(min)

P83:

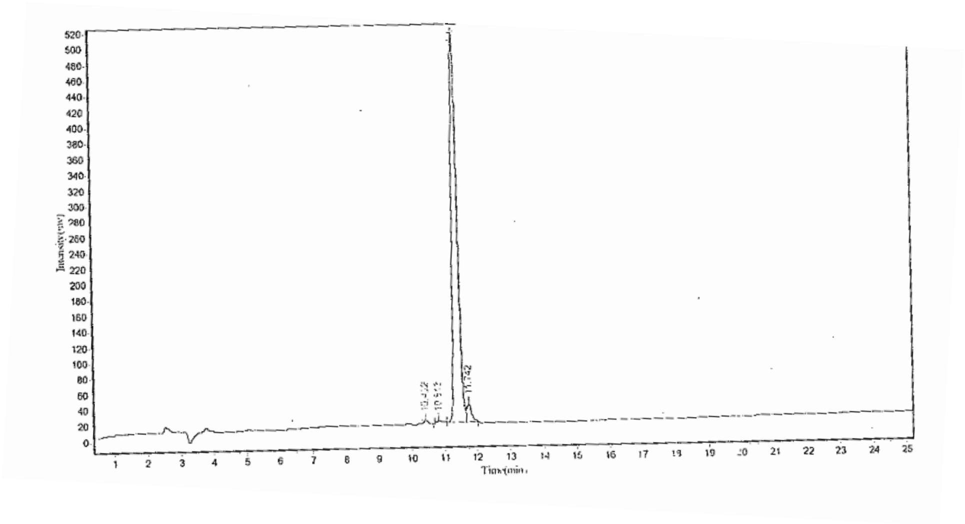
Intensity(mv)
Time(min)
11.742

P84:

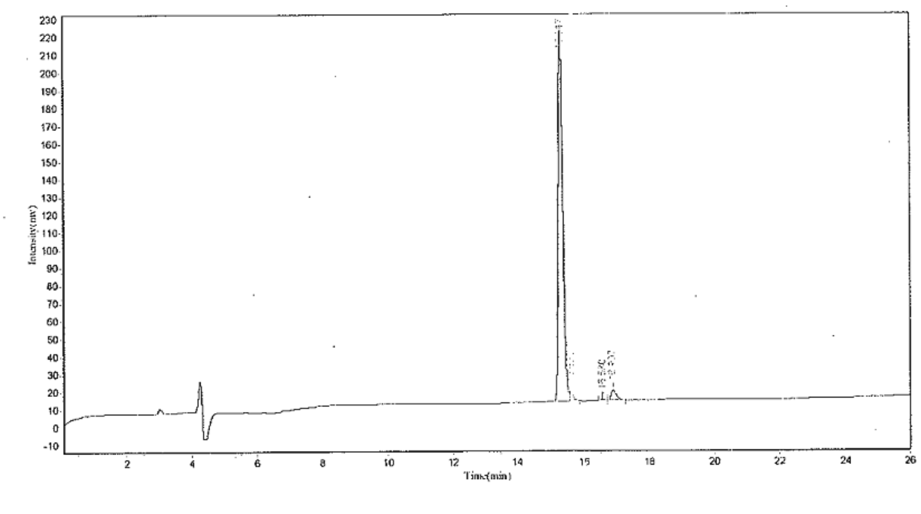
Intensity(mv)
Time(min)

P85:

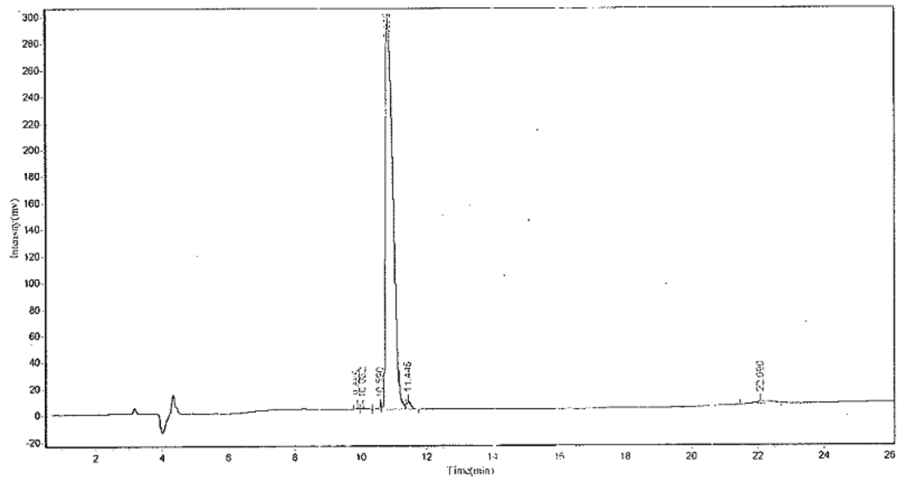

P86:

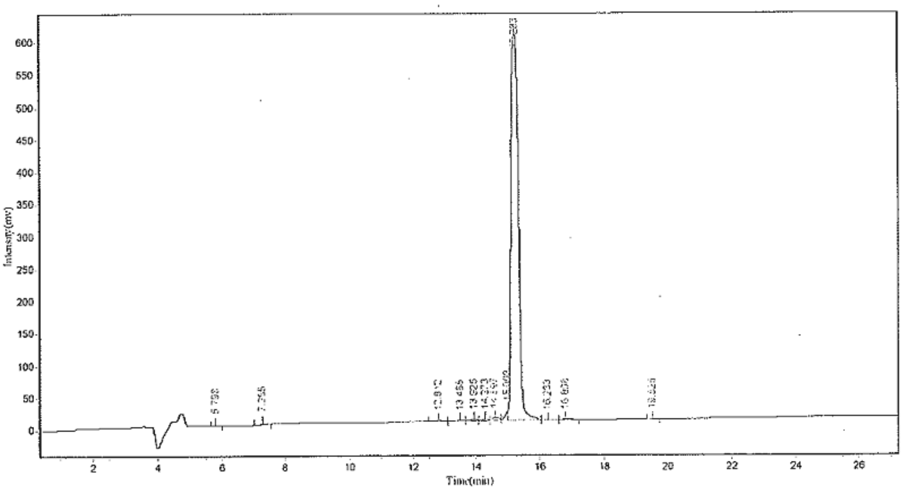

P87:

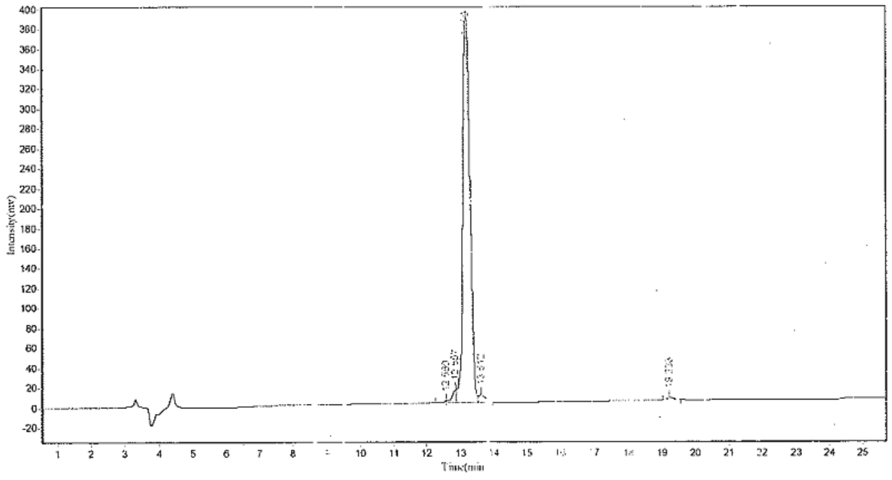

P88:

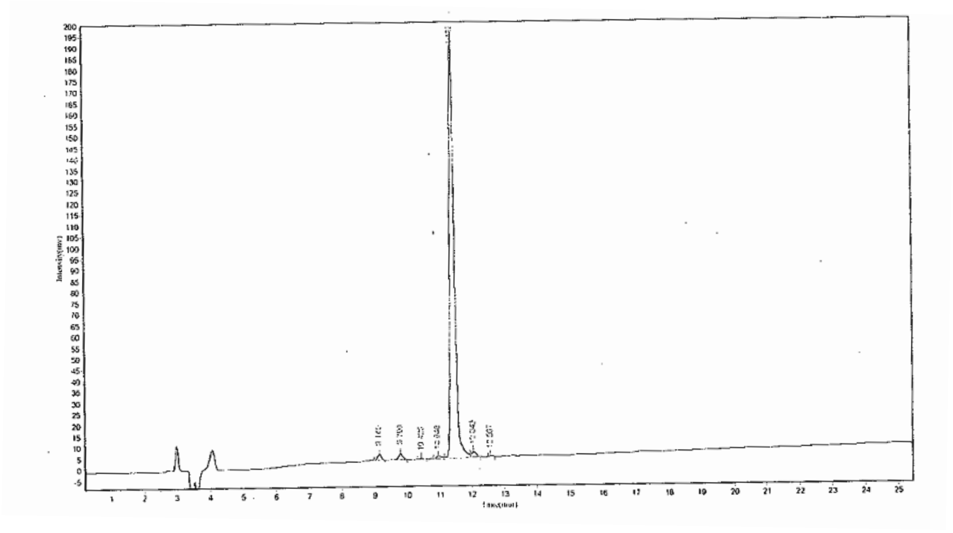

P89:

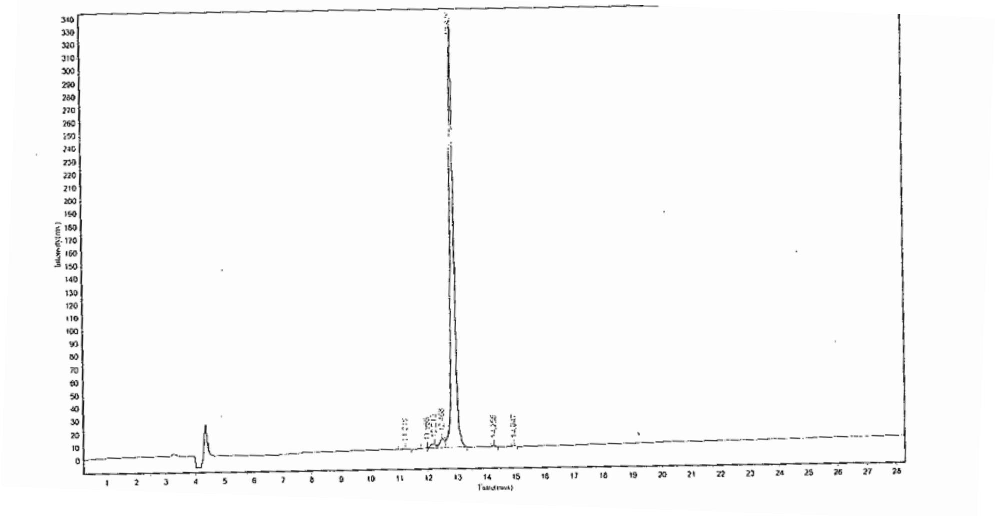

P90:

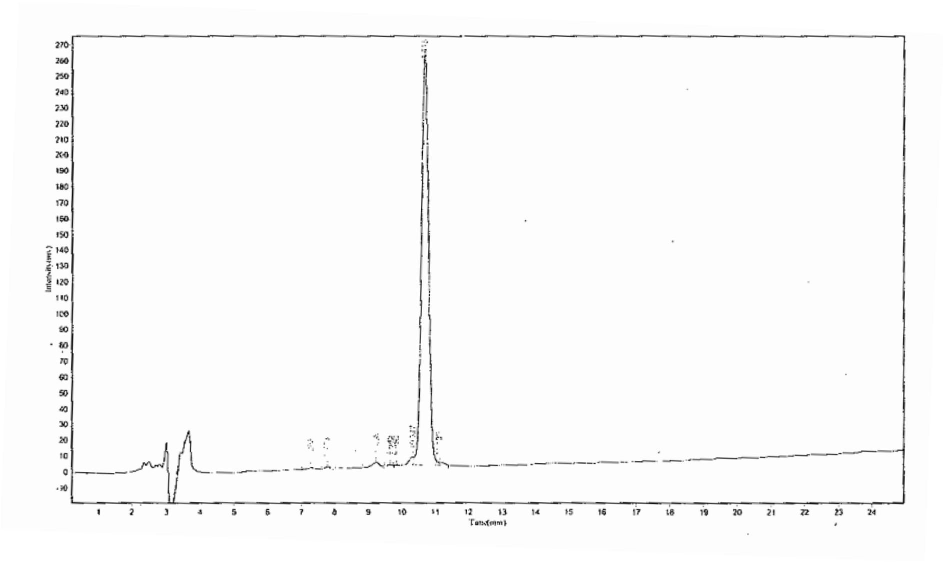

P91:

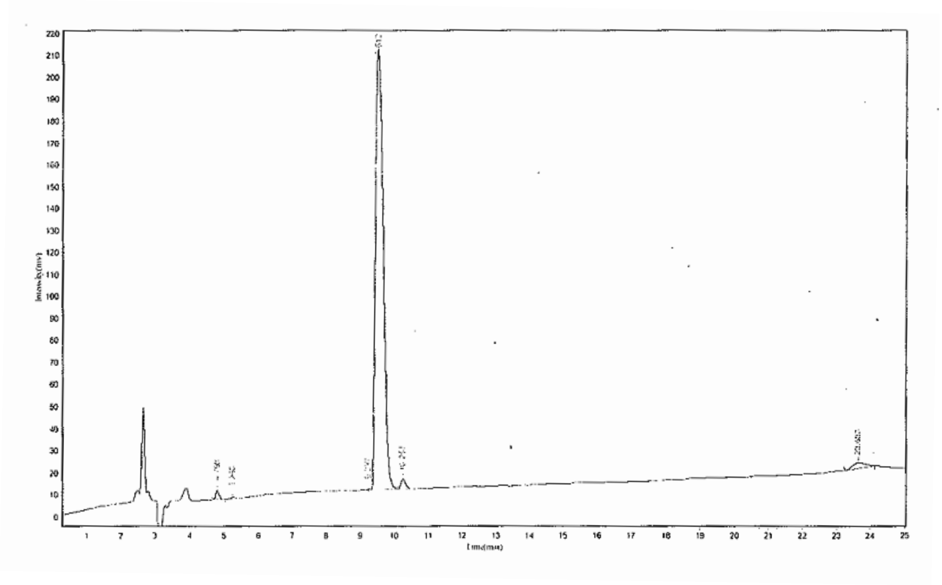

P92:

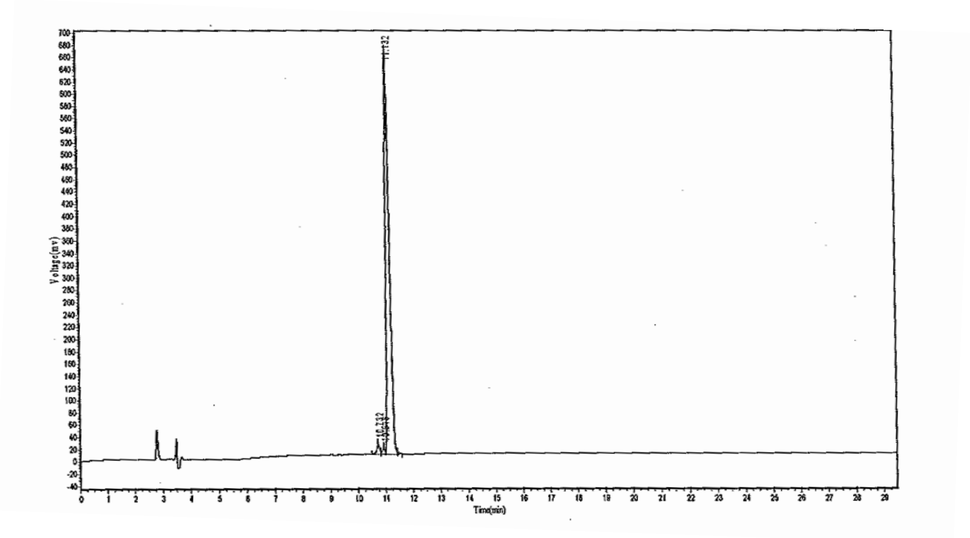

P93:

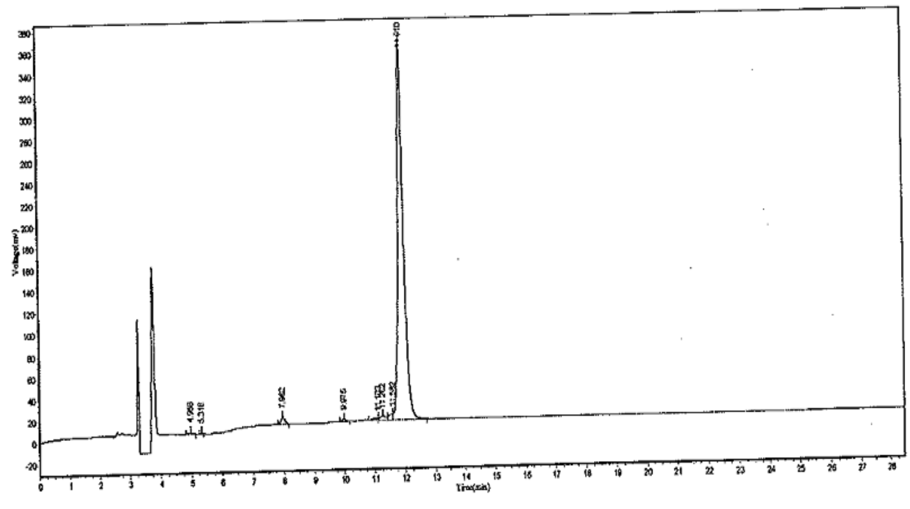

P94:

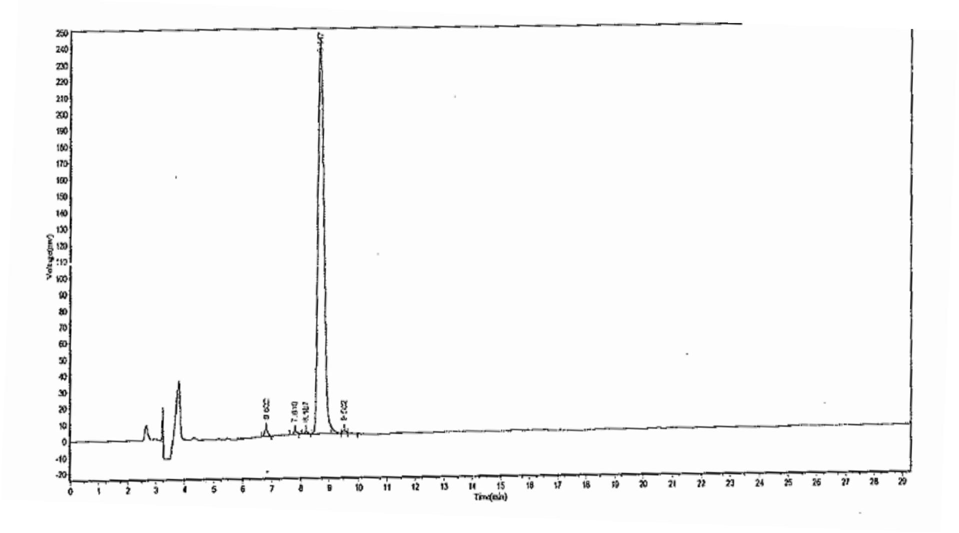

P95:

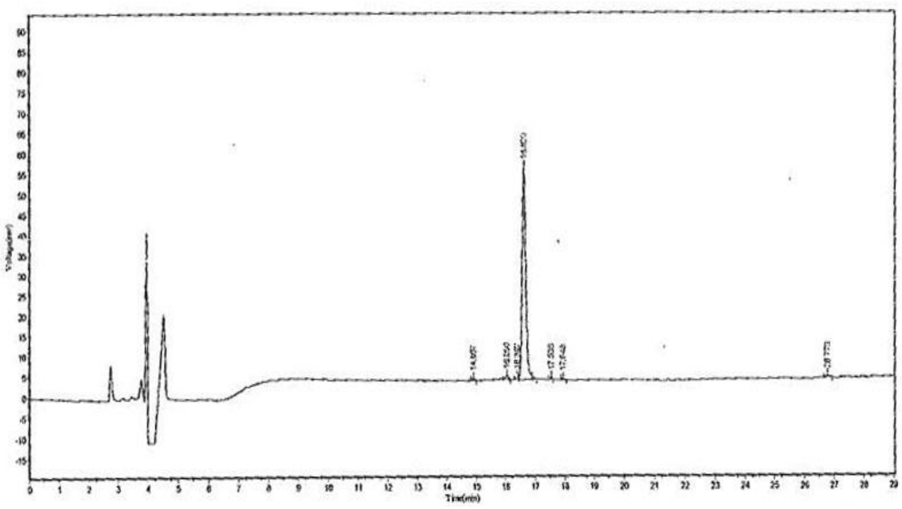

P96:

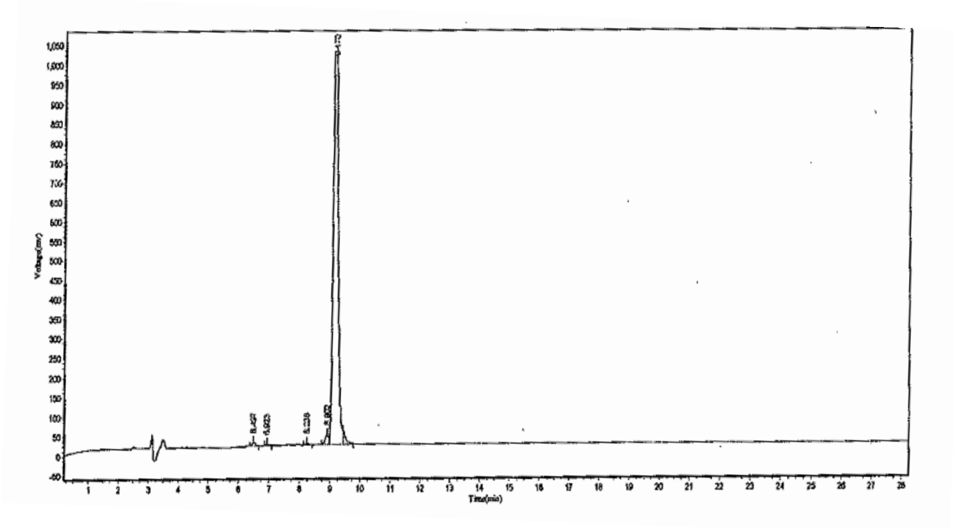

P97:

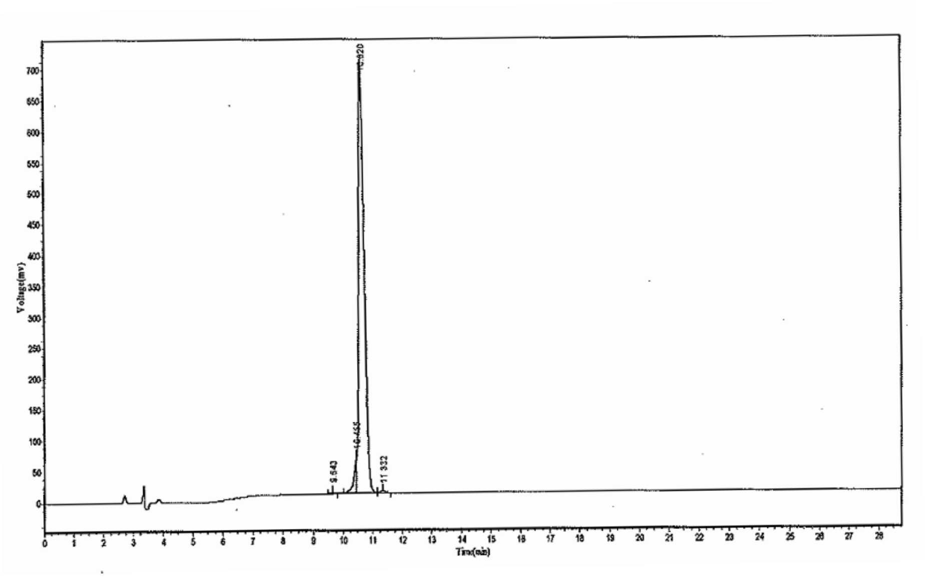
Voltage(mv)
Time(min)
9.643
10.455
11.332

P98:

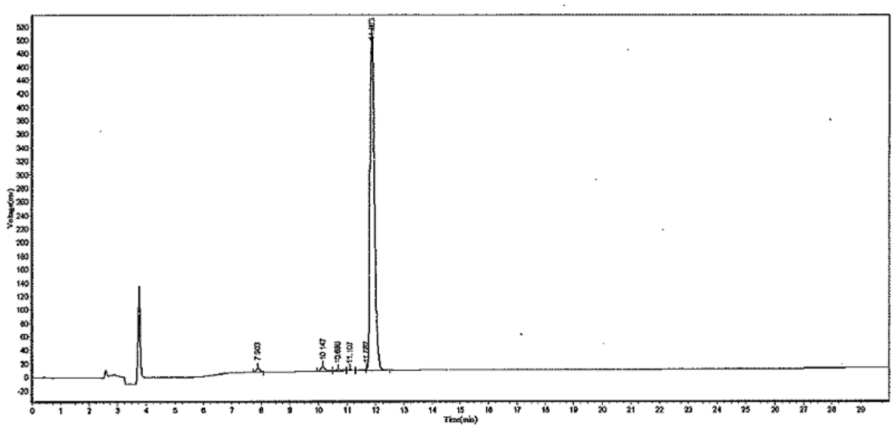
Voltage(mv)
Time(min)
7.903
10.147
11.107

P99:

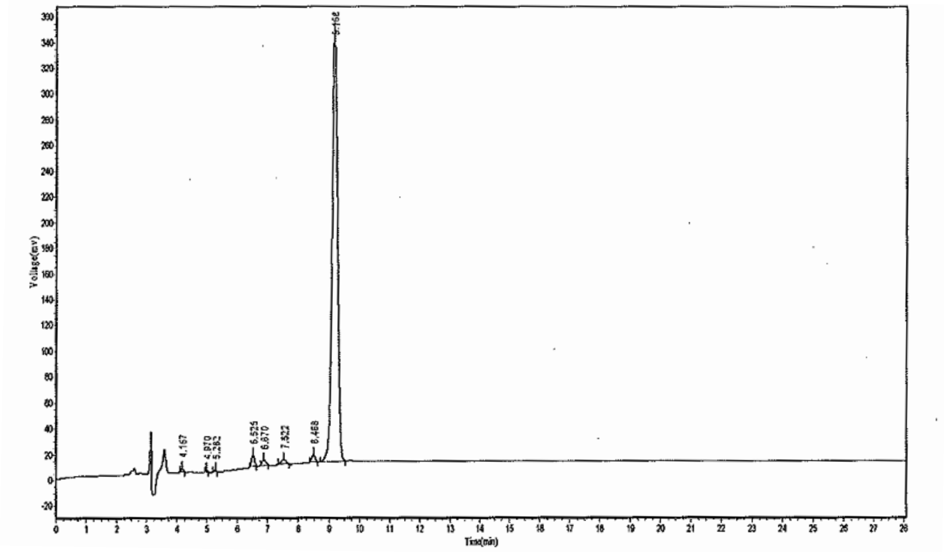
Voltage(mv)
Time(min)
4.167
4.870
5.282
6.525
6.870
7.522
8.458

P100:

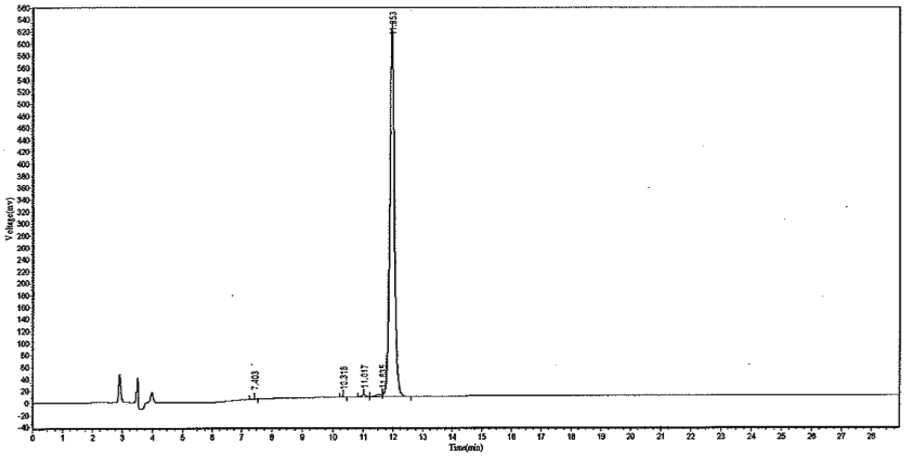

P101:

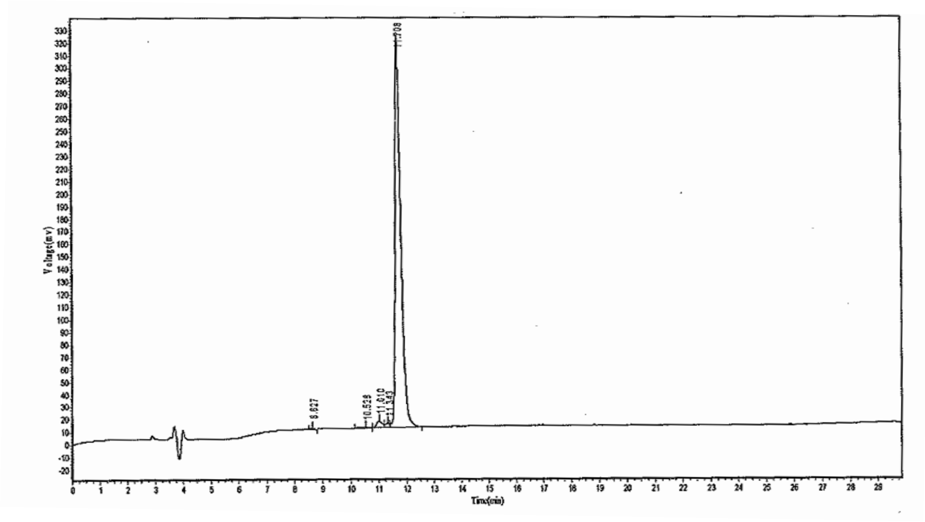

P102:

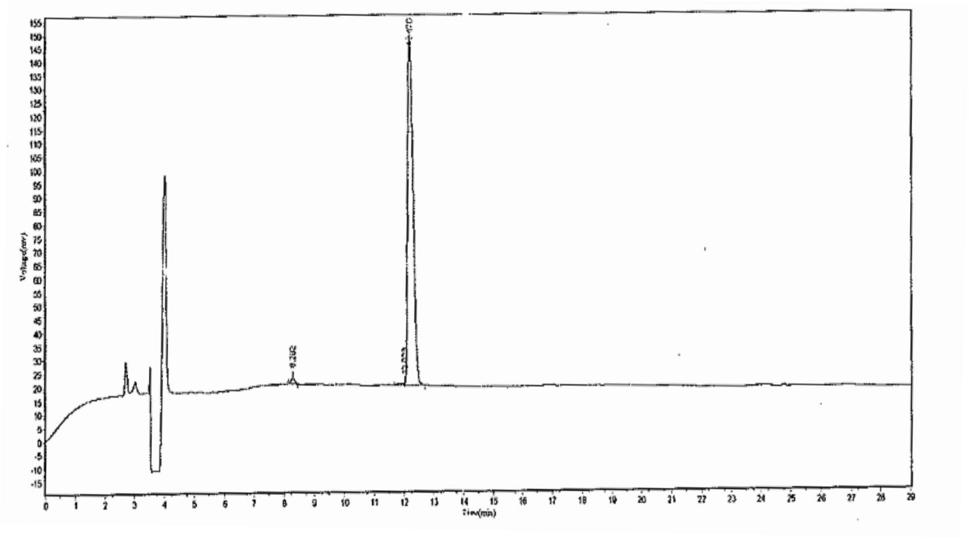

P103:

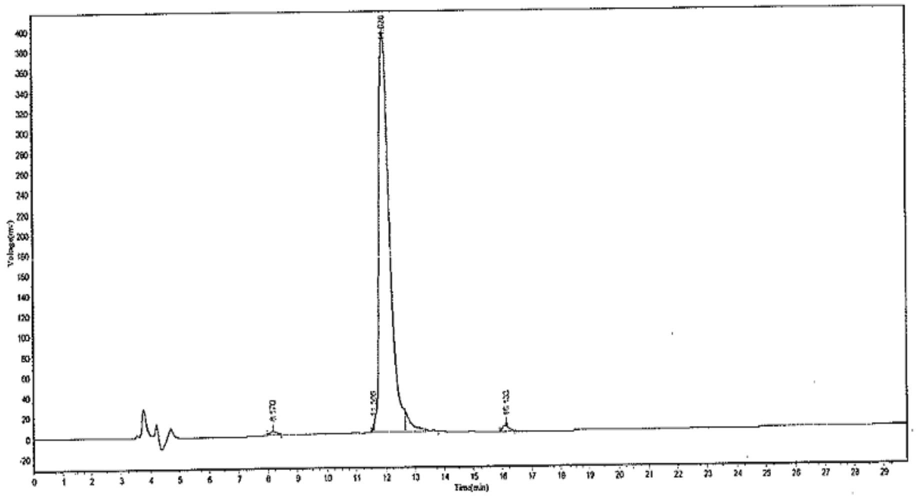

P104:

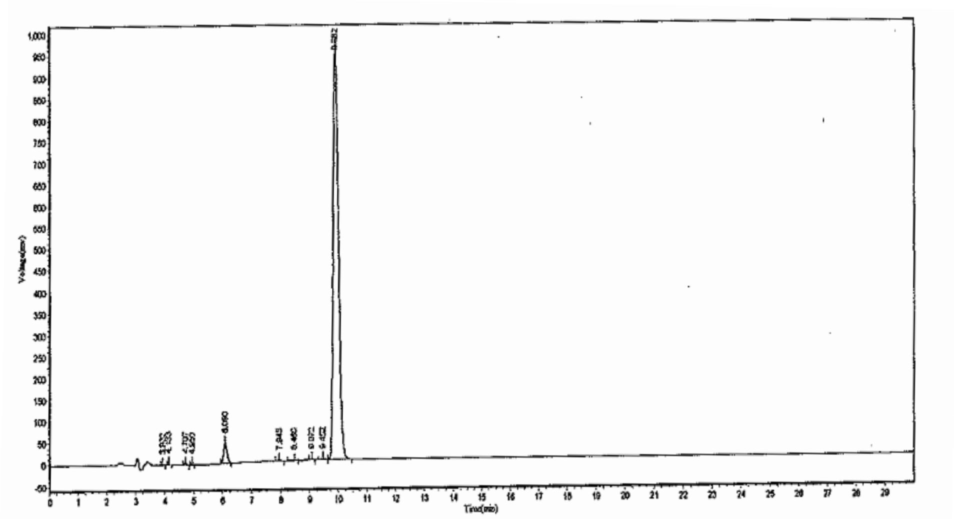

P105:

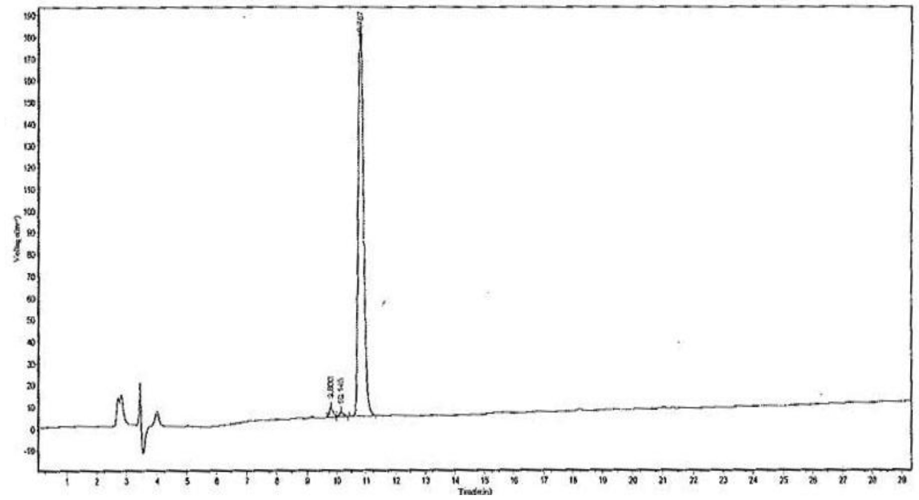

P106:

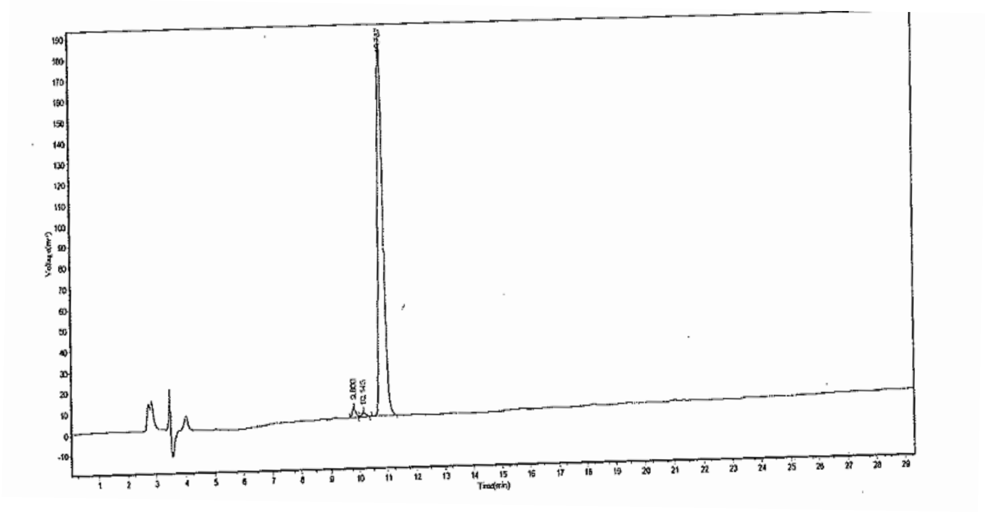

P107:

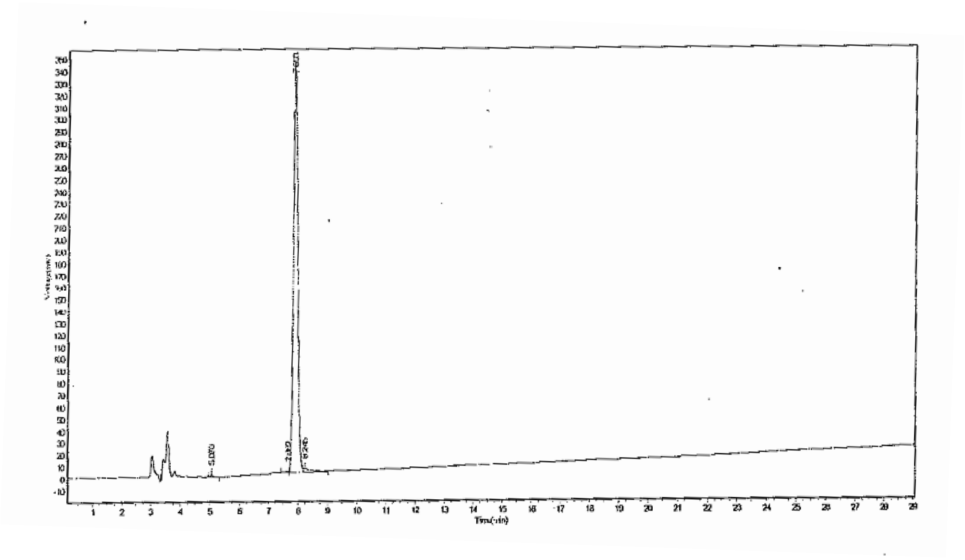

P108:

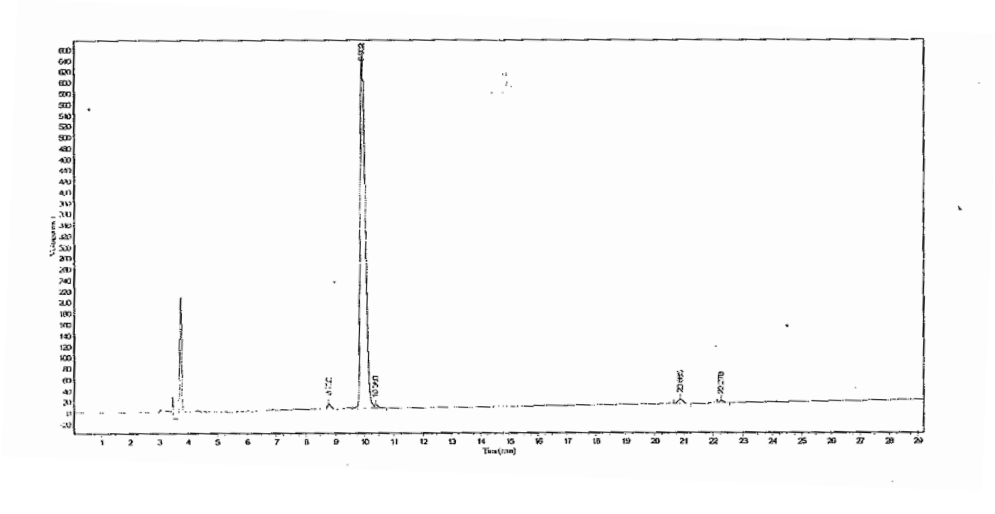

P109:

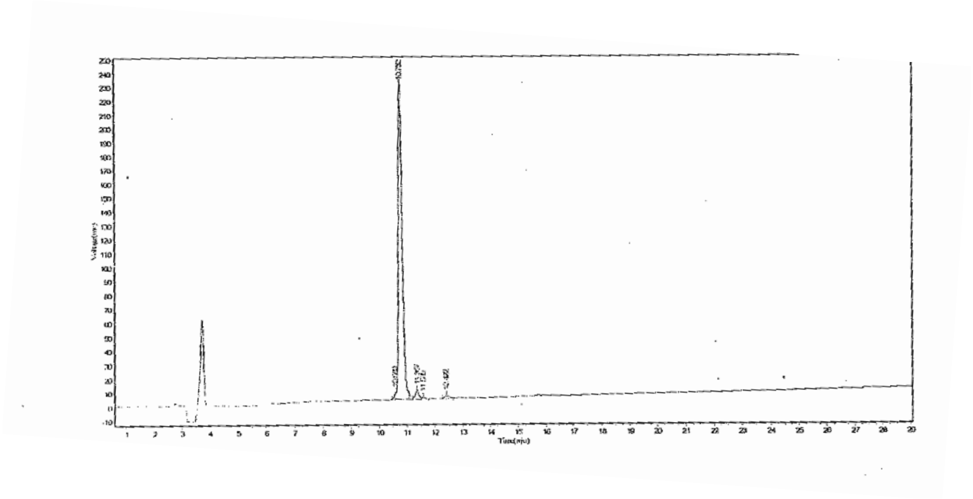

P110:

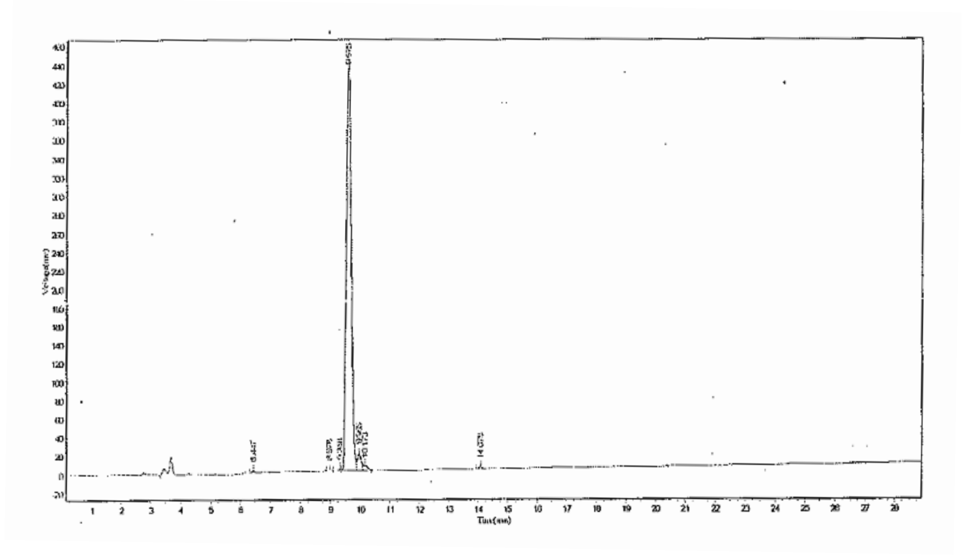

P111:

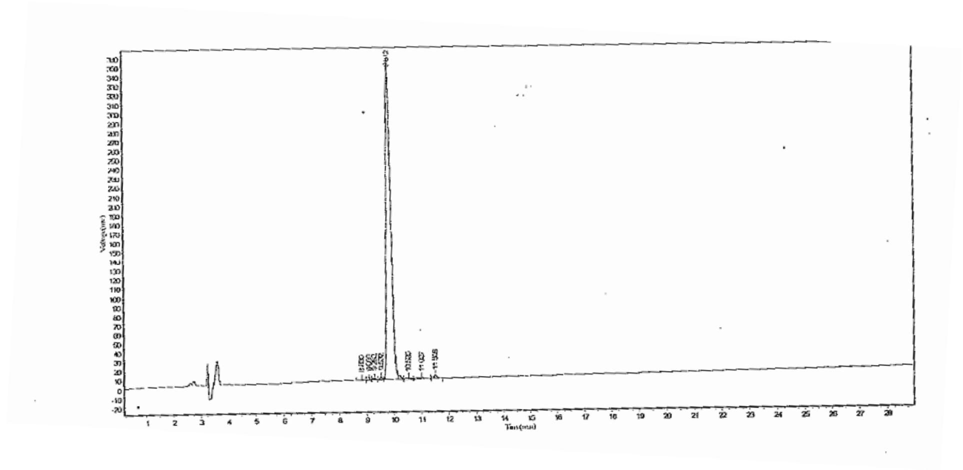

P112:

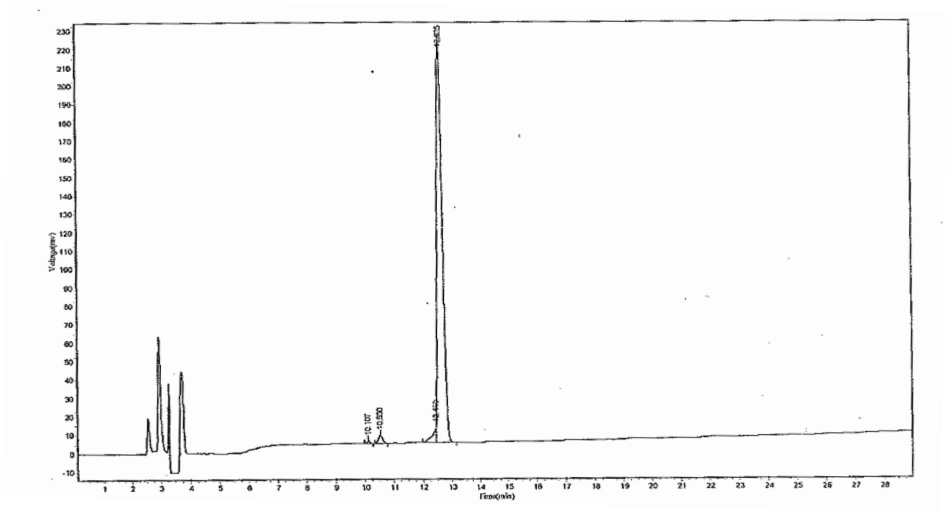

P113:

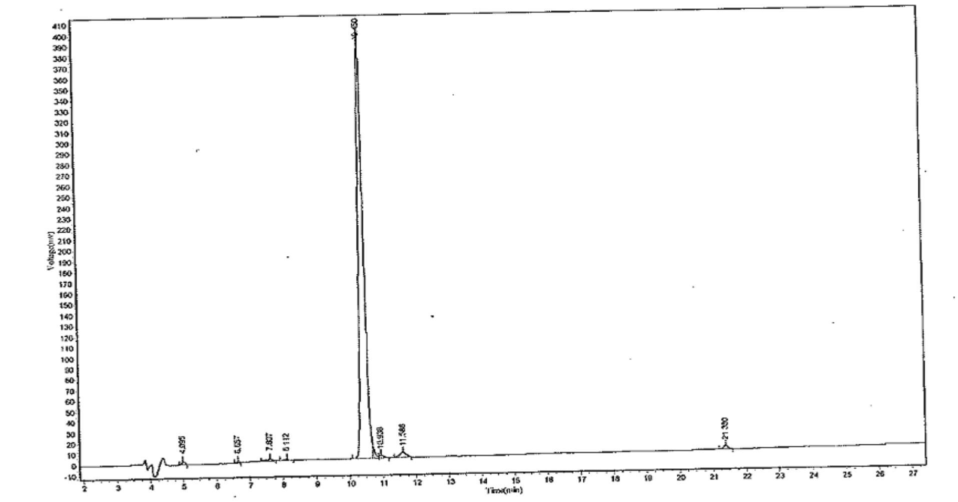

P114:

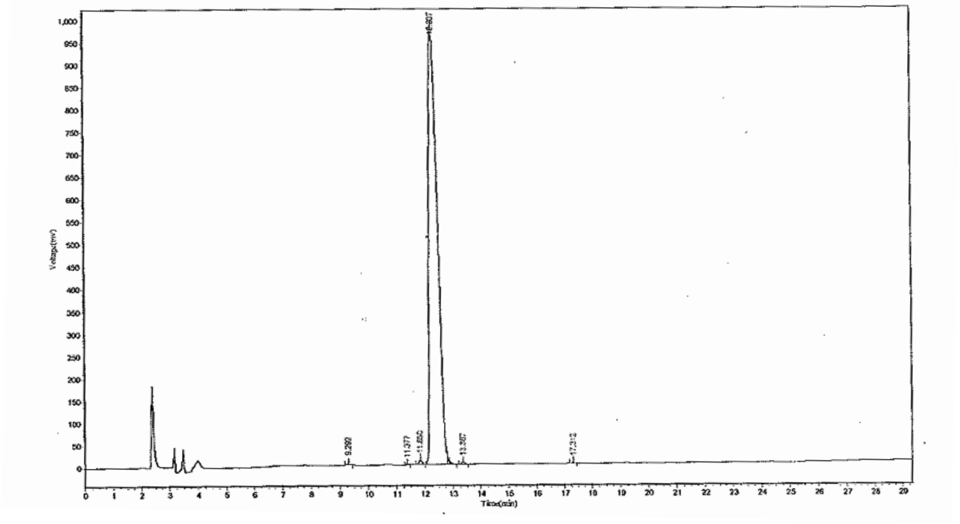

P115:

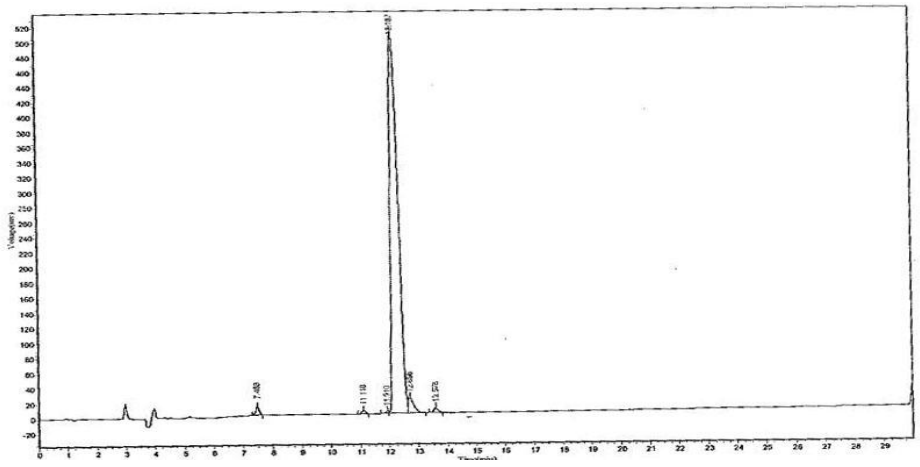

P116:

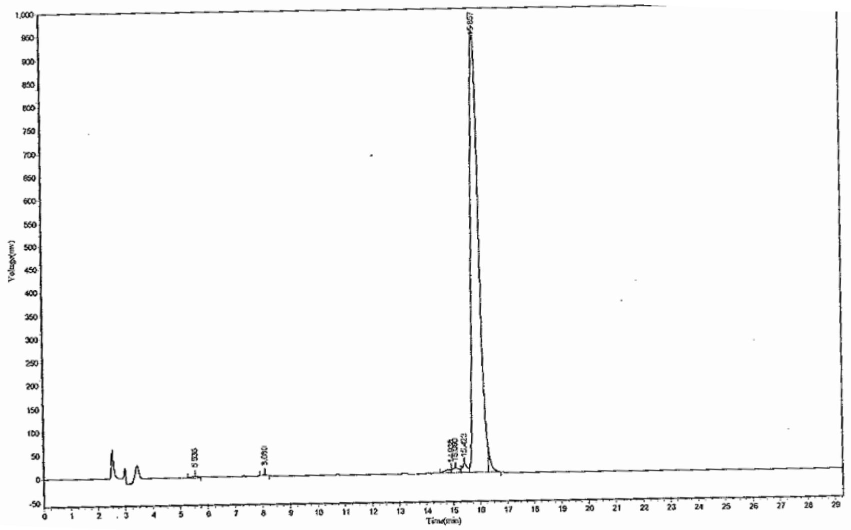

P117:

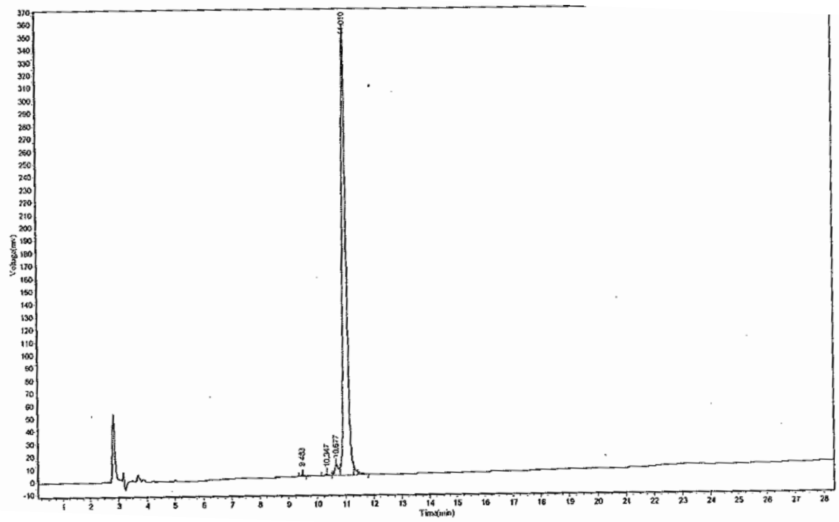

P118:

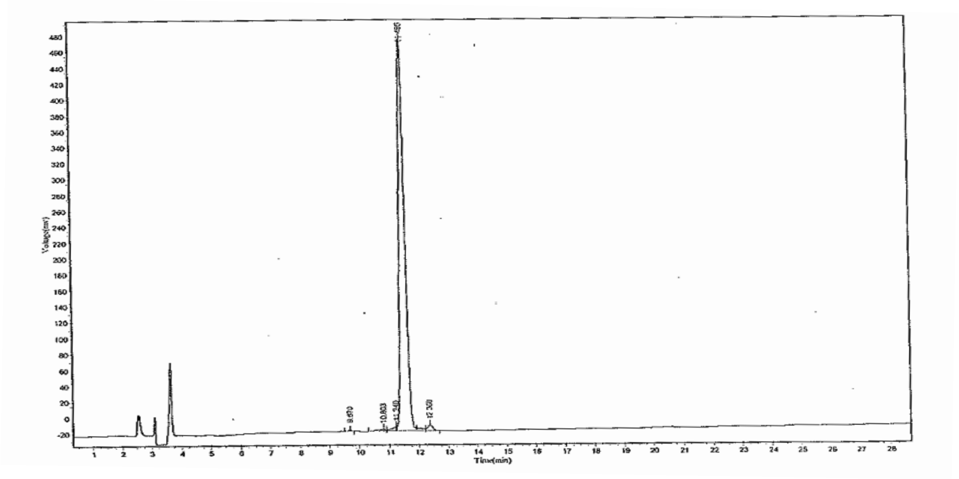

P119:

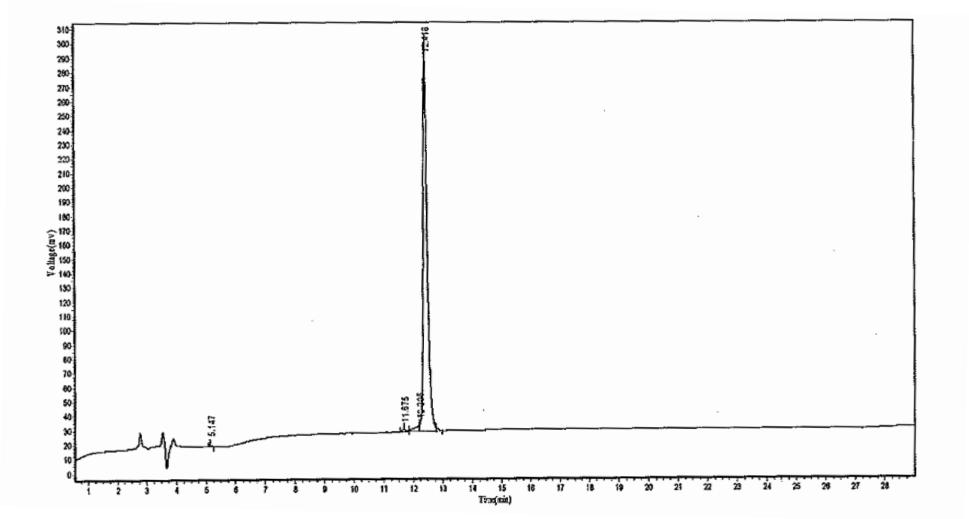

P120:

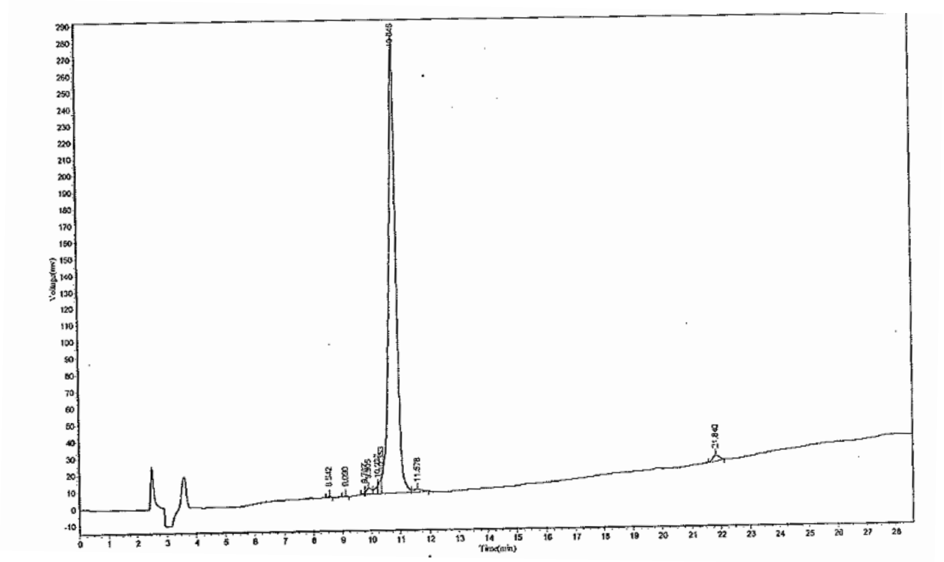

P121:

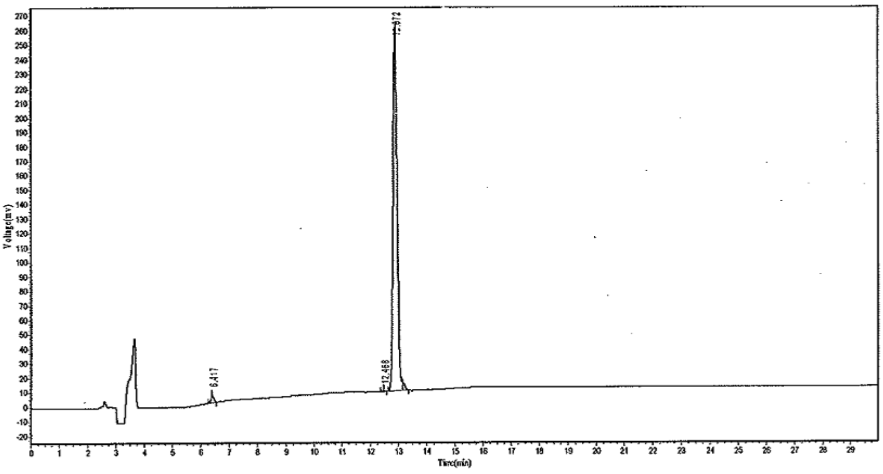

P122:

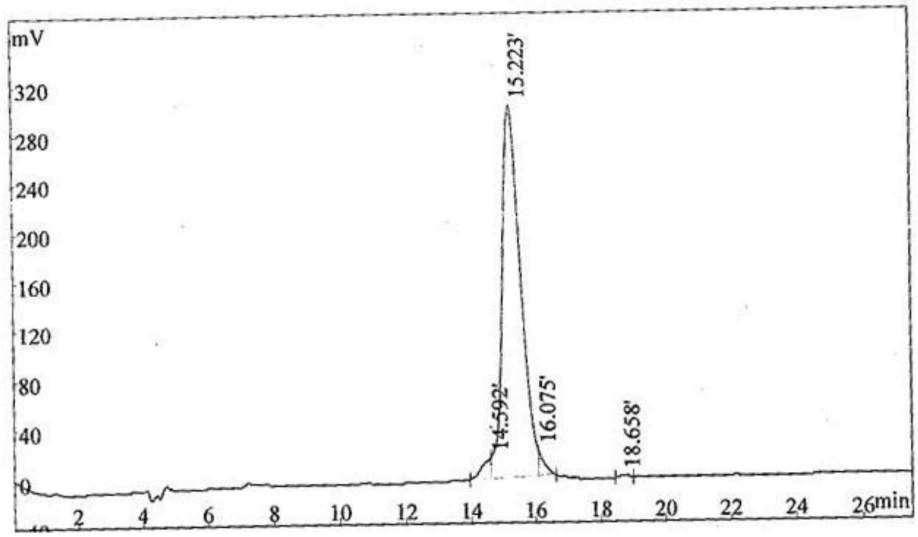

P123:

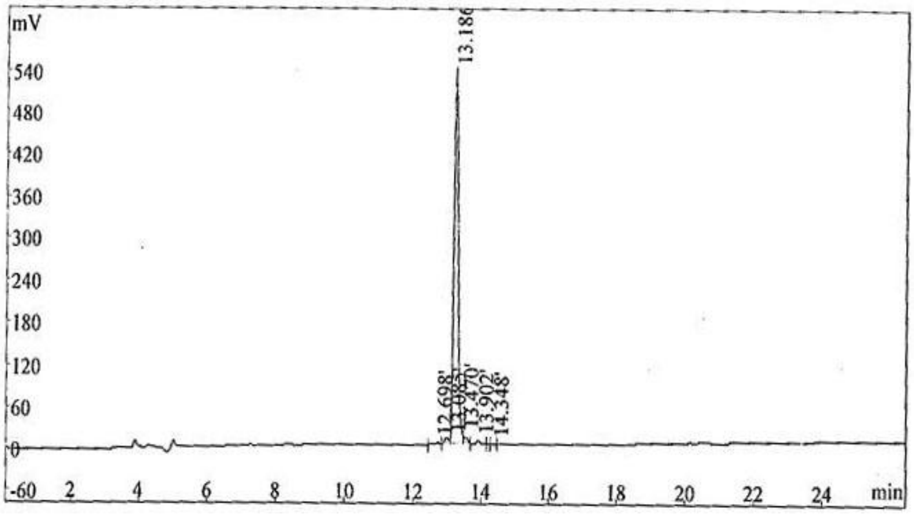

P124:

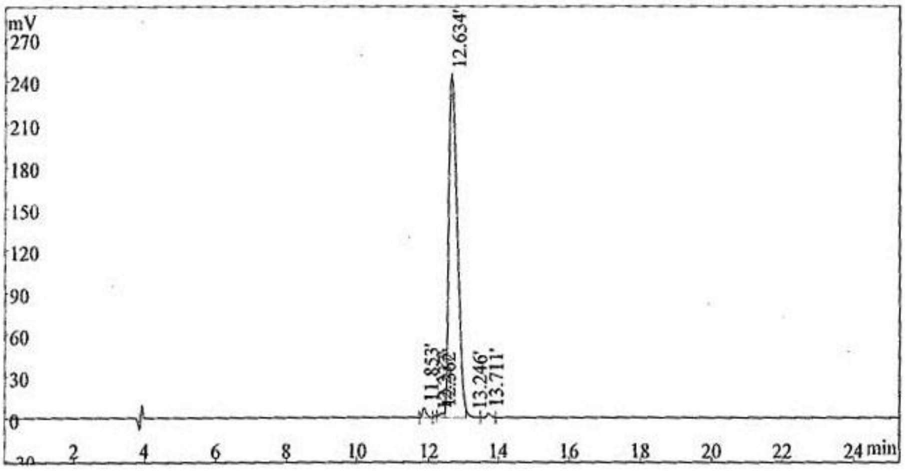

P125:

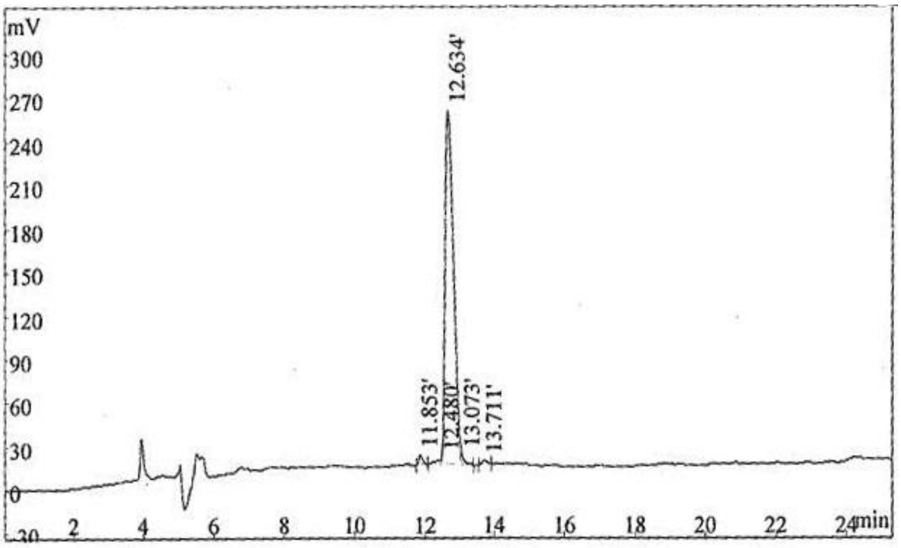

P126:

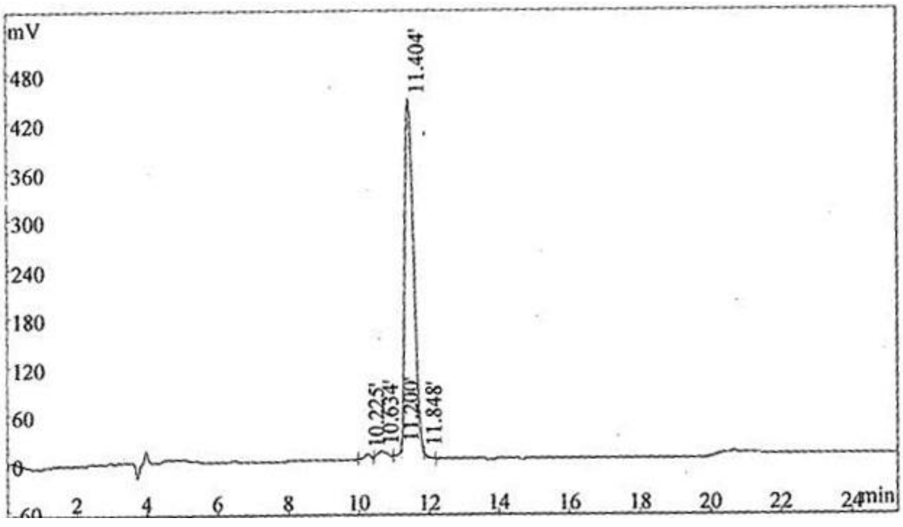

P127:

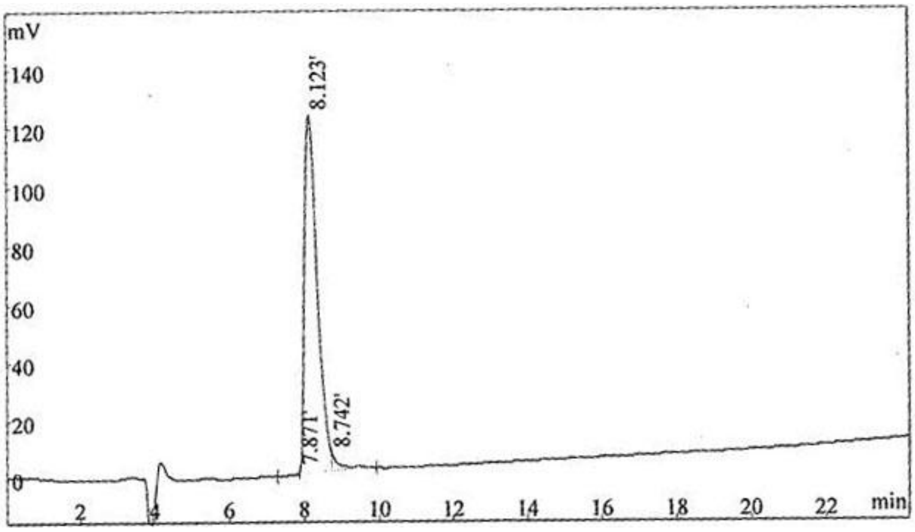

P128:

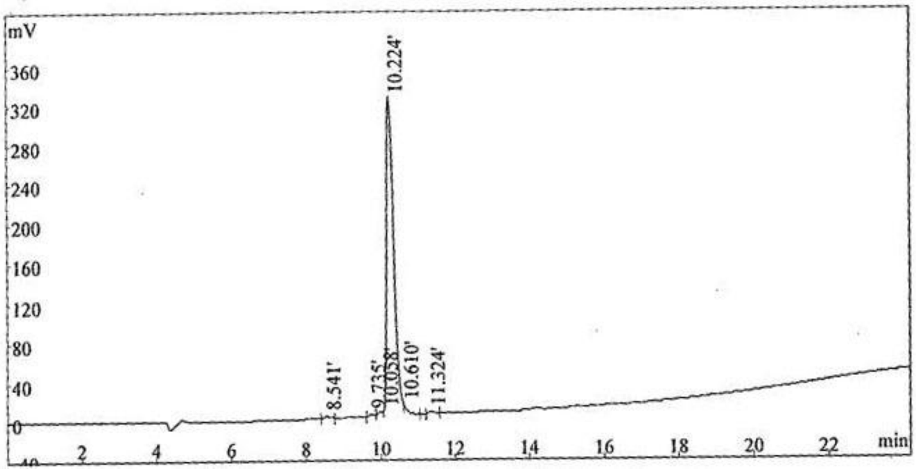

P129:

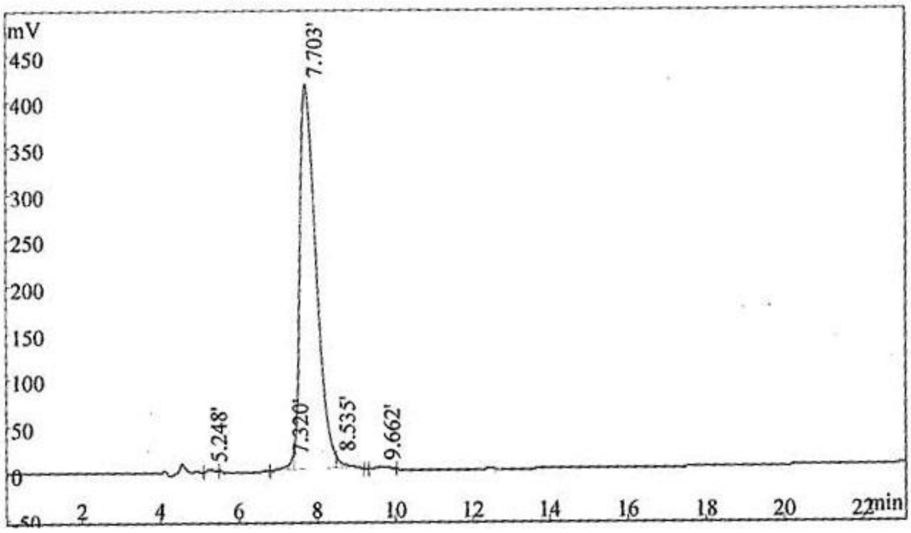

P130:

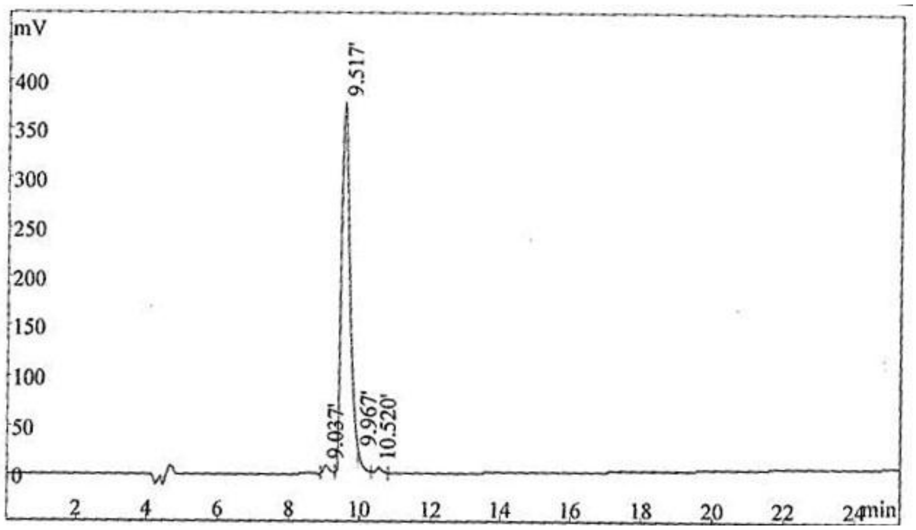

P131:

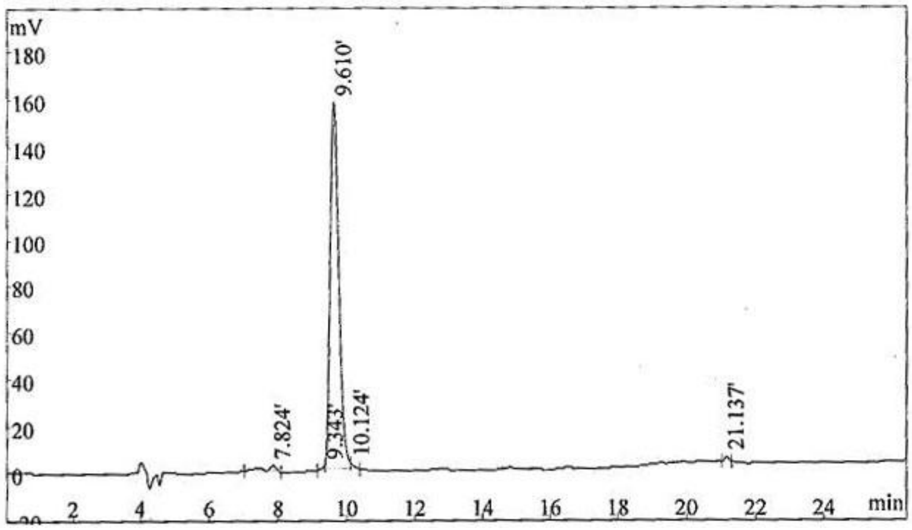

P132:

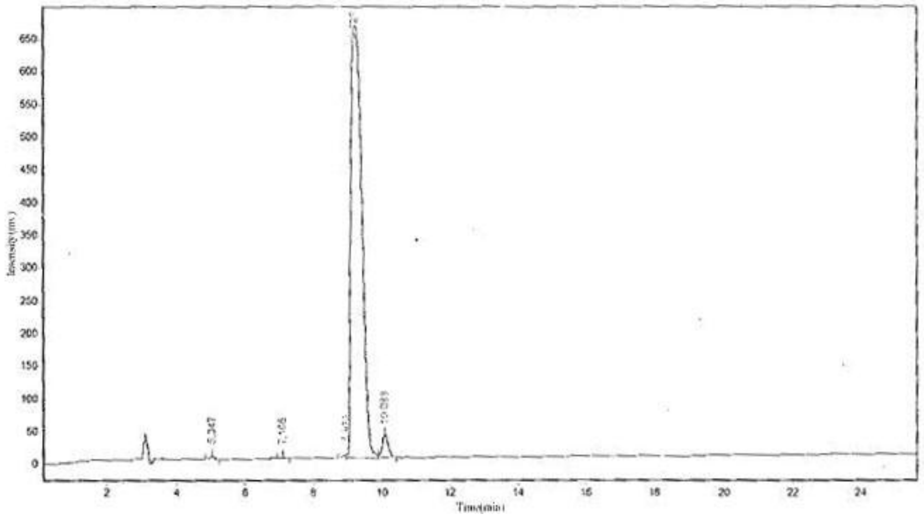

P133:

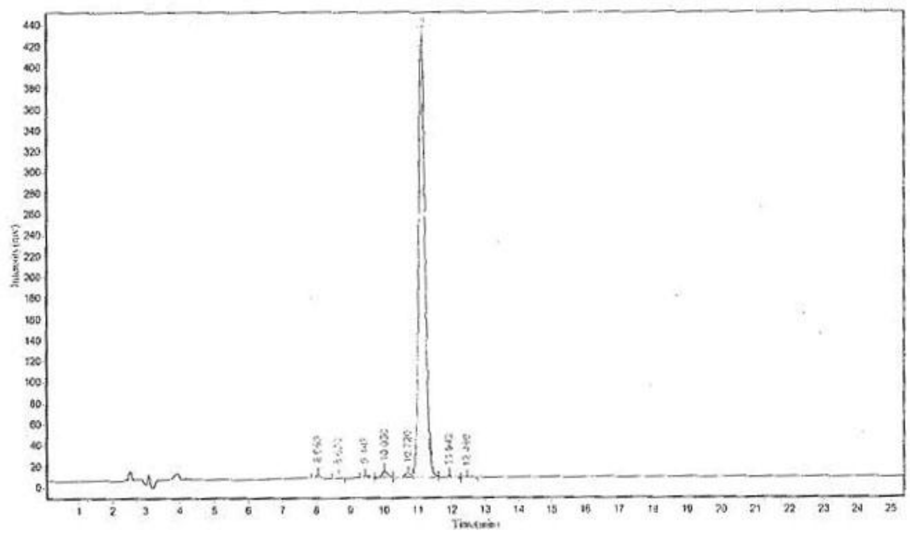

P134:

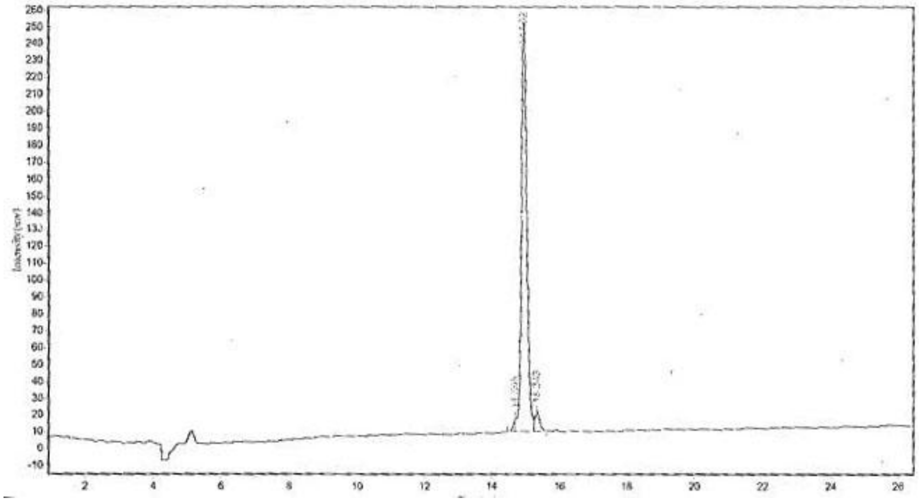

P135:

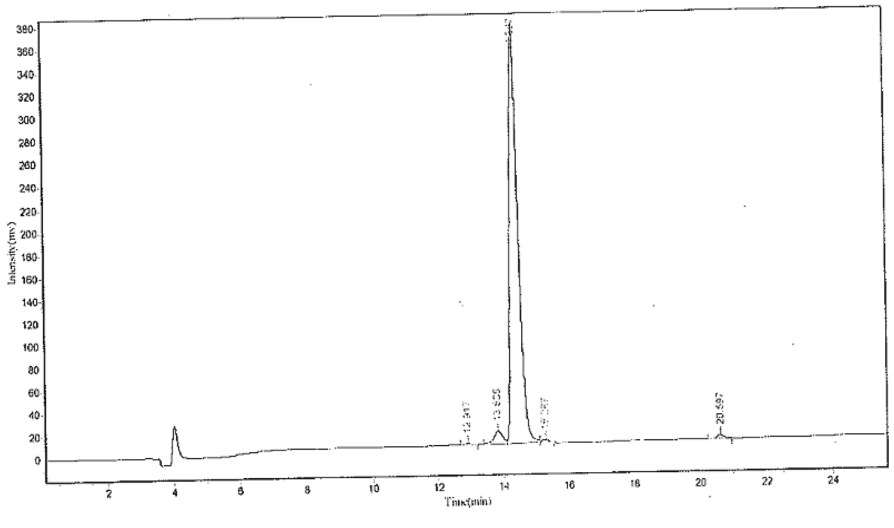

P136:

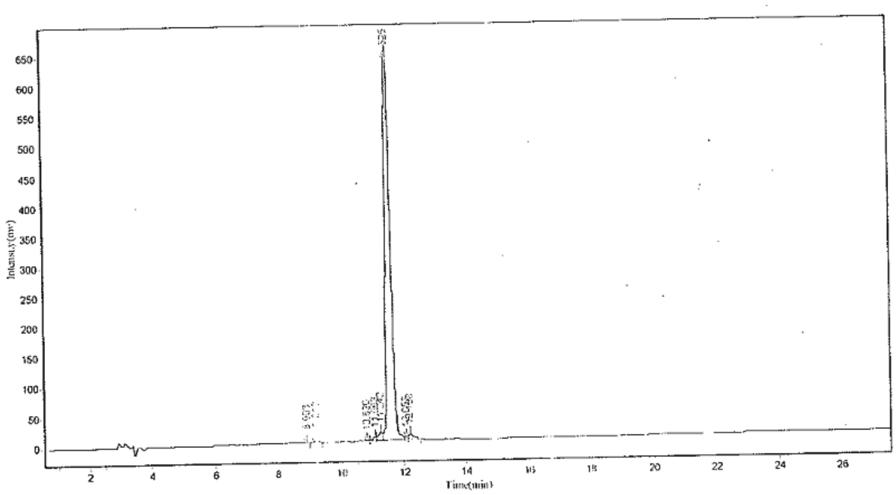

P155:

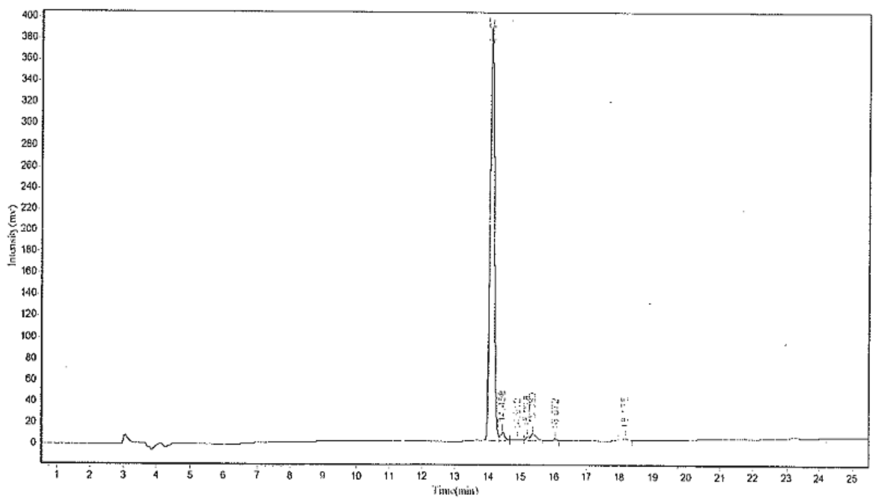

P156:

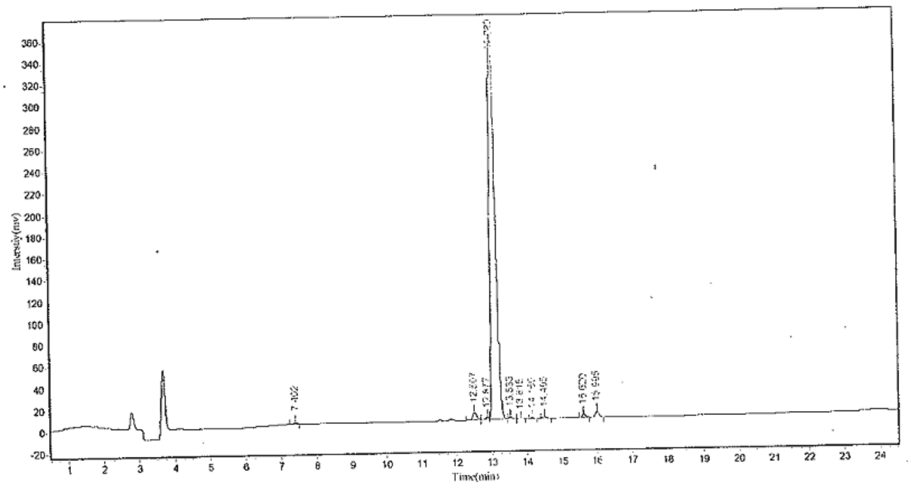

P157:

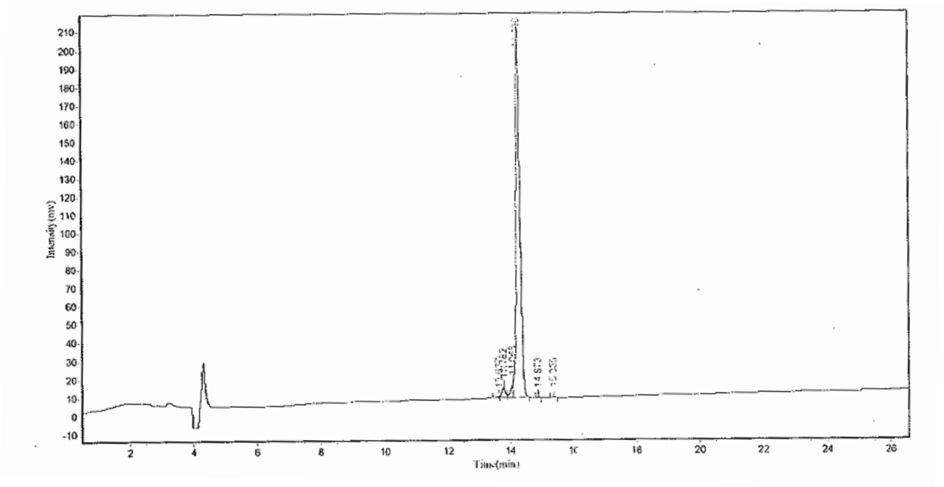

P158:

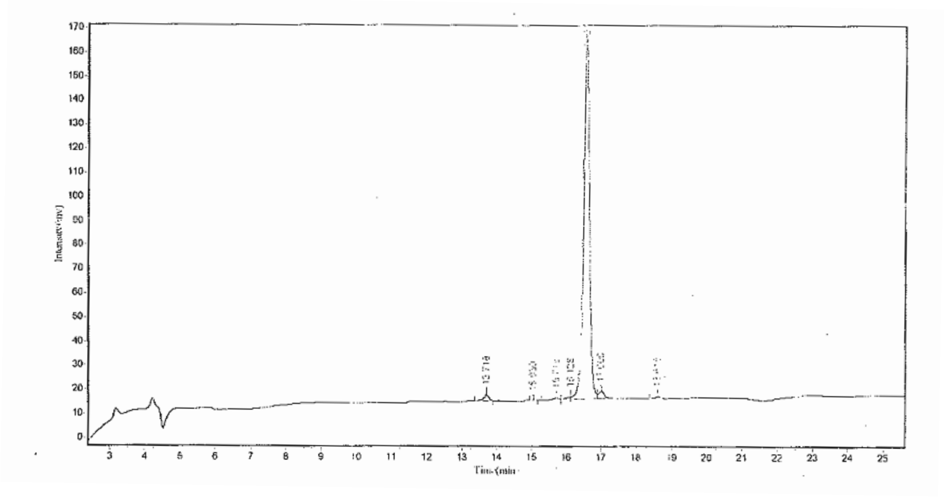

P159:

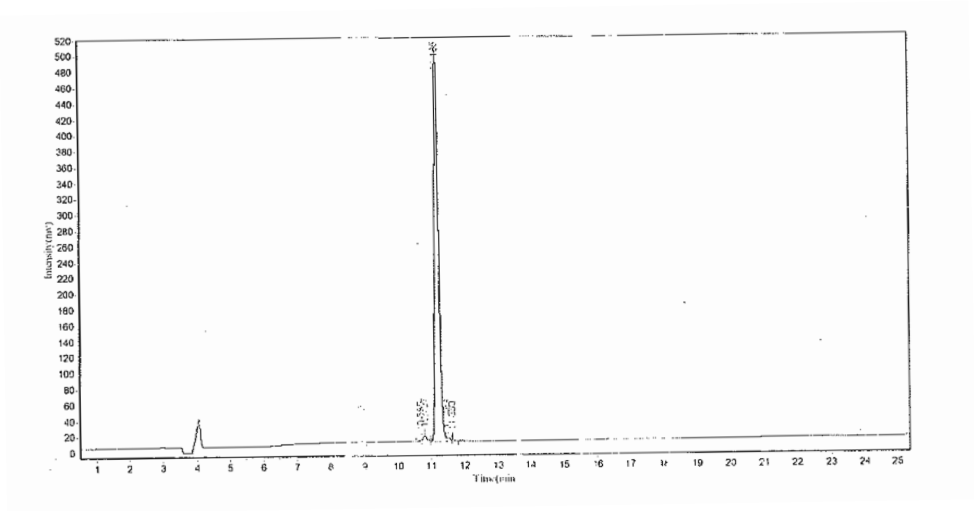

P160:

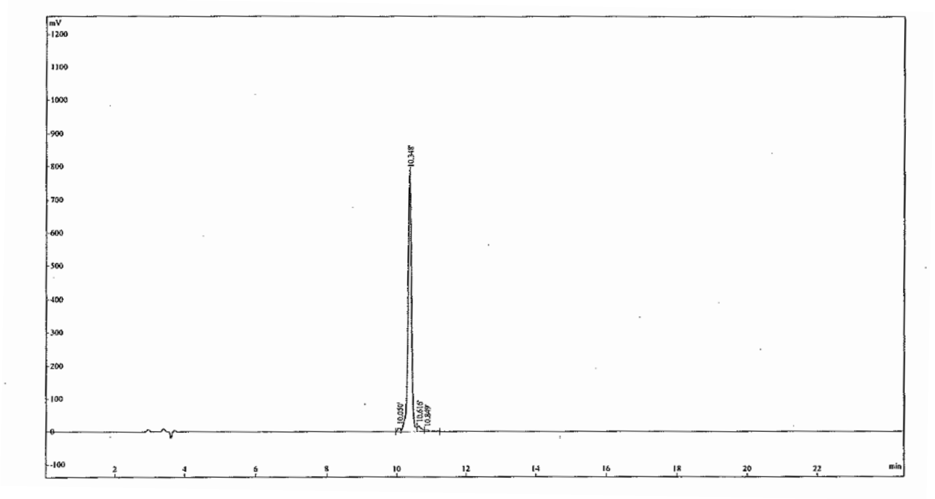

P161:

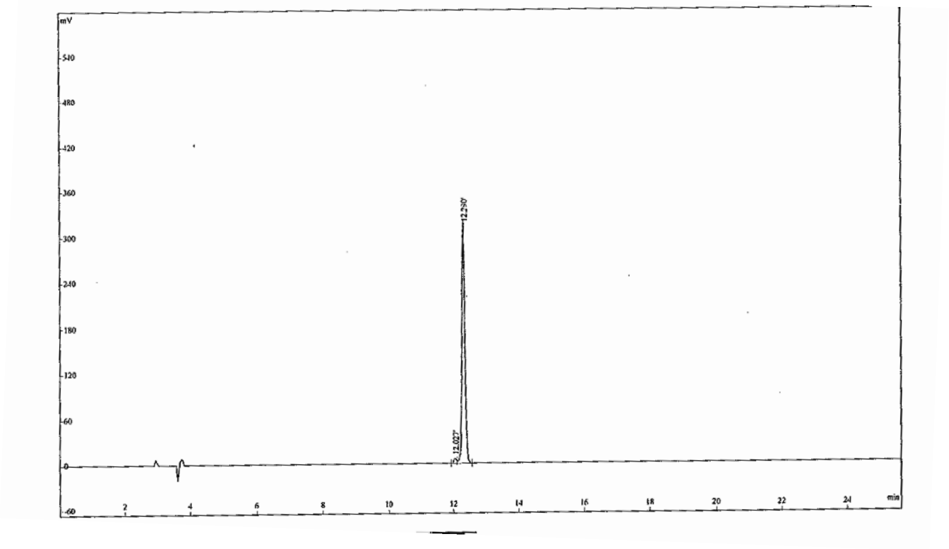

P162:

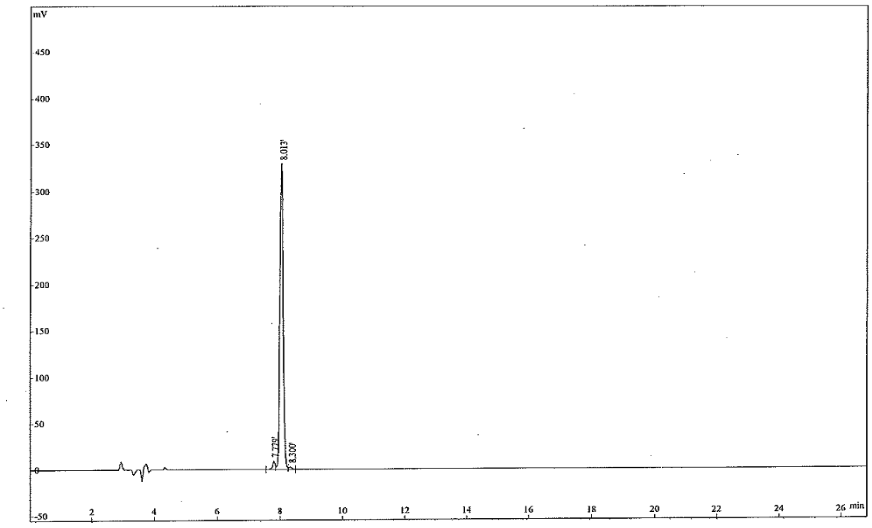

P163:

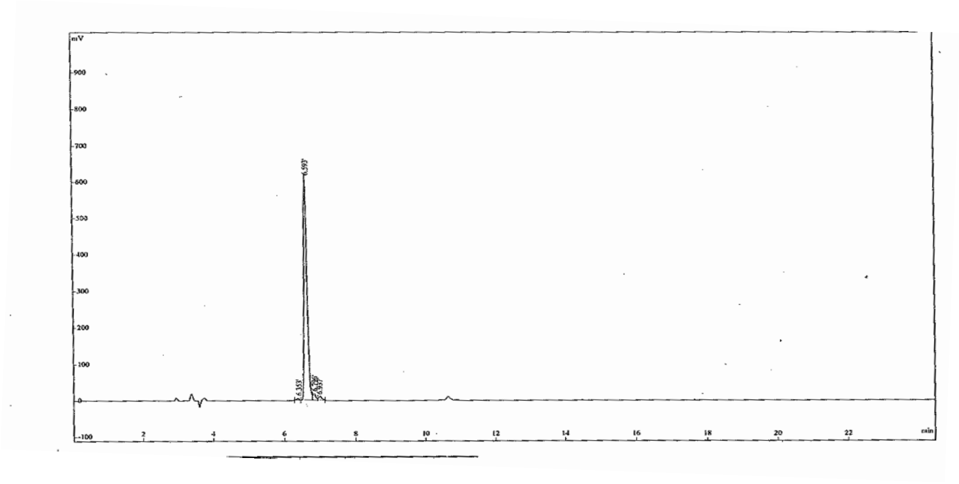

P164:

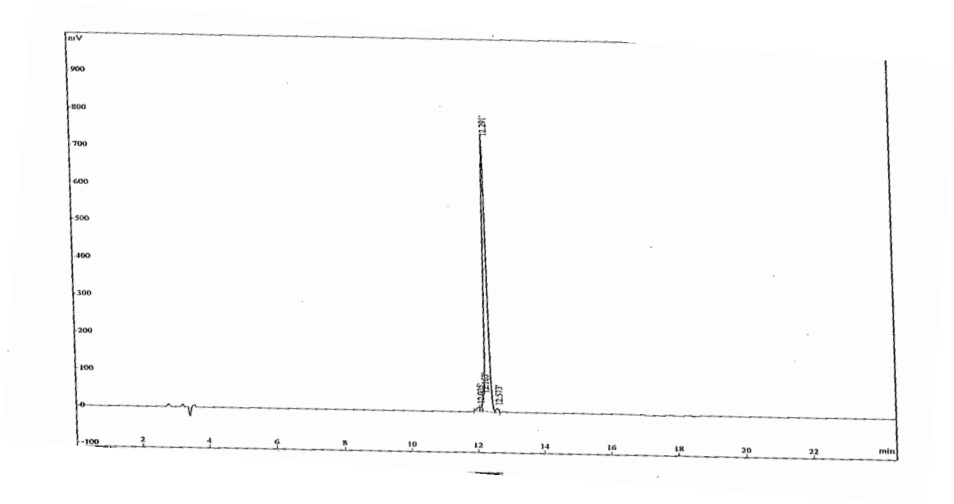

P165:

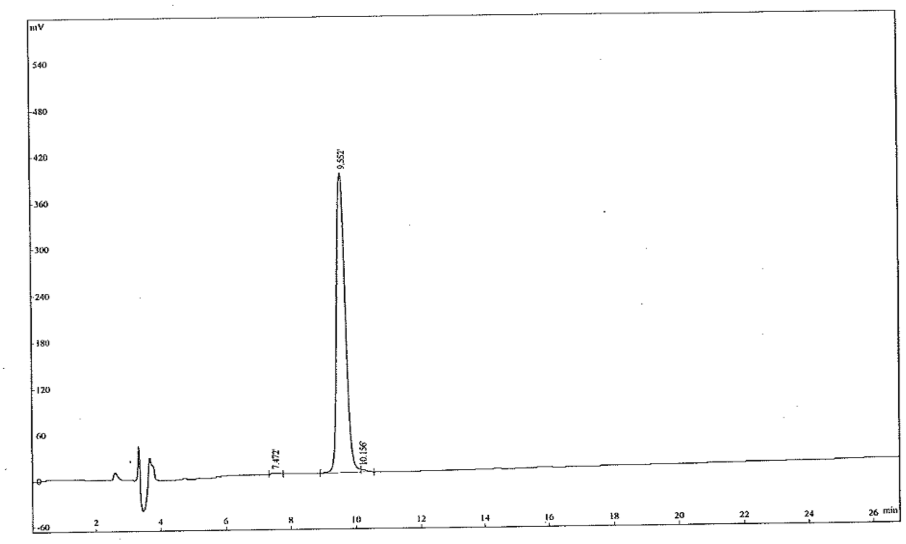

P166:

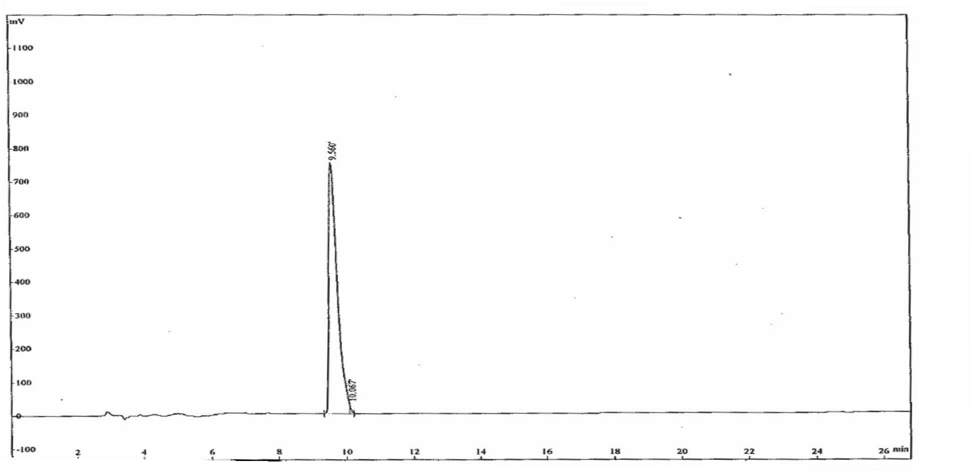

P167:

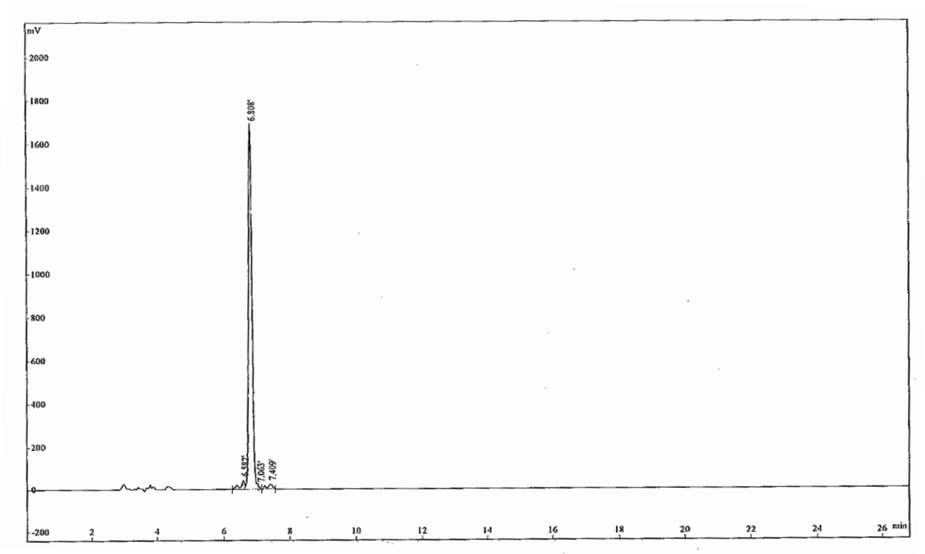

P168:

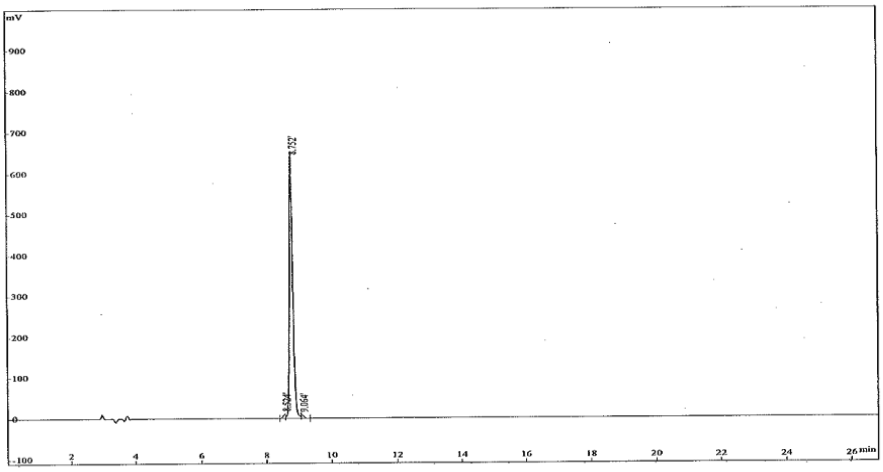

P169:

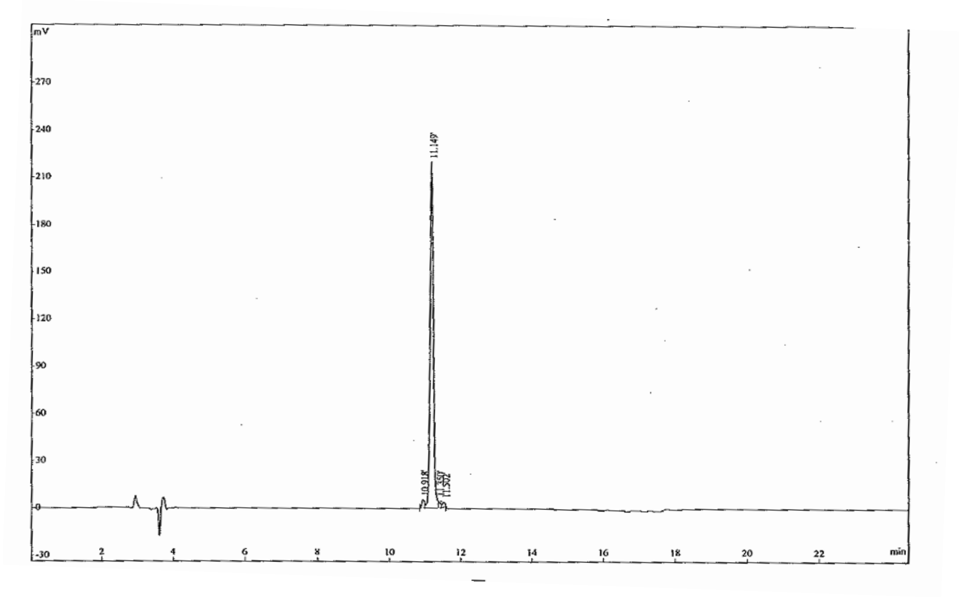

Mart-1(13 peptides, P137~P149)

P137:

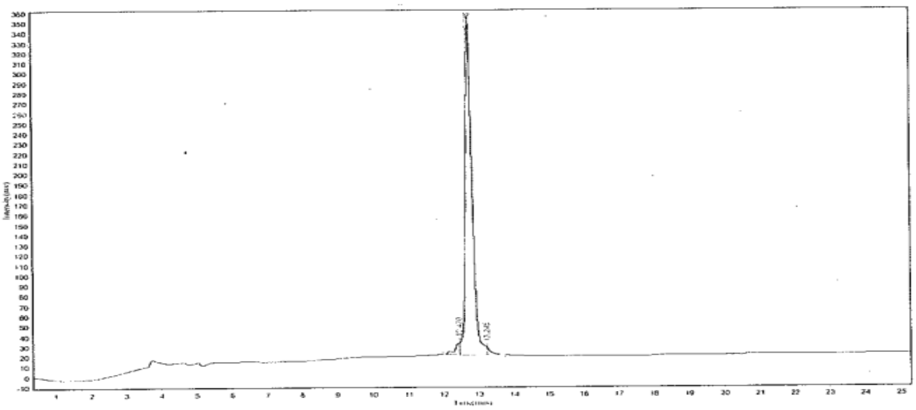

P138:

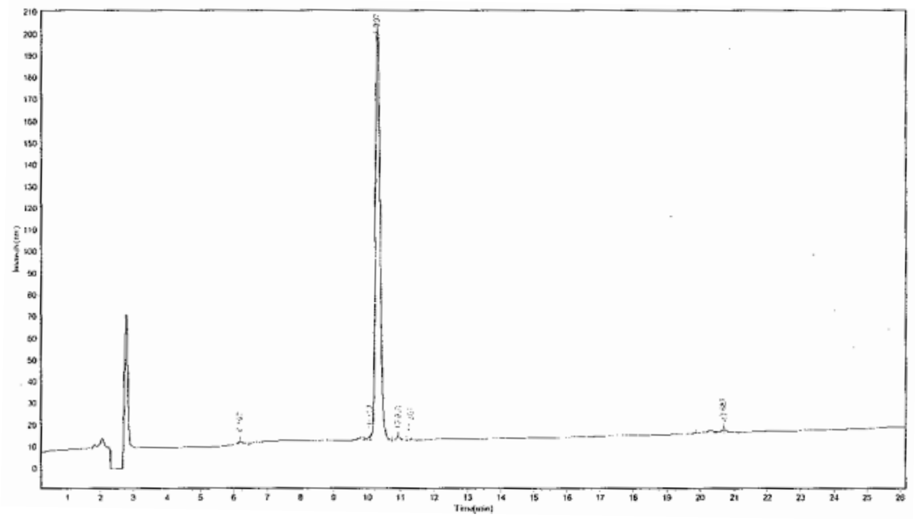

P139:

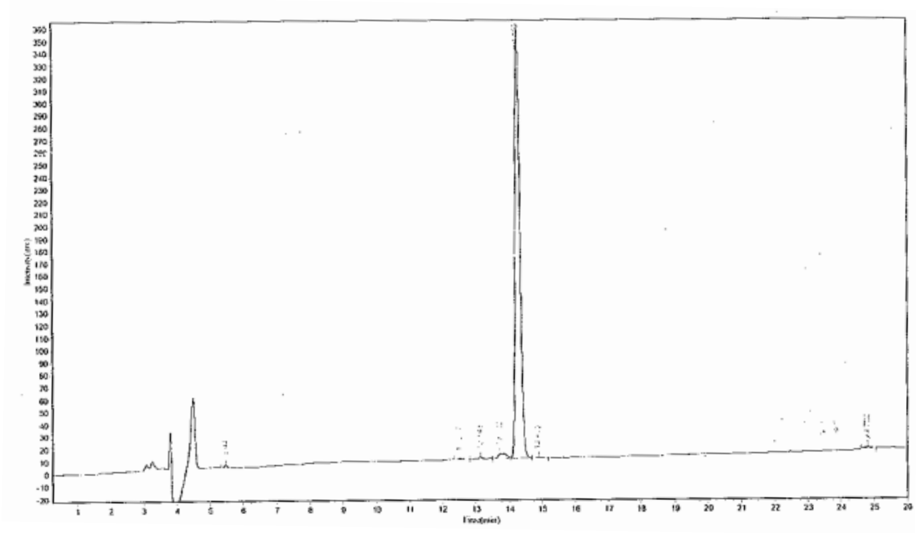

P140:

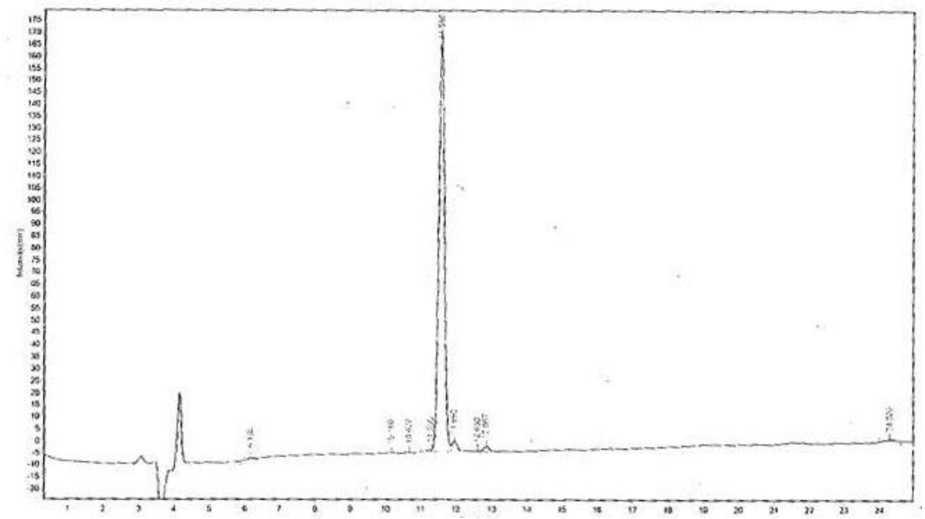

P141:

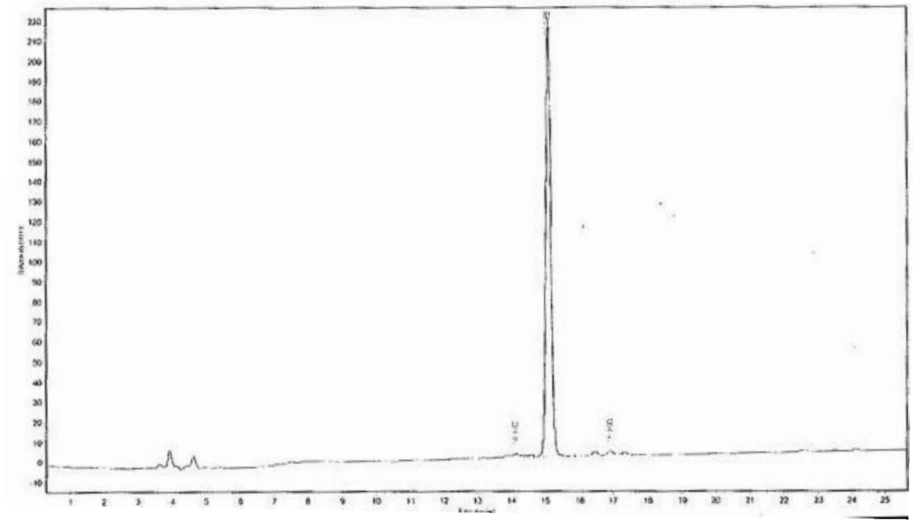

P142:

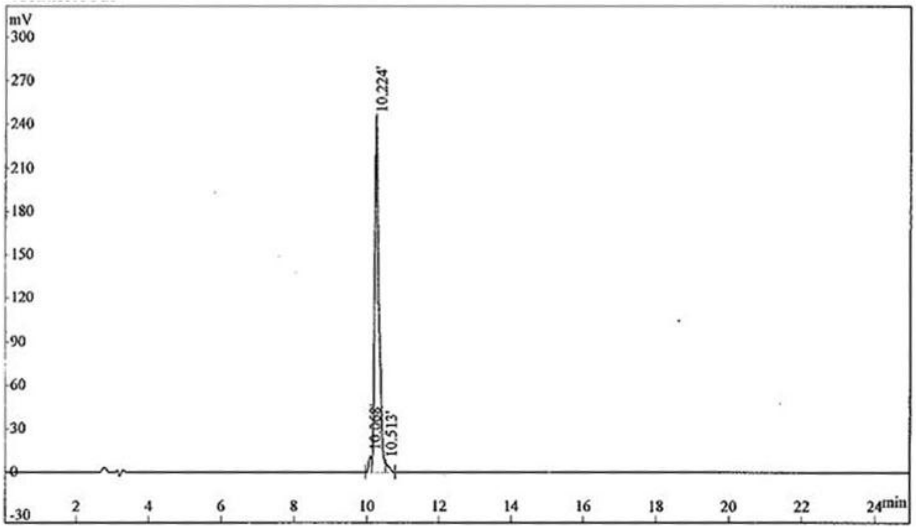

P143:

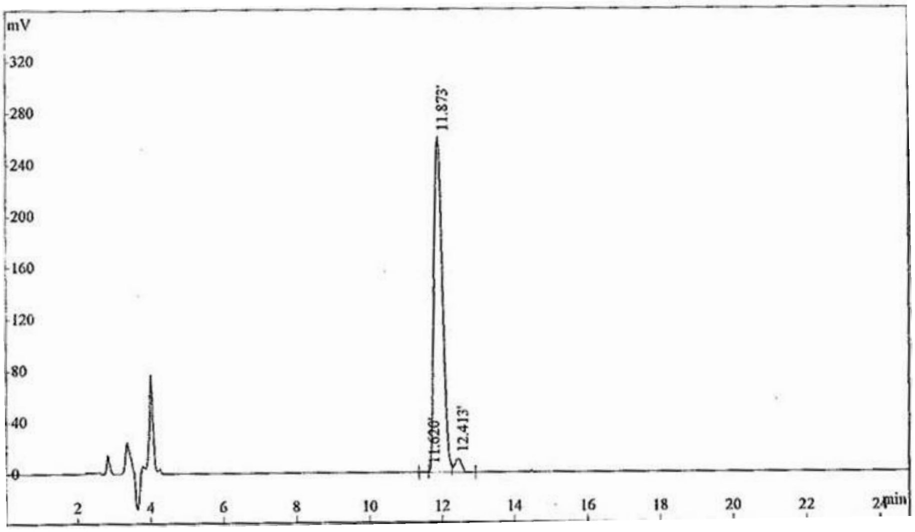

P144:

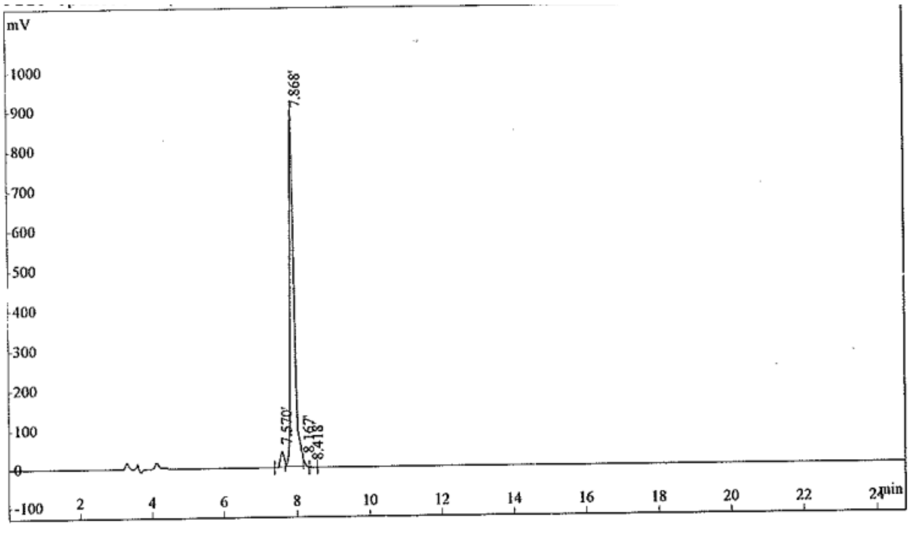

P145:

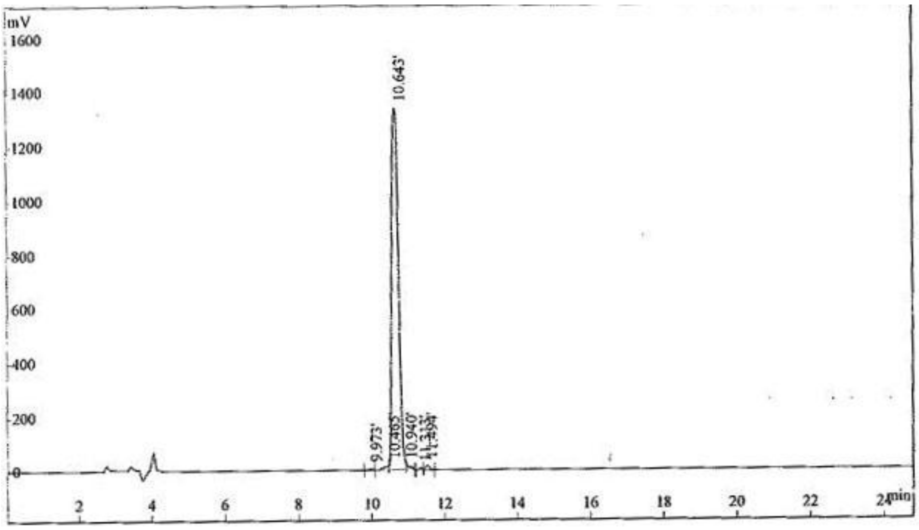

P146:

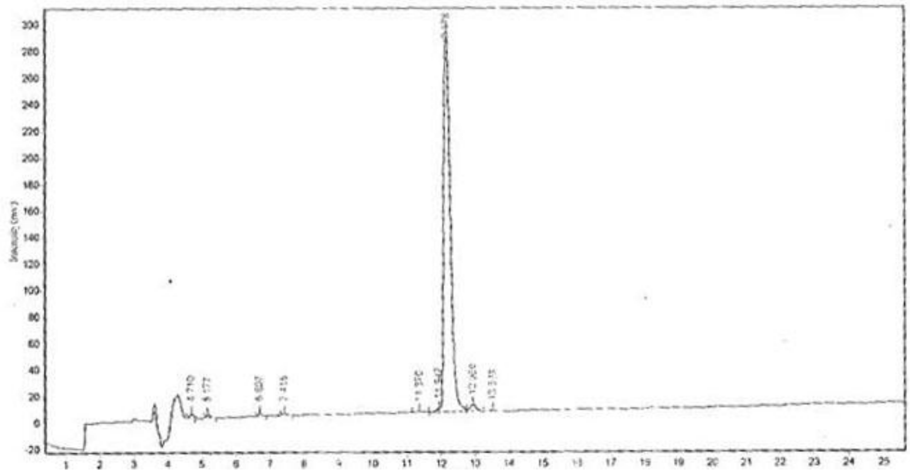

P147:

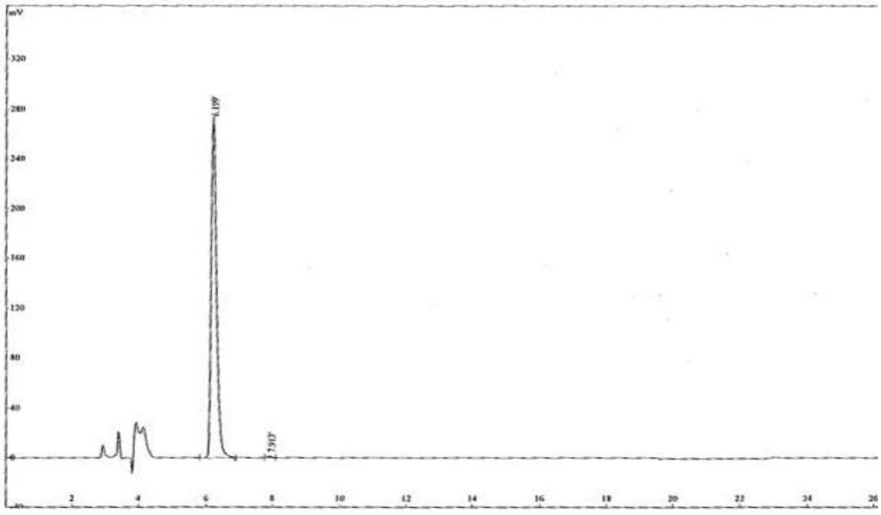

P148:

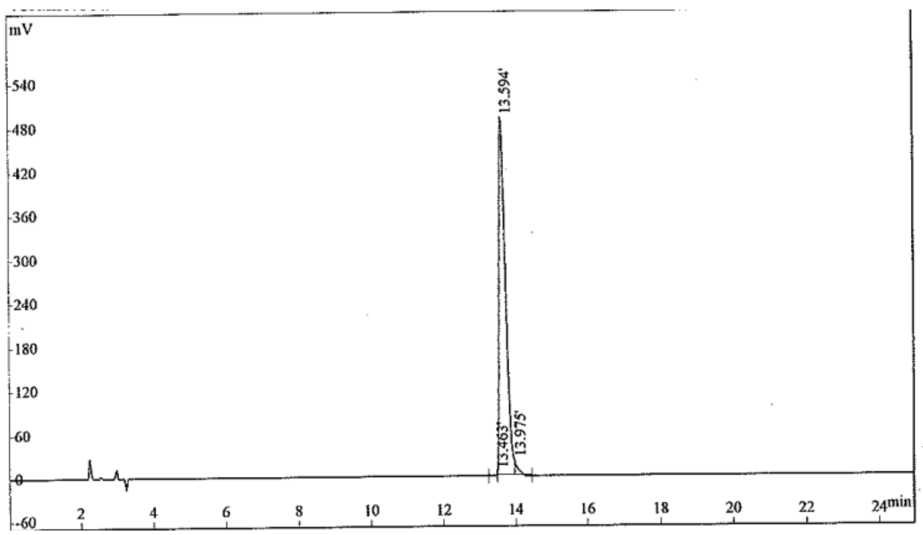
mV
540
480
420
360
300
240
180
120
60
0
-60
2
4
6
8
10
12
14
16
18
20
22
24min
13.594'
13.463'
13.975'

P149:

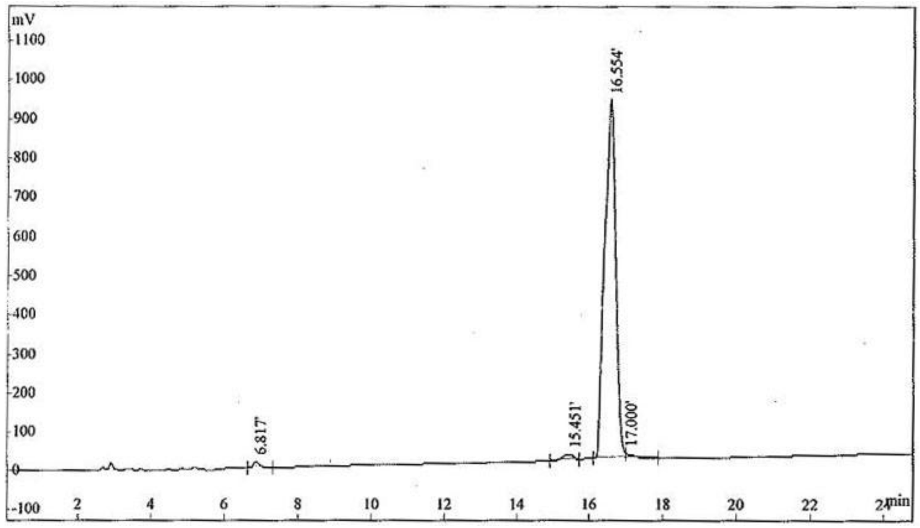
mV
1100
1000
900
800
700
600
500
400
300
200
100
0
-100
2
4
6
8
10
12
14
16
18
20
22
24min
16.554'
6.817'
15.451'
17.000'
